# Supplementary material for: EndoVAscular treatment and ThRombolysis for Ischemic Stroke Patients (EVA-TRISP) registry: basis and methodology of a pan-European prospective ischaemic stroke revascularisation treatment registry
Source: BMJ Open. 2021 Aug 8;11(8):e042211. doi: 10.1136/bmjopen-2020-042211 (PMC8354282; doi:10.1136/bmjopen-2020-042211)
Supplement: Supplementary data [file bmjopen-2020-042211supp001.pdf]

APPENDIX 1: EVA-TRISP Investigators (in alphabetical order by country)

Finland (1): Sami Curtze, Kimmo Lappalainen, Nicolas Martinez-Majander, Jukka Putaala, Gerli Sibolt, Daniel Strbian, Silja Räty, Turgut Tatlisumak, Marjaana Tiainen (Helsinki University Hospital)

France (1): Nicolas Bricout, Marie Bodenant, Regis Bordet, Charlotte Cordonnier, Nelly Dequatre, Hilde Hénon, Didier Leys, Anne-Marie Mendyk (University Lille North de France)

Germany (5): Andreas Kastrup, Panagiotis Papanagiotou (University Hospitals Bremen-Mitte and Bremen-Ost); Tim-Bastian Braemswig, Hebun Erdur, Christian H Nolte, Regina von Rennenberg, Jan F Scheitz, Georg Bohner (Charité-Universitätsmedizin, Berlin); Alex Brehm, Jan Liman, Marios Psychogios (University Medical Center Goettingen); Martin Bendszus, Christian Hametner, Markus Möhlenbruch, Peter A Ringleb (University Hospital Heidelberg); Katharina Feil, Lars Kellert, Clemens Küpper (University Hospital Munich LMU)

Grecce (1): George Ntaios, Dimitrios Sagris, Ioannis Ioannidis George Karagiorgas, Eftychia Kapsalaki, Marianna Vlychou (University of Thessaly)

Israel (1): Jose Cohen, John Gomori, Ronen R Leker (Hadassah-Hebrew University Medical Center, Jerusalem)

Italy (3): Mauro Magoni, Alessandro Pezzini (University Hospital Brescia); Guido Bigliardi, Luca Verganti, Stefano Vallone, Stefania Maffei (University Hospital Modena), Andrea Zini,

Nordanstig A, *et al. BMJ Open* 2022; 11:e042211. doi: 10.1136/bmjopen-2020-042211

Luigi Simonetti, Mauro Gentile, Luigi Cirillo, Ludovica Migliaccio (IRCCS Istituto di Scienze Neurologiche di Bologna)

The Netherlands (1): M Irem Baharoglu, Sophie van den Berg, Paul J Nederkoorn, Yvo B Roos, Fianne HM Spaander, Sanne M Zinkstok, Thomas P Zonneveld, Charles Majoie (Academic Medical Center Amsterdam)

Serbia (1): Mirjana Arsenijevic, Ivana Berisavac, Marko Ercegovac, Dejana R Jovanovic, Visnja Padjen, Predrag Stanarcevic, Maja Stefanovic Budimkic, Tamara Svabic Medjedovic, Ivan Vukasinovic, Vladimir Cvetic (Clinical Centre of Serbia, Belgrade)

Sweden (1): Margareta Abrahamson, Arne Allard, Monica Argus, Anke Brederleu, Erik Ceder, Maria Davidson, Niclas Dehlfors, Dennis Dunker, Torsteinn Gunnarsson, Lukas Holmegaard, Mikael Jerndal, Susanna Johansson, Katarina Jood, Camilla Karlsson, Jan-Erik Karlsson, Birgitta Leiram, Miroslav Malac, Inger Nilsson, Annika Nordanstig, Petra Redfors, Alexandros Rentzos, Turgut Tatlisumak (Sahlgrenska University Hospital, Gothenburg)

Switzerland (5): Leo H Bonati, Stefan T Engelter, Joachim Fladt, Henrik Gensicke, Philippe A Lyrer, Gian Marco De Marchis, Nils Peters, Alexandros Polymeris, Sebastian Thilemann, Christopher Traenka (University Hospital Basel); Marcel Arnold, Urs Fischer, Jan Gralla, Mirjam R Heldner, Hakan Sarikaya, David J Seiffge, Roland Wiest (University Hospital Bern); Olivier Bill, Ashraf Eskandari, Patrik Michel, Gaia Sirimarco (Hospitalier Universitaire Vaudois, Lausanne); Georg Kägi, Johannes Weber (Kantonsspital St. Gallen); Zsolt Kulcsar, Andreas R Luft, Susanne Wegener (University Hospital Zurich)

Nordanstig A, *et al. BMJ Open* 2022; 11:e042211. doi: 10.1136/bmjopen-2020-042211

APPENDIX 2. EVA-TRISP DATABASE DOMAINS AND ITEMS

The exact list of available variables may slightly differ between centers according to the judgment of local ethics committees.

Patient history

- Age
- Sex
- Pre-stroke mRS
- Independent prior to stroke (Y/N)
- Risk factor atrial fibrillation present and known (Y/N)
- Risk factor diabetes present and known (Y/N) (Y/N)
- Risk factor hypertension present and known(Y/N)
- Risk factor hypercholesterolemia present and known (Y/N)
- Risk factor coronary artery disease present and known(Y/N)
- Risk factor prior ischemic stroke (clinical diagnosis) (Y/N)
- If prior ischemic stroke, prior treatment with IVT/EVT (Y/N)
- Risk factor current smoking (or stopped <2y) (Y/N
- Pre-IVT use of statins (Y/N as well as name and dosage)
- Pre-IVT use of antihypertensive (Y/N as well as name and dosage)
- Pre-IVT use of antiplatelets (Y/N as well as name and dosage)
- Pre-IVT use of anticoagulants (Y/N as well as name and dosage)
- All medications (name and dosage as free text)

Nordanstig A, *et al. BMJ Open* 2022; 11:e042211. doi: 10.1136/bmjopen-2020-042211

Pre-hospital

- Date of stroke onset (last seen well)
- Time of stroke onset (last seen well)
- Date of stroke onset (first seen sick)
- Time of stroke onset (first seen sick)
- Stroke onset witnessed? (Y/N)
- Wake-up stroke? (Y/N)
- Epileptic seizure at stroke onset (Y/N)

Admission

- Date of hospital arrival
- Time of hospital arrival
- Was patient transferred from another hospital? (Y/N)
- IVT administrated at other hospital? (Y/N)
- NIHSS at admission
- Systolic blood pressure on admission [mmHg]
- Diastolic blood pressure on admission [mmHg]
- Weight (exact / estimated)
- Height (exact / estimated)
- Glucose on admission [mmol/l]
- Creatinine on admission [umol/l]

Nordanstig A, *et al. BMJ Open* 2022; 11:e042211. doi: 10.1136/bmjopen-2020-042211

- International Normalized Ratio prior to IVT (point of care test / actual lab value)
- If direct oral anticoagulants (DOAC), specific essay/level of DOAC
- Platelets on admission x10e9 [/l]
- Hemoglobin on admission [g/dl]
- Leukocytes on admission x10e9 [/l]
- C-Reactive Protein on admission [mg/l]

Acute interventions

- Type of intervention (IVT, bridging, EVT)
- Date of IVT administration
- Time of IVT administration
- Dosage of rtPA IVT (0.6 mg/kg, 0.9 mg/kg, <50% of dose or complete)
- Angioedema related to IVT (Y/N)
- Exclusion criteria IVT (received IVT, delays, major prestroke handicap, imaging contraindication, bleeding risk, stroke missed/uncertain, other)
- Exclusion criteria IVT [free text]
- Date of EVT groin puncture
- Time of EVT groin puncture
- Intra-arterial thrombolytic drug (Y/N)
- Name and dosage of intra-arterial thrombolytic drug [free text]
- General anesthesia during EVT (Y/N)

Nordanstig A, *et al. BMJ Open* 2022; 11:e042211. doi: 10.1136/bmjopen-2020-042211

- Type of endovascular treatment (none, stent retriever, aspiration, distal retriever, distal aspiration, Balloon angioplasty, permanent intracranial stent, extracranial stent, other [combination possible]
- Number of attempts for each treatment
- Name of device(s)
- Tandem stenosis/occlusion present (Y/N)
- Extracranial thrombectomy (no, ICA, VA)
- Extracranial permanent stent (no, ICA, VA)
- Only attempt to perform EVT
- EVT stopped early because (initiated, but access-to-clot-problems, tried, but artery already recanalized, other)
- EVT stopped early reason [free text]
- EVT complications (no, vessel perforation, vasospasm, dissection, SAH/ICH, device detachment/misplacement, embolization to new territory, access-site complications, early reocclusion, other [free text] [combination possible]
- Time of EVT end of procedure

Imaging

- Type of baseline image (none, CT, MR)
- Date of baseline image
- Time of baseline image

- Territory of infarction (ICA, MCA, ACA, PCA, Cerebellum, Brainstem [combination possible]
- Side of infarction (left anterior circulation, right anterior circulation, posterior circulation [combination possible]
- Anterior circulation ASPECTS on baseline image 0-10
- Posterior circulation ASPECTS on baseline image 0-10
- Early ischemic changes in suspected area (focal parenchymal hypoattenuation, loss of gray-white matter differentiation, focal edema manifested by sulcal or ventricular effacement) (Y/N)
- Occluded vessel with hyperdense artery sign (-> column CD) (Y/N)
- Type of baseline angiography (none, CTA, MRA)
- Site of main intracranial occlusion on baseline angiography (none, ICA-I, ICA-L/T, prox M1, distal M1, M2, ACA, PCA, BA, VA)
- Other occlusion site, please specify [free text]
- Additional vessel occlusion (y/n)
- If yes, location of additional vessel occlusion
- In case of intracranial large vessel occlusion in the anterior circulation: collaterals on baseline angiography if assessable (TAN Score 0-3)
- Relevant stenosis (>50% NASCET)/occlusion of extracranial ICA on baseline angiography (Y/N)
- Relevant stenosis (>50% NASCET)/occlusion of extracranial VA on baseline angiography (Y/N)

- Type of baseline perfusion modality (none, CTP, MRP)
- Mismatch ratio according to local modalities (Y/N)
- Total perfusion lesion/infarct core mismatch ratio visually on baseline perfusion image (f.i. 1.2 = perfusion lesion is 20% larger than core) (number)DSA (Y/N)
- In case of intracranial large vessel occlusion in the anterior circulation: collaterals on DSA (ASITN/SIR grading)
- Complete recanalization on DSA (mTICI=2b/3) (Y/N)
- Date of recanalization on DSA
- Time of recanalization on DSA

Follow up

- Type of first follow-up native image (none, NCCT, MRI)
- Date of follow-up image
- Time of follow-up image
- Type of first follow-up vessel imaging (none, CTA, MRA-TOF, MRA-CE, ultrasound)
- Date of follow-up vessel imaging
- Time of follow-up vessel imaging
- Complete recanalization on follow-up vessel imaging (CTA/MRA/US) (Y/N)
- mTICI
- Any intracerebral hemorrhage (ICH)
- Fatal ICH
- Symptomatic ICH (ECASS-2 criteria) (Y/N)

Nordanstig A, *et al. BMJ Open* 2022; 11:e042211. doi: 10.1136/bmjopen-2020-042211

- Other ICH (SAH, SDH, etc.) [free text]
- NIHSS after 24h
- mRS after 3 months
- Recurrent ischemic stroke or TIA within 3 months (no, TIA(s), stroke(s) (if TIA and stroke, count stroke)
- mRS after 1 year

Other

- Modified TOAST (LAA, CE (including PFO), SAO, other, more than one, undetermined, stroke mimic
- Rare specific stroke causes to be studied in more detail (cervical artery dissection, intracranial artery dissection, endocarditis, vasculitis, coagulopathies)

Nordanstig A, *et al. BMJ Open* 2022; 11:e042211. doi: 10.1136/bmjopen-2020-042211
