# Supplementary material for: EndoVAscular treatment and ThRombolysis for Ischemic Stroke Patients (EVA-TRISP) registry: basis and methodology of a pan-European prospective ischaemic stroke revascularisation treatment registry
Source: BMJ Open. 2021 Aug 8;11(8):e042211. doi: 10.1136/bmjopen-2020-042211 (PMC8354282; doi:10.1136/bmjopen-2020-042211)
Supplement: Supplementary data [file bmjopen-2020-042211supp002.pdf]

Appendix 3: Names of the ethics committees

City	Ethics committees
Amsterdam	Anonymized registry based research does not need ethical approval in The Netherlands
Basel	The ethics committee Basel (Ethikkommission Nordwest- und Zentralschweiz (EKNZ))
Belgrade	The ethics committee of Clinical Centre of Serbia
Berlin	The ethical review committee of the Charité–University Medicine Berlin
Bern	The cantonal ethics Committee Bern
Bremen	The local ethic committee of the Ärztekammer Bremen
Bologna	Local regulations do not require approval by the ethics board for observational studies using registry data in IRCCS (Institute for Treatment and Research)
Brescia	EC ASST spedali civili university hospital Brescia
Goettingen	The ethics committee in Goettingen approved it (No.: 16/2/16).
Gothenburg	The regional ethical board of Gothenburg
Heidelberg	Ethik kommission der Medizinischen Fakultät Heidelberg
Helsinki	Local regulations do not require approval by the ethics board for retrospective studies using registry data
Larissa	Participation in EVA-TRISP does not require ethics approval in Greece
Jerusalem	Hadassah Medical Organization (HMO) Jerusalem
Lausanne	The ethics commission for research on humans of the Canton of Vaud has approved the scientific use data from the ASTRAL registry
Lille	Comité de Protection des Personnes Nord Ouest IV Lille, France
Modena	The study was approved by local EC called “Comitato Etico Area Vasta Emilia Nord"
Munich	The ethics committee of the chamber of physicians at Ludwig-Maximilians University LMU Munich
St. Gallen	Each study project has been approved by the ethical committee
Zurich	The ethics commission Zurich, Switzerland.
