# Supplementary material for: EndoVAscular treatment and ThRombolysis for Ischemic Stroke Patients (EVA-TRISP) registry: basis and methodology of a pan-European prospective ischaemic stroke revascularisation treatment registry
Source: BMJ Open. 2021 Aug 8;11(8):e042211. doi: 10.1136/bmjopen-2020-042211 (PMC8354282; doi:10.1136/bmjopen-2020-042211)
Supplement: Author's manuscript [file bmjopen-2020-042211.draft_revisions.pdf]

BMJ Open is committed to open peer review. As part of this commitment we make the peer review history of every article we publish publicly available.

When an article is published we post the peer reviewers' comments and the authors' responses online. We also post the versions of the paper that were used during peer review. These are the versions that the peer review comments apply to.

The versions of the paper that follow are the versions that were submitted during the peer review process. They are not the versions of record or the final published versions. They should not be cited or distributed as the published version of this manuscript.

BMJ Open is an open access journal and the full, final, typeset and author-corrected version of record of the manuscript is available on our site with no access controls, subscription charges or pay-per-view fees (http://bmjopen.bmj.com).

If you have any questions on BMJ Open's open peer review process please email info.bmjopen@bmj.com

BMJ Open

Cohort profile: EndoVAscular treatment and ThRombolysis for Ischemic Stroke Patients (EVA-TRISP) Registry: Basis and methodology of a pan-European prospective ischemic stroke revascularization treatment registry

Journal:	*BMJ Open*
Manuscript ID	bmjopen-2020-042211
Article Type:	Cohort profile
Date Submitted by the Author:	29-Jun-2020

Complete List of Authors:	Nordanstig, Annika; Sahlgrenska Academy, Department of Clinical Neurosciences Sahlgrenska Academy at University of Gothenburg; Department of Neurology, Sahlgrenska University Hospital<br>Curtze, Sami; Helsingfors universitets centralsjukhus, Department of Neurology, University of Helsinki and Helsinki University Hospital<br>Gensicke, H; University of Basel, Department of Neurology and Stroke Center, University of Basel and University Hospital Basel, Switzerland<br>Zinkstok, Sanne; Amsterdam University Medical Centres, Department of Neurology, Amsterdam University Medical Centers, the Netherlands<br>Erdur, Herbun; Charité Universitätsmedizin Berlin, Department of Neurology and Center for Stroke Research, Charité-Universitätsmedizin Berlin, Germany<br>Karlsson, Camilla; Sahlgrenska Academy, Department of Clinical Neurosciences, Sahlgrenska Academy at University of Gothenburg<br>Karlsson, Jan-Erik; Sahlgrenska Academy, Department of Clinical Neurosciences, Sahlgrenska Academy at University of Gothenburg; Department of Neurology, Sahlgrenska University Hospital<br>Martinez-Majander , Nicolas; Helsinki University Hospital, Department of Neurology, University of Helsinki and Helsinki University Hospital<br>Sibolt, G; HYKS sairaanhoitopiiri, Department of Neurology, University of Helsinki and Helsinki University Hospital<br>Lyrer, Philippe; University Hospital Basel, Department of Neurology and Stroke Center, University of Basel and University Hospital Basel<br>Traenka, Christopher; Universitätsspital Basel, Department of Neurology and Stroke Center, University of Basel and University Hospital Basel<br>Baharoglu, Merih; Amsterdam University Medical Centres, Department of Neurology, Amsterdam University Medical Centers<br>Scheitz, Jan; Charite Universitatsmedizin Berlin Klinik fur Neurologie mit Experimenteller Neurologie, Department of Neurology and Center for Stroke Research, Charité-Universitätsmedizin Berlin<br>Bricout, Nicolas; University of Lille, Department of Neurology, University of Lille<br>Hénon, Hilde; University of Lille, Department of Neurology, University of Lille<br>Leys, Didier; University of Lille, Department of Neurology, University of Lille<br>Eskandari, Ashraf; University of Lausanne, Department of Neurology, Centre Hospitalier Universitaire Vaudois and University of Lausanne

Michel, Patrik; University of Lausanne, Department of Neurology, Centre Hospitalier Universitaire Vaudois and University of Lausanne
Hametner, Christian; University Hospital Heidelberg, Department of Neurology, University Hospital Heidelberg
Ringleb, Peter; University Hospital Heidelberg, Department of Neurology, University Hospital Heidelberg
Arnold, Marcel; Inselspital University Hospital Berne Department of Neurology, Inselspital, Bern University Hospital, University of Bern
Fischer, Urs; Inselspital University Hospital Bern, Department of Neurology, Inselspital, Bern University Hospital, University of Bern
Sarikaya, Hakan; Inselspital University Hospital Bern, Department of Neurology, Inselspital, Bern University Hospital, University of Bern
Seiffge, David ; Inselspital University Hospital Bern, Department of Neurology, Inselspital, Bern University Hospital, University of Bern
Pezzini, Alessandro ; Università degli Studi di Brescia, Department of Clinical and Experimental Sciences, Neurology Clinic, University of Brescia,
Zini, Andrea; IRCCS Istituto Delle Scienze Neurologiche di Bologna, Department of Neurology and Stroke Center, Maggiore Hospital, Bologna
Padjen, Visnja; Clinical Center of Serbia, Neurology Clinic, Clinical Centre of Serbia, Belgrade
Jovanovic, Dejana R.; Clinical Center of Serbia, Neurology Clinic, Clinical Centre of Serbia, Belgrade; Faculty of Medicine, University of Belgrade
Luft, Andreas; University of Zurich, Department of Neurology, University of Zürich
Wegener, Susanne; University of Zurich, Department of Neurology, University of Zürich
Kellert, Lars; Ludwig-Maximilians-Universitat Munchen, Neurology
Feil, Katharina; Ludwig-Maximilians-Universitat Munchen, Neurology and German Center for Vertigo and Balance Disorders
Kaegi, Georg; Kantonsspital St Gallen, Department of Neurology, Kantonsspital St. Gallen
Rentzos, Alexandros ; Sahlgrenska Academy, Department of Radiology, Institute of clinical sciences, Sahlgrenska Academy, University of Gothenburg; Diagnostic and Interventional Neuroradiology, Sahlgrenska University Hospital and
Lappalainen, Kimmo; Helsinki University Central Hospital, Department of Radiology, Helsinki University Hospital
Leker , RR; Hebrew University Hadassah Medical School, Department of Neurology, Hadassah-Hebrew University Medical Center
Cohen, Jose; Hebrew University Hadassah Medical School, Department of Neurosurgery, Hadassah-Hebrew University Medical Center
Gomori, John; Hebrew University Hadassah Medical School, Department of Radiology, Hadassah-Hebrew University Medical Center
Brehm, Alex; Georg-August-Universitat Gottingen Universitatsmedizin, Department of Neurology, University Medical Center Goettingen
Liman, Jan; Georg-August-Universitat Gottingen Universitatsmedizin, Department of Neurology, University Medical Center Goettingen
Psychogios, Marios; Georg-August-Universitat Gottingen Universitatsmedizin, Department of Neuroradiology, University Medical Center Goettingen; Neuroradiology and Stroke Center Basel, University of Basel and University Hospital Basel
Kastrup, Andreas; University of Bremen, Department of Neurology, University Hospitals Bremen-Mitte and Bremen-Ost
Papanagiotou, Panagiotis; University of Bremen, Clinic for Diagnostic and Interventional Neuroradiology, University Hospitals Bremen-Mitte and Bremen-Ost; Dept. of Radiology, Areteion University Hospital, National and Kapodistrian University of Athens
Gralla, Jan; University of Bern, Institute of Diagnostic and Interventional Neuroradiology, lnselspital, Bern University Hospital, University of Bern
Magoni, Mauro; ASST Spedali Civili di Brescia, USD Stroke Unit and

	Vascular Neurology, ASST Spedali Civili Brescia, Brescia, Italy<br>Majoie, Charles; Amsterdam UMC - Locatie AMC, Department of Neuroradiology, Amsterdam University Medical Centers<br>Bohner, Georg; Charité Universitätsmedizin Berlin, Institute of Neuroradiology, Charité-Universitätsmedizin Berlin, Germany<br>Vukasinovic, Ivan; Clinical Center of Serbia, Clinic of Neurosurgery, Clinical Centre of Serbia, Belgrade<br>Cvetic, Vladimir; University of Belgrade, Faculty of Medicine, University of Belgrade; Clinic for Vascular and Endovascular Surgery, Clinical Centre of Serbia, Belgrade<br>Weber, Johannes; Kantonsspital St Gallen, Department of Radiology and Nuclear Medicine, Kantonsspital St. Gallen<br>Kulcsar, Zsolt; University Hospital Zurich, Department of Neuroradiology<br>Bendszus, Martin; UniversitatsKlinikum Heidelberg, Department of Neuroradiology<br>Möhlenbruch, Markus ; UniversitatsKlinikum Heidelberg, Department of Neuroradiology<br>Ntaios, George; University of Thessaly, Department of Internal Medicine, Faculty of Medicine, School of Health Sciences<br>Kapsalaki, Eftychia; University of Thessaly, Department of Radiology, University Hospital of Larissa, School of Medicine<br>Jood, Katarina; Sahlgrenska Academy, Department for Clinical Neuroscience and Rehabilitation; Department of Neurology, Sahlgrenska University Hospital<br>Nolte, Christian ; Charité Universitätsmedizin Berlin, Department of Neurology and Center for Stroke Research<br>Nederkoorn, Paul J; Amsterdam University Medical Centres, Department of Neurology<br>Engelter, Stefan; University of Basel, Department of Neurology and Stroke Center; Deptartment of Radiology, Areteion University Hospital, National and Kapodistrian University of Athens<br>Strbian, Daniel; University of Helsinki, Department of Neurology, University of Helsinki and Helsinki University Hospital<br>Tatlisumak, T; Sahlgrenska University Hospital, Department of Clinical Neurosciences; University of Helsinki, Department of Neurology
Keywords:	Stroke < NEUROLOGY, Stroke medicine < INTERNAL MEDICINE, Neurology < INTERNAL MEDICINE

SCHOLARONE™
Manuscripts

I, the Submitting Author has the right to grant and does grant on behalf of all authors of the Work (as defined in the below author licence), an exclusive licence and/or a non-exclusive licence for contributions from authors who are: i) UK Crown employees; ii) where BMJ has agreed a CC-BY licence shall apply, and/or iii) in accordance with the terms applicable for US Federal Government officers or employees acting as part of their official duties; on a worldwide, perpetual, irrevocable, royalty-free basis to BMJ Publishing Group Ltd ("BMJ") its licensees and where the relevant Journal is co-owned by BMJ to the co-owners of the Journal, to publish the Work in this journal and any other BMJ products and to exploit all rights, as set out in our licence.

The Submitting Author accepts and understands that any supply made under these terms is made by BMJ to the Submitting Author unless you are acting as an employee on behalf of your employer or a postgraduate student of an affiliated institution which is paying any applicable article publishing charge ("APC") for Open Access articles. Where the Submitting Author wishes to make the Work available on an Open Access basis (and intends to pay the relevant APC), the terms of reuse of such Open Access shall be governed by a Creative Commons licence – details of these licences and which Creative Commons licence will apply to this Work are set out in our licence referred to above.

Other than as permitted in any relevant BMJ Author's Self Archiving Policies, I confirm this Work has not been accepted for publication elsewhere, is not being considered for publication elsewhere and does not duplicate material already published. I confirm all authors consent to publication of this Work and authorise the granting of this licence.

For peer review only

Cohort profile: EndoVAscular treatment and ThRombolysis for Ischemic Stroke Patients (EVA-TRISP) Registry: Basis and methodology of a pan-European prospective ischemic stroke revascularization treatment registry

Annika Nordanstig[1*], Sami Curtze[2*], Henrik Gensicke[3*], Sanne M Zinkstok[4*], Hebun Erdur[5*], Camilla Karlsson[1*], Jan-Erik Karlsson[1], Nicolas Martinez-Majander[2], Gerli Sibolt[2], Philippe A Lyrer[3], Christopher Traenka[3], M Irem Baharoglu[4], Jan F Scheitz[5], Nicolas Bricout[6], Hilde Hénon[6], Didier Leys[6], Ashraf Eskandari[7], Patrik Michel[7], Christian Hametner[8],Peter A Ringleb[8], Marcel Arnold[9], Urs Fischer[9], Hakan Sarikaya[9], David J Seiffge[9], Alessandro Pezzini[10], Andrea Zini[11], Visnja Padjen[12], Dejana R Jovanovic[12,13], Andreas R Luft[14], Susanne Wegener[14], Lars Kellert[15], Katharina Feil[15], Georg Kägi[16], Alexandros Rentzos[17], Kimmo Lappalainen[18], Ronen R Leker[19], Jose Cohen[20], John Gomori[21], Alex Brehm[22], Jan Liman[22], Marios Psychogios[23], Andreas Kastrup[24], Panagiotis Papanagiotou[25,26], Jan Gralla[27], Mauro Magoni[28], Charles Majoie[29], Georg Bohner[30], Ivan Vukasinovic[31], Vladimir Cvetic[13,32], Johannes Weber[33], Zsolt Kulcsar[34], Martin Bendszus[35], Markus Möhlenbruch[35], George Ntaios[36], Eftychia Kapsalaki[37], Katarina Jood[1#], Christian H Nolte[5#], Paul J Nederkoorn[4#], Stefan T Engelter[3,26#], Daniel Strbian[2#], Turgut Tatlisumak[1,2#], on behalf of EVA-TRISP Investigators

*= shared first authorship; #= shared last authorship

[1]Department of Neurology, Sahlgrenska University Hospital and Department of Clinical Neurosciences, Institute of Neurosciences and Physiology, Sahlgrenska Academy at University of Gothenburg, Gothenburg, Sweden

[2]Department of Neurology, University of Helsinki and Helsinki University Hospital, Finland

[3]Department of Neurology and Stroke Center, University of Basel and University Hospital Basel, Switzerland

[4]Department of Neurology, Amsterdam University Medical Centers, the Netherlands

[5]Department of Neurology and Center for Stroke Research, Charité-Universitätsmedizin Berlin, Germany

[6]Department of Neurology, University of Lille, France

[7]Department of Neurology, Centre Hospitalier Universitaire Vaudois and University of Lausanne, Switzerland

[8]Department of Neurology, University Hospital Heidelberg, Germany

[9]Department of Neurology, lnselspital, Bern University Hospital, University of Bern, Switzerland

[10]Department of Clinical and Experimental Sciences, Neurology Clinic, University of Brescia, Italy

For peer review only - http://bmjopen.bmj.com/site/about/guidelines.xhtml

[11]IRCCS Istituto di Scienze Neurologiche di Bologna, Department of Neurology and Stroke Center, Maggiore Hospital, Bologna, Italy

[12]Neurology Clinic, Clinical Centre of Serbia, Belgrade, Serbia

[13]Faculty of Medicine, University of Belgrade, Serbia

[14]Department of Neurology, University of Zürich, Switzerland

[15]Department of Neurology, Klinikum der Universität München, Ludwig-Maximilians University, Germany

[16]Department of Neurology, Kantonsspital St. Gallen, Switzerland

[17]Diagnostic and Interventional Neuroradiology, Sahlgrenska University Hospital and Department of Radiology, Institute of clinical sciences, Sahlgrenska Academy, University of Gothenburg, Sweden

[18]Department of Radiology, Helsinki University Hospital, Finland

[19]Department of Neurology, Hadassah-Hebrew University Medical Center, Jerusalem, Israel

[20]Department of Neurosurgery, Hadassah-Hebrew University Medical Center, Jerusalem, Israel

[21]Department of Radiology, Hadassah-Hebrew University Medical Center. Jerusalem, Israel

[22]Department of Neurology, University Medical Center Goettingen, Germany

[23]Department of Neuroradiology, University Medical Center Goettingen, Germany, and Neuroradiology and Stroke Center Basel, University of Basel and University Hospital Basel, Switzerland

[24]Department of Neurology, University Hospitals Bremen-Mitte and Bremen-Ost, Germany

[25]Clinic for Diagnostic and Interventional Neuroradiology, University Hospitals Bremen-Mitte and Bremen-Ost, Germany

[26]Deptartment of Radiology, Areteion University Hospital, National and Kapodistrian University of Athens, Greece

[27]Institute of Diagnostic and Interventional Neuroradiology, lnselspital, Bern University Hospital, University of Bern, Switzerland

[28]USD Stroke Unit and Vascular Neurology, ASST Spedali Civili Brescia, Brescia, Italy

[29]Department of Neuroradiology, Amsterdam University Medical Centers, the Netherlands

For peer review only

For peer review only - http://bmjopen.bmj.com/site/about/guidelines.xhtml

[30]Institute of Neuroradiology, Charité-Universitätsmedizin Berlin, Germany

[31]Clinic of Neurosurgery, Clinical Centre of Serbia, Belgrade, Serbia

[32]Clinic for Vascular and Endovascular Surgery, Clinical Centre of Serbia, Belgrade, Serbia

[33]Department of Radiology and Nuclear Medicine, Kantonsspital St. Gallen, Switzerland

[34]Department of Neuroradiology, University Hospital Zürich, Switzerland

[35]University of Heidelberg, Neurology Clinic, Department of Neuroradiology, Germany

[36]Department of Internal Medicine, Faculty of Medicine, School of Health Sciences, University of Thessaly, Larissa, Greece

[37] Department of Radiology, University Hospital of Larissa, School of Medicine, University of Thessaly, Larissa, Greece

Corresponding author: Annika Nordanstig, MD, PhD, Department of Neurology, Sahlgrenska University Hospital, Gröna Stråket 11, 3rd floor, 41345 Gothenburg, Sweden
E-mail: annika.nordanstig@vgregion.se ; Tel: + 46 31 3429061, Fax: + 46 31 823650

Authors' e-mail addresses: annika.nordanstig@vgregion.se; sami.curtze@hus.fi; henrik.gensicke@usb.ch; s.m.zinkstok@amc.uva.nl; hebun.erdur@charite.de; camilla.l.karlsson@vgregion.se; nicolas.martinez-majander@hus.fi; jan.scheitz@charite.de; nicolas.bricout2@chru-lille.fr; didier.leys@univ-lille2.fr; ashraf.eskandari@chuv.ch; christian.hametner@med.uni-heidelberg.de; hakan.sarikaya@insel.ch; urs.fischer@insel.ch; mago1959@hotmail.com; a.zini@ausl.bologna.it; visnja.padjen@hotmail.com; dejana.r.jovanovic@gmail.com; andreas.luft@usz.ch; susanne.wegener@usz.ch; lars.kellert@med.uni-muenchen.de; katharina.feil@med.uni-muenchen.de; georg.kaegi@kssg.ch; christopher.traenka@usb.ch; m.i.baharoglu@amc.uva.nl; jan-erik.karlsson@vgregion.se; gerli.sibolt@hus.fi; alexandros.rentzos@vgregion.se; kimmo.lappalainen@hus.fi; david.seiffge@insel.ch; leker@hadassah.org.il; jcohenns@yahoo.com; gomori@cc.huji.ac.il; alex.brehm@med.uni-goettingen.de; jliman@gwdg.de; marios.psychogios@usb.ch; akastrup06@gmail.com; papanagiotou@me.com; alessandro.pezzini@unibs.it; marcel.arnold@insel.ch; peter.arthur.ringleb@med.uni-heidelberg.de; patrik.michel@chuv.ch; hilde.henon@chru-lille.fr; philippe.lyrer@usb.ch; christian.nolte@charite.de; p.j.nederkoorn@amsterdamumc.nl; jan.gralla@insel.ch; c.b.majoie@amsterdamumc.nl; georg.bohner@charite.de; vukasinovic_i@yahoo.co.uk; drvladimircvetic@gmail.com; johannes.weber@kssg.ch; Zsolt.Kulcsar@usz.ch; markus.moehlenbruch@med.uni-heidelberg.de; martin.bendszus@med.uni-heidelberg.de; ntaiosgeorge@gmail.com; gntaios@med.uth.gr; ekapsal@med.uth.gr; katarina.jood@neuro.gu.se; stefan.engelter@usb.ch; daniel.strbian@hus.fi; turgut.tatlisumak@neuro.gu.se

For peer review only - http://bmjopen.bmj.com/site/about/guidelines.xhtml

Running title: EVA-TRISP Registry Methodology
Number of words in manuscript body: 6399
Number of words in abstract: 298
Number of tables: 2
Number of figures: 1 (EVA-TRISP centers)
Number of references: 57
Number of appendices: 2
Appendix 1: EVA-TRISP Investigators (in alphabetical order by country)
Appendix 2: EVA-TRISP database items

For peer review only

For peer review only - http://bmjopen.bmj.com/site/about/guidelines.xhtml

ABSTRACT

Purpose

The Thrombolysis in Ischemic Stroke Patients(TRISP) collaboration was a concerted effort initiated 2010 with the purpose to address relevant research questions about the effectiveness and safety of intravenous thrombolysis(IVT). The collaboration also aims to prospectively collect data on patients undergoing endovascular treatment(EVT) and hence the name of the collaboration change from TRISP to EVA-TRISP. The methodology of the former TRISP registry for patients treated with IVT has already been published. This paper focuses on describing the EVT part of the registry.

Participants

All centers committed to collecting predefined variables on consecutive patients prospectively. We aim for accuracy and completeness of the data, and adapting local databases to investigate novel research questions.

Herein, we introduce the methodology of a recently constructed academic investigator-initiated open collaboration EVT registry built as an extension of existing IVT registry in patients with acute ischemic stroke(AIS).

Findings to date

Currently, the EVA-TRISP network includes 20 stroke centers with considerable expertise in EVT and maintenance of high-quality hospital-based registries.

Following several successful randomized controlled trials(RCTs), many important clinical questions remain unanswered in the(EVT) field and some of them will unlikely be investigated in

For peer review only - http://bmjopen.bmj.com/site/about/guidelines.xhtml

For peer review only

future RCTs. Prospective registries with high-quality data on EVT-treated patients may help answering some of these unanswered issues, especially on safety and efficacy of EVT in specific patient subgroups.

Future plans

This collaborative effort aims at addressing clinically important questions on safety and efficacy of EVT in conditions not covered by RCTs. The TRISP registry generated substantial novel data supporting stroke physicians in their daily decision-making considering IVT candidate patients. While providing observational data on EVT in daily clinical practice, our future findings may likewise be hypothesis-generating for future research as well as for quality improvement(on EVT). The collaboration welcomes participation of further centers fulfilling the commitment and the outlined requirements.

Key words

acute ischemic stroke, benchmarking, collaboration, endovascular treatment, large-artery occlusion, outcome, quality, recanalization, registry, stroke, thrombectomy, thrombolysis

For peer review only

For peer review only - http://bmjopen.bmj.com/site/about/guidelines.xhtml

Strengths and limitations of this study

- The EVA-TRISP collaboration offers a platform to pool individual patient data from prospective registries of patients with ischemic stroke undergoing revascularization therapies.
- The large sample size (currently >13 000 EVTs from 20 centres), high completeness of data and standardized data ascertainment are strengths of EVA-TRISP.
- EVA-TRISP will provide data from everyday clinical practice and address clinically important questions about safety and outcomes of patients with ischemic stroke treated with EVT who are neither covered by randomized controlled trials.
- Data is derived from registries that are neither monitored nor randomized. There will be no control group without EVT which disallows the assessment of effectiveness of EVT in study populations.

INTRODUCTION

Timely recanalization improves outcomes in patients with AIS [1, 2]. Safety and efficacy of recanalization strategies, namely IVT and more recently EVT (including mechanical thrombectomy with various techniques and devices in AIS patients with anterior circulation large-artery occlusions), have been well-documented in several randomized controlled trials (RCTs)[3-9]. A meta-analysis of five RCTs revealed an average 2.5-fold reduction in disability through EVT in large-vessel occlusions compared with standard care, including IVT[10]. Early

For peer review only - http://bmjopen.bmj.com/site/about/guidelines.xhtml
For peer review only

recanalization is currently the cornerstone of acute stroke treatment with increasing use globally. This benefit is substantially higher with earlier achievement of recanalization and diminishes with longer onset-to-treatment intervals[2, 11, 12]. Previous research has explicitly shown that IVT with alteplase within 4.5 hours of symptom onset improved AIS patient outcomes[13].

Following the results of these RCTs, EVT is recommended as standard of care in patients with intracranial large vessel occlusion in several guidelines[14-16]. Consequently, health systems all over the world adapted themselves to identify and quickly transfer eligible patients to centers offering EVT. Simultaneously, capacity, logistics, know-how and 24/7 coverage were developed to cope with the quickly increasing demand for this intervention. Moreover, two recent RCTs showed benefit with EVT in patients treated up to 16 or 24 hours after stroke onset given that presence of a considerable amount of salvageable brain tissue had to be demonstrated with appropriate imaging methods [17, 18].

Relevant questions in the daily clinical work of treating stroke patients suffering from large vessel occlusions remain. First, EVT-RCTs included a highly selective patient population increasing chances to demonstrate efficacy and to exclude patients who presumably have low chances for a favorable outcome and patients who have high risk for serious complications. Secondly, the seven published EVT trials[3, 5-9, 19] analyzed altogether only included 1754 randomized patients (of whom 869 underwent EVT) with the single smallest trial including only 65 patients (of whom 33 underwent EVT)[8]. Usually, after a novel treatment is proved effective, a new wave of assumptions and extrapolations for treating a broader domain of patients begins. As all these excluded patient subgroups cannot be studied in future RCTs, in most cases judgments for treating or not treating with EVT will be based on limited knowledge. Some remaining questions will eventually be answered with a long delay, but some will never be answered in forthcoming RCTs. However, stroke physicians keep facing patients where evidence-based data

For peer review only - http://bmjopen.bmj.com/site/about/guidelines.xhtml

do not explicitly contribute to decision-making for these individuals, in which available treatments may very well have a potential benefit as well. Here, prospective high-quality multicenter registries including large numbers of patients representing many subgroups not included or not separately analyzed within RCT settings may offer helpful information for basing clinical judgments while being aware that the level of certainty will not reach that gained from RCTs. These registries may also give strong clues on how trial results are implemented to clinical practice and how daily practice safety and efficacy levels match with those gained in RCTs. Further, registry-based data deliver hints in generating new and adequate hypotheses for future RCTs. Another important aspect is the recently developing new field of clot property-research: interested centers can collect detached clots and ship to laboratories where macroscopic and microscopic properties of the clot coupled with clinical data can be further investigated and may open new avenues in understanding stroke mechanisms. Lastly, quality is a central indicator in health care and registry-based data can be utilized in comparisons and for improving individual center acute stroke care pathways. As a prerequisite, such data have to be based on well-maintained registries containing a large number of detailed, clearly-defined, and well-characterized variables. EVA-TRISP registry aims at meeting these prerequisites. Utilizing our decade-long experience from the multinational TRISP registry[20], we are now aiming to build a prospective multinational registry of AIS patients treated with EVT including detailed clinical, laboratory, and imaging data for future analyses. We are presenting herein the current versions of the clinical and imaging database items of the EVA-TRISP registry. Additionally, we will discuss a selection of specific related topics.

COHORT DESCRIPTION

For peer review only - http://bmjopen.bmj.com/site/about/guidelines.xhtml

The Thrombolysis in Ischemic Stroke Patients (TRISP) collaboration was a concerted effort initiated 2010 by 11 European stroke centers with the purpose to address clinically relevant research questions about the effectiveness and safety of IVT, and currently 20 stroke centers from nine different countries participate in the collaboration (see Figure 1 and Appendix 1 for a list of member sites and investigators). As the collaboration also aims to prospectively collect granular and high-quality data on all consecutive stroke patients undergoing EVT, the name changes from TRISP to EVA-TRISP.

EVA-TRISP (former TRISP) operates as an independent, non-profit, investigator-driven open platform that focuses on generating high quality data for clinical research purposes. The EVA-TRISP research initiatives are characterized by informal, project-driven and relaxed work processes based on a high level of mutual trust and understanding between participating collaborators, and no formal scientific leadership committees have so far been implemented or deemed necessary. Internal communication principally takes place via e-mail and teleconferences. The EVA-TRISP collaboration also hosts an annual face-to-face meeting during the European Stroke Organisation (ESO) conference, and this forum is utilized for overall strategic planning and major decisions. Brief meeting minutes from the teleconferences and the annual face-to-face meeting are disseminated to all participating centers. The collaboration welcomes new collaborators and project proposals from all stroke centers that fulfill the requirements as stated below. The collaboration particularly aims at supporting young researchers. Thus, with the exception of the very first paper[21], first authors of the publications generated by the TRISP invariably have been young stroke physicians or PhD-students. The methodology of the TRISP registry has previously been published[20], and currently, data on more

For peer review only - http://bmjopen.bmj.com/site/about/guidelines.xhtml

than 18,000 IVT-treated patients are available in the registry. This paper focuses on describing the EVT part of the registry.

The EVA-TRISP registry aims to prospectively collect granular and high-quality data on all consecutive AIS patients undergoing both IVT and/or EVT. The overall purpose is to provide a means to address clinically important research questions on the safety and effectiveness of IVT and/or EVT in AIS patients that are typically not covered by RCTs or single center research initiatives. Furthermore, the EVA-TRISP aims at providing a large data source for various stroke care quality improvement initiatives. All EVA-TRISP centers have a proven track-record for delivering high-quality and high-volume stroke patient care. Every EVA-TRISP center offers stroke management that fulfill the criteria of Stroke Centers or Stroke Units as proposed by the ESO[22]. The simple idea of the EVT part of the EVA-TRISP registry is that experienced stroke centers with expertise in both EVT implementation and in the maintenance of hospital-based EVT databases pool their data together. An advantage of the EVA-TRISP registry is the availability of substantially more variables than in other large-scale registries. We strive for a commitment by the collaborators to provide data of high accuracy and completeness as well as towards a willingness among collaborators to swiftly adapt the local databases by adding new variables of interest. This enables a potential for explorative insights in the putative prognostic importance of variables with unknown influence on outcome or risk of complications, such as symptomatic intracranial hemorrhage (sICH). Strengths and limitations of the EVA-TRISP registry in general and compared to other existing EVT registries are discussed below (please see Discussion).

The currently participating centers all have agreed to fulfill the prerequisites that are summarized in Table 1. Participation in other registries does not preclude participation in the EVA-TRISP registry. A standard database template has been developed by an international expert group

For peer review only - http://bmjopen.bmj.com/site/about/guidelines.xhtml

consisting of stroke physicians and scientists in collaboration with all members leading to the current standard version of the registry (see Appendix 2-Registry data elements) that have been agreed upon by all EVA-TRISP member sites. This comprehensive dataset includes over 110 items and covers a wide range of data elements, including demographics, pre-stroke health information, acute phase management, and long-term outcomes including three months and one-year modified Rankin Scale (mRS) as well as detailed laboratory data and imaging findings. An add-on imaging repository is currently under preparation and will be implemented within the near future. Electronic medical records used at all member sites allow for quickly and reliably collecting additional variables when deemed necessary for new projects.

Lead of a single project and authorship principles

The researcher or researchers - usually one or two (rarely three) - who originally presented the idea and rendered the analysis proposal make(s) the initiation by drafting a standard 1-2 pages draft stating a clear hypothesis and statistical plan summarizing the project (project proposal). The proposal is circulated to all member centers and discussed for scientific content and feasibility enriched with input from a large expert community. After that, the enriched plan along with the list of data items required is recirculated. If a center agrees to participate, the center contributes data within the in-advance agreed time frame on all consecutive patients with the key variables of interest. For some projects retrospective collection of data is required. The original proposal makers are entitled to the first and senior authorships. Co-authorships are distributed according to contributions. This includes not only mere quantitative means (i.e. number of patients contributed) but also quality of data (e.g. completeness; considered high across EVA-TRISP centers), handling and pooling of the multicenter data; maintenance of the pooled data set (including data cleaning), statistics, contribution to EVA-TRISP in general, and intellectual input

For peer review only - http://bmjopen.bmj.com/site/about/guidelines.xhtml

in details of the design or the analyses of the research project, and lastly intellectual input to the writing and improving of the manuscript. These criteria are suggestions and the researches taking the lead in each project take the final responsibility for the fair distribution of authorships.

Each member site possesses its own data and each member site whose data are utilized, is entitled to co-authorship(s). Whenever feasible, an abstract approved by all co-authors is submitted to the forthcoming ESO Conference. All EVA-TRISP member centers and investigators are listed at the end of the manuscript as a supplement given that the publishing journal's own format allows this approach.

Data collection and definitions

Data on the characteristics of patients treated with EVT are collected prospectively by all participating centers using standardized definitions and a standardized form. Not all centers have to provide data on all variables but have given a commitment to add missing variables retrospectively, if considered relevant to answer a specific research question.

The dataset includes patient demographics, history, prehospital information, admission data, details on acute interventions, stroke unit and/or intensive care information, discharge, rehabilitation, outcome data (three months and one-year outcomes measured by mRS), as well as detailed laboratory test results, vital signs, and imaging findings (see Appendix 2). Risk factors and stroke etiology will be determined according to standard approaches across centers[20].

Moreover, neuroimaging findings before and after treatment are systematically ascertained and comprise imaging modality (computed tomography versus magnetic resonance imaging) as well as specific imaging findings such as hyperdense artery sign, presence and extent of early ischemic signs, site of vessel occlusion, collateral status, presence of tandem occlusion of ipsilateral carotid artery, recanalization status immediately after EVT and on follow-up imaging

For peer review only - http://bmjopen.bmj.com/site/about/guidelines.xhtml

(quantified according modified treatment in cerebral ischemia (mTICI) score)[23], white matter disease severity, presence and burden of cerebral microbleeds.

All patients are monitored for occurrence of hemorrhagic transformation. Follow-up imaging usually take place close to 24 hours after treatment or earlier in case of clinical worsening. Some centers perform follow-up imaging only in case of clinical worsening. The definition of symptomatic intracerebral hemorrhage (sICH) is in accordance with the definition used in the European Cooperative Acute Stroke Study II ("an intracranial hemorrhage was defined as symptomatic if the patient had clinical deterioration causing an increase in the National Institute of Health Stroke Scale (NIHSS) score of more than or equal to four points and if the hemorrhage was likely to be the cause of the clinical deterioration")[24]. The majority of centers additionally evaluate type of hemorrhagic transformation (hemorrhagic infarction, parenchymal hemorrhage), indicate whether the bleeding occurred remotely from the infarcted area, and document sICH according to definitions used in the National Institute of Neurological Disorders and Stroke Trial I+II, SITS-MOST and ECASS III definitions[25, 26, 27]. Functional outcomes at three months and one-year are assessed using the mRS. The mRS is obtained by telephone calls, postal/electronical questionnaire, or outpatient visits. If patients cannot be interviewed, close relatives, nurses or family doctors are asked for disability status.

We are currently exploring technical, legal, and ethical as well as financial backgrounds for establishing an electronic registry to one of the member centers that would allow direct data insertion from each center and holding the registry compactly in one single file. Otherwise, each center will maintain own registry within their own electronic system and data will be transferred always without personal identifiers for each single analysis looking at one single aspect and leading to one mutual international publication. Establishing and maintaining such a large

For peer review only - http://bmjopen.bmj.com/site/about/guidelines.xhtml

database with complete compliance to rules and safety measures is a costly procedure and requires long-term funding. This option is now under exploration.

Future plans in the registry include the addition of a neuroradiology imaging bank that will enable detailed image analysis of patients treated with IVT and/or EVT. The imaging bank will provide "real-world" diagnostic neuroradiology and, used in combination with detailed clinical information from the EVA-TRISP registry, analysis of this imaging data will help to (i) create standardized imaging protocols in acute stroke (i.e. defining optimal threshold for perfusion parameters), (ii) identify new (i.e. a collateral score for the posterior circulation) and validate published (i.e. different collateral scores for the anterior circulation) imaging outcome predictors and imaging-based selection tools for reperfusion therapies, (iii) assess the generalizability of RCT results to subgroups of patients who would have been excluded based on imaging criteria (i.e. baseline Alberta Stroke Program Early CT score [ASPECTS] under five, extracranial vessel pathologies), (iv) improve automated analyzing techniques (i.e. machine and deep learning algorithms), (v) enhance the accuracy of outcome prediction of different clinical and imaging parameters by implementing new imaging outcomes (f.i. infarct volume, recanalization status). Neuroimages from TRISP/EVA-TRISP patients since 2015 will be pooled centrally. All imaging modalities (non-contrast CT, CT-angiography, CT-perfusion, MRI, MRA, MR-perfusion, and digital subtraction angiography) at baseline and follow-up (up to three months after stroke onset) will be eligible for analysis. Image analyses will be performed blinded to clinical information and treatment decisions, and undergo a central systematic re-evaluation using a specified case report form including all predefined imaging variables.

Stroke-specific image analyzing software (i.e. OSIRIX medical imaging viewer, Quantomo for semi-automated volumetric analysis, and Rapid Processing of Perfusion and Diffusion [RAPID]

For peer review only - http://bmjopen.bmj.com/site/about/guidelines.xhtml

for perfusion analysis) could be utilized. A detailed case report form for image analysis will be designed in review with all collaborators.

Lastly, EVA-TRISP investigators prepared a detailed standard operating procedure (SOP) to ascertain that all members collecting data are well-aware of standard interpretations and follow identical steps to avoid unnecessary heterogeneities or individual-borne differences. The numbers of recruited patients in each participating center during the study period until the end of year 2019 are reported in Table 2 along with the population each center is covering for EVT.

Data ownership, access, use, and publications

Each center is self-financing in data collection and is indisputable owner of own data. If and when a mutual single databank is constructed, each individual researcher with necessary formal training and permissions will be able to insert data directly to the central database on the internet and access to own center's data without limitations. Individual scientists working on a properly agreed single project and doing data analyses will be granted proper access to all data. In case that an individual center refrains from participating to a particular analysis, their data will not be included in that analysis. Any publication that is produced from the registry data will include authors from each contributing center in accordance to number of patients delivered as well as active involvement in analyses and writing work. Number of authors and their placement in the author list may vary regarding the amount of contributions. This will be handled openly and in a delicate way aiming at mutual consent.

Data elements and completeness:

A detailed database is aimed to allow investigating various current and future topics. Data elements are listed in detail in Appendix 2. Over time, new data elements may become necessary

For peer review only - http://bmjopen.bmj.com/site/about/guidelines.xhtml

for new individual projects. Should this occur, investigators will quickly supplement the missing variables. Patient age, sex, admission NIHSS score, recanalization status before and after thrombectomy, and three months outcome measured by mRS are obligatory data items and must be present for all patients (otherwise a patient is not eligible to be included to the registry). In general, missing data for any variable or patient must not exceed 10%.

Data sources

All paper-based or electronic patient files including laboratory values and imaging data will be utilized. When feasible, missing data will be completed by reconstructing e.g. NIHSS scores. In most cases, the local investigators form the local stroke team will be actively seeing the patients already at the emergency room and at their stroke units and can therefore guarantee completeness of data in most cases by collecting missing items directly from the patients and their relatives.

Target population

All AIS patients designated for EVT and in whom interventionalists gained arterial access are within the target population. Therefore, this registry also may include patients with misdiagnosis, already recanalized leading to premature interruption of the procedure, unsuccessful attempts to recanalize, and other unforeseeable conditions. Inclusion of patients is not limited to certain EVT techniques. The registry will include also patients who receive intra-arterial thrombolysis even without mechanical thrombectomy.

Registry size and duration

The registry will include all EVT patients from all member sites. We anticipate that the absolute numbers and proportions of EVT-treated AIS patients will be increasing over time and annual

For peer review only - http://bmjopen.bmj.com/site/about/guidelines.xhtml
For peer review only

inserts will exceed thousands shortly. The registry size is not limited. One anticipated strength of this registry is the high patient number together with detailed information on each patient allowing looking at many issues that are not feasible to investigate within RCTs or even merged data from all RCTs because of the fact that they included fairly small patient numbers. The use of the registry will be launched after 5000 patients' data have been inserted and adequately quality-checked. Similarly, this registry will be utilized as long as EVT is a viable option in stroke treatment. The unlimited time span requires careful evaluation by ethics committees. If the consortium decides to end the registry, each center's data will be adequately returned to owners and the registry data will be deleted achieving absolutely non-retrievable condition according to technical standard operating procedures (SOPs) of the registry-holding center. Thereafter, each individual center will be free to decide how to proceed with their own datasets. Similarly, if a center decides to resign from the registry, their data will be adequately returned and will be deleted from the main database file after confirmation that the data are safely received by the local principal investigator. While all patient-related data including clinical, laboratory, and imaging data are completely anonymized, each center will keep a key file within their local electronic hospital system with patient identifiers matching to the patient code on the registry (e.g. if necessary to go back to patient files). This approach is compliant with current principles and is the SOP worldwide.

Quality control

Quality control is another crucial step in multicenter large-sized registries since missing data are a frequent problem impairing the reliability and generalizability of registry-borne data. The EVA-TRISP registry is different in this sense because data are not yet directly collected to a central registry, but each center collects own data to their own institutional registry according to a

For peer review only - http://bmjopen.bmj.com/site/about/guidelines.xhtml

standard harmonized database item list and SOP. Later, the data are merged to a single file for maintenance and analyses. Therefore, missing data are expected to be close to nil. All centers will be including all consecutive patients attempted with an EVT and doing frequent checks not to leave any patient out of the registry. Our registry data will likely include all EVTs performed within a region and population practically equaling to a population-based study, although being hospital-based, because EVT is available only at stroke centers serving a predefined region and the inhabitant population in most cases. Most of the required data come from routine procedures which are standardly collected and recorded in stroke patient care pathways as part of the clinical routine and therefore almost always retrievable. The whole database will be checked for missing data as well as illogical entries. It is also feasible to set range limits to database cells to avoid or reject illogical entries (i.e. range and consistency checks; e.g. NIHSS score cannot be minus or over 42 points and can only be full points and not decimals; patient age at stroke onset can be only in digits, and is expected to be from 16 and very rarely over 100). There are other procedures regarding quality check, for example comparing retrospectively and prospectively entered data as well as comparing centers. These approaches will be run at certain milestone points.

Ethics, informed consent, and privacy

Each center has received necessary official approval from their respective local authorities and/or ethical committees according to their national and local rules. These permits include transfer of data between EVA-TRISP centers. Necessity of individual informed consent is dependent on national rules and will be collected if necessary. Data are shared with respect to the EU law 2016/679 about General Data Protection Regulation (GDPR). In the long run, a permanent

For peer review only - http://bmjopen.bmj.com/site/about/guidelines.xhtml

database residing at a member site is aimed. Establishment and maintenance of the permanent database at one center will be initiated only after a separate ethics approval.

Aims of the EVA-TRISP Registry

The major aim of EVA-TRISP is to address in AIS patients treated with IVT and/or EVT clinically important questions about safety and outcomes that are not covered by RCTs. The idea of EVA-TRISP is that experienced stroke centers with a record and expertise in both (I) usage of IVT and/or EVT and (II) maintenance of hospital-based stroke databases pool their data. In addition to the characteristics of the EVA-TRISP centers stated above, an advantage of EVA-TRISP is the availability of more additional variables than in other large-scale registries and the commitment by the collaborators to I) accuracy and completeness of the data and to II) the willingness to adapt the local databases and add quickly new variables retro- and prospectively.

DISCUSSION

Endovascular treatment, now fulfilling criteria for the highest level of evidence, has changed acute stroke care substantially. Probably approximately 10% of all ischemic stroke patients are eligible for EVT, but the percentages may grow as more and more patients are brought to the attention of emergency systems and the treatment indications will likely expand over time[28]. The rapid developments in acute stroke care put considerable demands on health care systems and necessitate quick rearrangements for coupling these needs. The published seven RCTs and following meta-analyses answered most central questions. Nevertheless, there are numerous

For peer review only - http://bmjopen.bmj.com/site/about/guidelines.xhtml

unanswered questions remaining in terms of EVT in acute ischemic stroke. Some of these questions will be solved and satisfied via ongoing and forthcoming RCTs. Yet, many other issues will never or unlikely be tested in RCTs and yet stroke physicians need firm data on these topics to base their clinical decisions on. Moreover, there are certain patient groups where RCTs are ethically difficult to organize; such a group is patients with basilar artery occlusion (BAO). Although a clearly important clinical condition that, untreated, have poor outcomes, BAO patients were not included in the large EVT trials. An extrapolation from the anterior circulation EVT trial results to BAO currently have strong support in clinical practice, and thus EVT are currently offered to BAO patients despite limited direct evidence of treatment effectiveness. A small multi-center RCT that included 131 patients– the Chinese BEST trial (Basilar artery occlusion Endovascular intervention versus Standard medical Treatment)[29]– was prematurely terminated due to slow recruitment and a high crossover rate that severely hampered the interpretability of the intent-to-treat analysis. Another RCT with 300 patients included is closed, but data has not yet been presented[30]. Still, both the per-protocol and the as-treated analyses favored EVT compared to best medical treatment. Some small-sized registry data showed high recanalization rates and similar hemorrhagic complication rates as in anterior circulation patients treated with EVT, but more often futile recanalization. Large-scale registry studies may further improve our knowledge in this patient group and may help identifying those who will likely benefit or not benefit from EVT in a real-life setting[6, 31-33]. In the absence of RCT-based data, comprehensive observational data may be useful for individual treatment decisions in clinical practice and in evaluating processes of stroke triage and care for IVT or EVT. As a prerequisite, such data have to be based on well-maintained registries containing large numbers of detailed, clearly-defined, and well-characterized variables. EVA-TRISP registry meets these prerequisites.

For peer review only - http://bmjopen.bmj.com/site/about/guidelines.xhtml

Ideally, the results from such observational studies are verified or falsified by RCTs. However, with few exceptions (e.g. age limit) this is unlikely to happen. Thus, registry-based data will reflect the highest level of evidence in several aspects, available currently and in the foreseeable future.

Further, we need to continuously follow-up whether the safety and benefit aspects of EVT shown in RCTs could be correctly translated to routine clinical practice. Indeed, the safety and benefit may be better, similar, or even worse in daily practice. Registry data can easily be compared to RCT data especially when basic settings are similar. Additionally, it becomes more and more feasible to compare centers, patient subgroups and devices. Benchmarking, previously performed by site visits, is a popular approach for understanding differences and making improvements, can now be done easily using electronic data[34].

Systematically ascertained, comprehensive and high-quality observational data are useful to both (I) challenge or (II) confirm the clinical usefulness of commonly used but often arbitrary eligibility criteria. An early example has been the challenge of the usefulness of the upper age limit of 80 years for IVT based on comprehensive, observational studies. Eventually, the third International Stroke Trial (IST-3) proved that indeed patients aged 80 years and older benefit from IVT, too[35].

Utilizing the TRISP registry, we examined previously safety of IVT in a number of patient subgroups where RCT-based data did not exist. Previous publications of TRISP registry (I) provided insight into safety and efficacy of IVT in subgroups of patients who were excluded in RCTs (e.g. patients dependent on the help of others prior to stroke), underrepresented or not specifically addressed (e.g. dissection as cause, impaired renal function, low platelet count, body-mass-index, prior use of statins, serotonin uptake inhibitors, prior use of novel oral anticoagulants, patients with seizure at onset)[21, 36-45], (II) were helpful to evaluate processes of

For peer review only - http://bmjopen.bmj.com/site/about/guidelines.xhtml

acute stroke care such as the meaning of the “off-hour-thrombolysis”, IVT during “working hours”, or the variable “time” in clinical practice[46-48], and (III) served to derive, validate, and compare risk scores for sICH or functional three months outcome[49-51]. These registry-based novel data contributed to the numbers of patients treated safely and successfully with IVT globally. Disease- or intervention-based patient registries with consecutive patients recruited in a population- or hospital-based approach are useful in many ways: they help describing the natural history, determine clinical effectiveness and cost-effectiveness of health care products or services, measure or monitor safety and harm, measure quality of care, improve quality of care, and help with benchmarking purposes such as how clinical practices vary, what the best predictors of treatment practices are, and comparing different practices providing a basis for further improvements. In such settings, stakeholders are several: the primary stakeholder with the EVA-TRISP registry is the academic consortium establishing and running the registry. Potential stakeholders with such a large-scale registry may include public health and regulatory authorities, product manufacturers, health care service providers, payer and commissioning authorities, patients and their advocacy groups, treating physician groups, academic institutions, and professional societies. The EVA-TRISP registry aims at including all patients who underwent EVT as a treatment for AIS, including patients with misdiagnosis, unsuccessful attempts (EVT is defined as a puncture to the artery with the aim of recanalization), or other unforeseeable scenarios. In most countries, registry-based studies are approved by ethics committees with waving informed consent from individual patients as demanding informed consent would leave most severe patients out of the registry and cause a severe bias on representability of any finding. Imaging has become more and more critical in stroke field. In addition to stroke diagnostics and excluding competing etiologies (e.g. stroke mimics), there are a number of imaging findings related to increased risk following acute treatments (e.g. leukoaraiosis[52] and microbleeds), or

For peer review only - http://bmjopen.bmj.com/site/about/guidelines.xhtml

findings guiding treatment choices (e.g. major artery occlusion reachable with a catheter), as well as findings helpful in prognostics such as ASPECTS[53] or SEDAN[49] scores. Acute stroke patients are increasingly imaged with a package of standard CT, CT angiography and CT perfusion or in a similar fashion with an MRI-based package. These imaging modalities are then analyzed quickly for determining diagnosis, prognosis, and treatment approach. Functional imaging modalities are increasingly taken into account for patient selection to IVT and EVT instead of strictly deciding according to time. The former TRISP registry included few items on imaging studies. Imaging requirements were according to routine thrombolysis: only a non-contrast CT imaging prior to thrombolysis was mandatory. Imaging was not in the main focus as image-analysis is usually labor-intense and not always available. Technological improvements may substitute some of the expert workforce in image analysis already today and in near future. However, with the developments in imaging technologies and increased requirements in patient care, now, most patients are imaged with CT angiography and CT-perfusion in addition to basic non-contract CT imaging. Less frequently, patients are imaged with a similar versatile package of various MRI sequences. Developments in the imaging technology and logistics, decrease in radiation dose used with CT imaging, as well as automatic image-analysis software development contributed to the progress. Installing imaging scanners into or adjacent to emergency departments or taking acute stroke suspect patient directly to the imaging facility by-passing emergency room have also improved availability of more detailed imaging. Patient selection for the best individual treatment is becoming increasingly dependent on neuroimaging with the goal of providing rapid patient-specific metrics such as tissue viability, vessel patency status, thrombus characteristics and cerebral perfusion etc. Imaging findings have also been used for patient selection in some highly successful IVT and EVT RCTs[4, 5, 54-56]. Detailed imaging information is even more crucial when EVT is considered. Therefore, establishing an imaging repository parallel with the EVA-TRISP

For peer review only - http://bmjopen.bmj.com/site/about/guidelines.xhtml

registry received a widespread support from members. To date, the choice of imaging modalities, parameters and thresholds varies widely across medical centers. No standardized imaging protocols currently exist, other than joint statements from professional societies[57]. A large, multicenter neuroimaging registry with state-of-the-art re-evaluation of images combined with detailed clinical data of IVT/EVT-treated stroke patients would be helpful for validating between modalities, defining thresholds, enhancing automated assessments and creating standards in neuroimaging for acute ischemic stroke. For the imaging part of the database we will collect baseline, interventional and follow-up images (up to three months after stroke onset) from all stroke patients included in TRISP since 2012. All images will be centrally analyzed using a predefined, standardized form.

Strengths and limitations of the EVA-TRISP registry

Strengths of EVA-TRISP registry include (I) the high completeness level of data with few missing data, (II) large sample sizes which reduce the risk of bias and allows adjustments for confounders, (III) the systematic and standardized data ascertainment which increases the homogeneity of the study population, (IV) the intrinsic motivation of the study personnel, leads to a high rate of completeness of ascertained data sets, contributing to a high-quality registry, and (V) the dynamic nature of the EVA-TRISP database due to the commitment of the centers to adapt the local database and add variables retro- and prospectively. In addition, (VI) a large number of variables is gathered including those with unknown prognostic importance. This allows addressing novel yet unidentified research questions. Moreover, (VII) pooling of individual patient data increases generalizability compared to single center studies, and (VIII) the fact, that variables and outcomes have been collected irrespective of the present research question, reduces the risk of a bias. (IX) As most EVA-TRISP centers are regional reference

For peer review only - http://bmjopen.bmj.com/site/about/guidelines.xhtml

centers for acute patient care, particularly for EVT, the EVA-TRISP registry will resemble a population-based registry. Limitations are inherent to the design of EVA-TRISP: (I) Data is derived from registries that are neither monitored nor randomized. Usually, there will be no control group without EVT which disallows the assessment of effectiveness of EVT in study populations. (II) As true for all observational studies, analyses based on registers have a higher risk of bias than RCTs. Thus, we urge to a cautious interpretation of findings and observations. (III) All EVA-TRISP centers are experienced in stroke treatment. This expertise implies – as a downside – a limited generalizability of findings to all stroke providers with less expertise and less advanced setting. (IV) The majority of our included patients are Caucasians and from high-income countries. Thus, we cannot compare ethnical differences, nor can compare health systems with various funding levels. (V) Currently, there is no 'core lab' to validate hemorrhagic complications and three-month mRS ratings. As valid for other major registries like SITS and GWTG, local interpretation of outcome data may differ between sites. Since EVA-TRISP centers are mostly high-volume centers with long-standing experience in maintaining IVT databases, this bias is likely to be smaller than in most of the other registries.

For peer review only

SUMMARY

The EVA-TRISP collaboration is an open platform dedicated to conduct joint research projects in AIS patients treated with IVT and/or EVT. EVA-TRISP aims to increase knowledge on safety and efficacy of IVT and EVT, study outcomes after IVT and EVT, to evaluate processes of acute stroke care as well as document and improve acute stroke care quality. Our previous achievements prove that this collaboration has the potential to provide versatile observational

For peer review only - http://bmjopen.bmj.com/site/about/guidelines.xhtml

information on treatment of AIS patients faced during daily clinical practice. Prospective and standardized documentation of individual patient data according to consensus definitions is a major requirement to maintain the quality of the EVA-TRISP registry. Publishing this methodology paper improves the transparency of the registry and collected data. EVA-TRISP welcomes participation and project proposals of further centers fulfilling the requirements stated above.

Consent for publication

Not applicable.

Availability of data and materials

Not applicable.

Competing interests

The authors declare that they have no competing interests.

Patient and public involvement

Patients or the public were not involved in the design, or conduct, or reporting, or dissemination plans of our research.

Funding

None.

Authors' contributions

For peer review only - http://bmjopen.bmj.com/site/about/guidelines.xhtml

Study concept and design: All authors. Data collection: All authors. Manuscript drafting: AN, SC, HG, SMZ, HE, CK, CHN, PJN, KJ, STE, DS, TT. Study supervision: TT, DS, STE, KJ, PJN, CHN. Statistical analysis and interpretation: do not apply. Review of the manuscript for intellectual contribution: All authors. All authors agreed on submitting this last version of the manuscript to the journal.

Acknowledgments

We thank Ms. Anu Eräkanto and Ms. Judith Klecki for technical support.

Disclosures

TT: Academic grants from the European Union, National Stroke Research Institute of Australia, Sahlgrenska University Hospital, Sigrid Juselius Foundation, University of Gothenburg, and Wennerström Foundation. Study contracts with Bayer, Boehringer Ingelheim, Bristol Myers Squibb, and Portola Pharm. Personal fees from Bayer, Boehringer Ingelheim, Bristol Myers Squibb, Lumosa Pharmaceuticals, and Portola Pharmaceuticals. TT received speaker's fees from the University of Donau, Krems, Austria. He holds patents on a treatment aiming to avoid post-thrombolytic brain edema and intracerebral hemorrhage in stroke (method to prevent brain edema and reperfusion injury; method to prevent post-thrombolytic hemorrhage formation).

STE has received funding for travel or speaker honoraria from Bayer, Boehringer Ingelheim and Daiichi-Sankyo. He has served on scientific advisory boards for Bayer, Boehringer Ingelheim, BMS/Pfizer, and MindMaze and on the editorial board of Stroke. His institutions have received an educational grant from Pfizer, compensation from Stago for educational efforts and research support from Daiichi-Sankyo, the Science Funds [Wissenschaftsfonds] of the University Hospital

For peer review only - http://bmjopen.bmj.com/site/about/guidelines.xhtml

Basel, the University Basel, the “Freiwillige Akademische Gesellschaft Basel”, the Swiss Heart Foundation, and the Swiss National Science Foundation.

PL has received funding for travel or speaker honoraria from Bayer, Boehringer Ingelheim, and has served on scientific advisory boards for Bayer, Boehringer Ingelheim, Pfizer/BMS, Amgen, and Medtronic. His institutions have received research support from Bayer, and Sanofi, the Science Funds [Wissenschaftsfonds] of the University Hospital Basel, the Swiss Heart Foundation, and the Swiss National Science Foundation, and the University Hspital Basel foundation “propatient”.

CHN has received funding for travel or speaker honoraria from Bayer, Boehringer Ingelheim, Pfizer Pharma, Bristol-Myers Squibb, Gore and Ass. and Sanofi. His institutions have received research support or grants from the German Federal Ministry of Research and Education (BMBF) namely the “Center for Stroke Research Berlin, CSB”, the “Deutsches Zentrum für Herz-Kreislauf Forschung (DZHK)”, “Deutsches Zentrum für neurodegenerative Erkrankungen (DZNE)”.

SW has received speaker honoraria from Amgen, travel honoraria from Bayer and a research grant from Boehringer Ingelheim and academic grants from the Swiss National Science Foundation, the UZH (Clinical Research Priority Program Stroke), the Swiss Heart foundation and the Olga Mayenfish foundation.

AZ has received speaker fees and consulting fees from Boehringer-Ingelheim, Medtronic, Cerenovus and advisory board from Daiichi Sankyo and Boehringer-Ingelheim and Stryker.

MB is on the editorial board for Springer as Co-editor of "Clinical Neuroradiology". He serves on advisory boards for Boehringer, Vascular Dynamics and BBRaun. He received speaker honoraria from Guerbet, Bayer, Novartis, Codman, Roche, Teva, Grifols and Merck. He

For peer review only - http://bmjopen.bmj.com/site/about/guidelines.xhtml

receiverd research support from Novartis, Guerbet, Siemens, Hopp foundation, the European Union, and from Deutsche Forschungs Gesellschaft."

MM is consultant for Acandis, Cerenovus, Medtronic, MicroVention, Route92, Stryker. He received research support from Balt, MicroVention, Siemens, Stryker.

PR is on scientific advisory boards for Bayer and Boehringer-Ingelheim not in relation to the submitted manuscript. He received funding for travel or speaker honoraria from SITS, and lecture fees and travel compensation from Boehringer Ingelheim, Bayer, BMS, Pfizer. He is on the editorial board of the journal Stroke as Associate editor (since 2010), of the journal Gefäßchirurgie (editor since 2014), and of the ESO-Journal (Editor since 2015). The University of Heidelberg received research support from Boehringer Ingelheim for the ECASS 4 study, and funding from the German research foundation for the SPACE-2 study.

REFERENCES

1. Rha JH and Saver JL. The impact of recanalization on ischemic stroke outcome: a meta-analysis. *Stroke*. 2007;38:967-73.
2. Saver JL, Goyal M, van der Lugt A, Menon BK, Majoie CB, Dippel DW, Campbell BC, Nogueira RG, Demchuk AM, Tomasello A, Cardona P, Devlin TG, Frei DF, du Mesnil de Rochemont R, Berkhemer OA, Jovin TG, Siddiqui AH, van Zwam WH, Davis SM, Castano C, Sapkota BL, Fransen PS, Molina C, van Oostenbrugge RJ, Chamorro A, Lingsma H, Silver FL, Donnan GA, Shuaib A, Brown S, Stouch B, Mitchell PJ, Davalos A, Roos YB, Hill MD and Collaborators H. Time to Treatment With Endovascular Thrombectomy and Outcomes From Ischemic Stroke: A Meta-analysis. *JAMA*. 2016;316:1279-88.
3. Berkhemer OA, Fransen PS, Beumer D, van den Berg LA, Lingsma HF, Yoo AJ, Schonewille WJ, Vos JA, Nederkoorn PJ, Wermer MJ, van Walderveen MA, Staals J, Hofmeijer J, van Oostayen JA, Lycklama a Nijeholt GJ, Boiten J, Brouwer PA, Emmer BJ, de Bruijn SF, van Dijk LC, Kappelle LJ, Lo RH, van Dijk EJ, de Vries J, de Kort PL, van Rooij WJ, van den Berg JS, van Hasselt BA, Aerden LA, Dallinga RJ, Visser MC, Bot JC, Vroomen PC, Eshghi O, Schreuder TH, Heijboer RJ, Keizer K, Tielbeek AV, den Hertog HM, Gerrits DG, van den Berg-Vos RM, Karas GB, Steyerberg EW, Flach HZ, Marquering HA, Sprengers ME, Jenniskens SF, Beenen LF, van den Berg R, Koudstaal PJ, van Zwam WH, Roos YB, van der Lugt A, van Oostenbrugge RJ, Majoie CB, Dippel DW and Investigators MC. A randomized trial of intraarterial treatment for acute ischemic stroke. *N Engl J Med*. 2015;372:11-20.
4. Goyal M, Demchuk AM, Menon BK, Eesa M, Rempel JL, Thornton J, Roy D, Jovin TG, Willinsky RA, Sapkota BL, Dowlatshahi D, Frei DF, Kamal NR, Montanera WJ, Poppe AY, Ryckborst KJ, Silver FL, Shuaib A, Tampieri D, Williams D, Bang OY, Baxter BW, Burns PA, Choe H, Heo JH, Holmstedt CA, Jankowitz B,

For peer review only - http://bmjopen.bmj.com/site/about/guidelines.xhtml

Kelly M, Linares G, Mandzia JL, Shankar J, Sohn SI, Swartz RH, Barber PA, Coutts SB, Smith EE, Morrish WF, Weill A, Subramaniam S, Mitha AP, Wong JH, Lowerison MW, Sajobi TT, Hill MD and Investigators ET. Randomized assessment of rapid endovascular treatment of ischemic stroke. *N Engl J Med*. 2015;372:1019-30.
5. Campbell BC, Mitchell PJ, Kleinig TJ, Dewey HM, Churilov L, Yassi N, Yan B, Dowling RJ, Parsons MW, Oxley TJ, Wu TY, Brooks M, Simpson MA, Miteff F, Levi CR, Krause M, Harrington TJ, Faulder KC, Steinfort BS, Priglinger M, Ang T, Scroop R, Barber PA, McGuinness B, Wijeratne T, Phan TG, Chong W, Chandra RV, Bladin CF, Badve M, Rice H, de Villiers L, Ma H, Desmond PM, Donnan GA, Davis SM and Investigators E-I. Endovascular therapy for ischemic stroke with perfusion-imaging selection. *N Engl J Med*. 2015;372:1009-18.
6. Saver JL, Goyal M, Bonafe A, Diener HC, Levy EI, Pereira VM, Albers GW, Cognard C, Cohen DJ, Hacke W, Jansen O, Jovin TG, Mattle HP, Nogueira RG, Siddiqui AH, Yavagal DR, Baxter BW, Devlin TG, Lopes DK, Reddy VK, du Mesnil de Rochemont R, Singer OC, Jahan R and Investigators SP. Stent-retriever thrombectomy after intravenous t-PA vs. t-PA alone in stroke. *N Engl J Med*. 2015;372:2285-95.
7. Jovin TG, Chamorro A, Cobo E, de Miquel MA, Molina CA, Rovira A, San Roman L, Serena J, Abilleira S, Ribo M, Millan M, Urra X, Cardona P, Lopez-Cancio E, Tomasello A, Castano C, Blasco J, Aja L, Dorado L, Quesada H, Rubiera M, Hernandez-Perez M, Goyal M, Demchuk AM, von Kummer R, Gallofre M, Davalos A and Investigators RT. Thrombectomy within 8 hours after symptom onset in ischemic stroke. *N Engl J Med*. 2015;372:2296-306.
8. Muir KW, Ford GA, Messow CM, Ford I, Murray A, Clifton A, Brown MM, Madigan J, Lenthall R, Robertson F, Dixit A, Cloud GC, Wardlaw J, Freeman J, White P and Investigators P. Endovascular therapy for acute ischaemic stroke: the Pragmatic Ischaemic Stroke Thrombectomy Evaluation (PISTE) randomised, controlled trial. *J Neurol Neurosurg Psychiatry*. 2017;88:38-44.
9. Bracard S, Ducrocq X, Mas JL, Soudant M, Oppenheim C, Moulin T, Guillemin F and investigators T. Mechanical thrombectomy after intravenous alteplase versus alteplase alone after stroke (THRACE): a randomised controlled trial. *Lancet Neurol*. 2016;15:1138-47.
10. Goyal M, Menon BK, van Zwam WH, Dippel DW, Mitchell PJ, Demchuk AM, Davalos A, Majoie CB, van der Lugt A, de Miquel MA, Donnan GA, Roos YB, Bonafe A, Jahan R, Diener HC, van den Berg LA, Levy EI, Berkhemer OA, Pereira VM, Rempel J, Millan M, Davis SM, Roy D, Thornton J, Roman LS, Ribo M, Beumer D, Stouch B, Brown S, Campbell BC, van Oostenbrugge RJ, Saver JL, Hill MD, Jovin TG and collaborators H. Endovascular thrombectomy after large-vessel ischaemic stroke: a meta-analysis of individual patient data from five randomised trials. *Lancet*. 2016;387:1723-31.
11. Meretoja A, Keshtkaran M, Saver JL, Tatlisumak T, Parsons MW, Kaste M, Davis SM, Donnan GA and Churilov L. Stroke thrombolysis: save a minute, save a day. *Stroke*. 2014;45:1053-8.
12. Meretoja A, Keshtkaran M, Tatlisumak T, Donnan GA and Churilov L. Endovascular therapy for ischemic stroke: Save a minute-save a week. *Neurology*. 2017;88:2123-2127.
13. Emberson J, Lees KR, Lyden P, Blackwell L, Albers G, Bluhmki E, Brott T, Cohen G, Davis S, Donnan G, Grotta J, Howard G, Kaste M, Koga M, von Kummer R, Lansberg M, Lindley RI, Murray G, Olivot JM, Parsons M, Tilley B, Toni D, Toyoda K, Wahlgren N, Wardlaw J, Whiteley W, del Zoppo GJ, Baigent C, Sandercock P, Hacke W and Stroke Thrombolysis Trialists' Collaborative G. Effect of treatment delay, age, and stroke severity on the effects of intravenous thrombolysis with alteplase for acute ischaemic stroke: a meta-analysis of individual patient data from randomised trials. *Lancet*. 2014;384:1929-35.
14. Wahlgren N, Moreira T, Michel P, Steiner T, Jansen O, Cognard C, Mattle HP, van Zwam W, Holmin S, Tatlisumak T, Petersson J, Caso V, Hacke W, Mazighi M, Arnold M, Fischer U, Szikora I, Pierot L, Fiehler J, Gralla J, Fazekas F, Lees KR, Eso-Ksu EEE and Ean. Mechanical thrombectomy in acute ischemic stroke: Consensus statement by ESO-Karolinska Stroke Update 2014/2015, supported by ESO, ESMINT, ESNR and EAN. *Int J Stroke*. 2016;11:134-47.

For peer review only - http://bmjopen.bmj.com/site/about/guidelines.xhtml

15. Powers WJ, Rabinstein AA, Ackerson T, Adeoye OM, Bambakidis NC, Becker K, Biller J, Brown M, Demaerschalk BM, Hoh B, Jauch EC, Kidwell CS, Leslie-Mazwi TM, Ovbiagele B, Scott PA, Sheth KN, Southerland AM, Summers DV, Tirschwell DL and American Heart Association Stroke C. 2018 Guidelines for the Early Management of Patients With Acute Ischemic Stroke: A Guideline for Healthcare Professionals From the American Heart Association/American Stroke Association. *Stroke*. 2018;49:e46-e110.
16. Turc G, Bhogal P, Fischer U, Khatri P, Lobotesis K, Mazighi M, Schellinger PD, Toni D, de Vries J, White P and Fiehler J. European Stroke Organisation (ESO) - European Society for Minimally Invasive Neurological Therapy (ESMINT) Guidelines on Mechanical Thrombectomy in Acute Ischaemic StrokeEndorsed by Stroke Alliance for Europe (SAFE). *Eur Stroke J*. 2019;4:6-12.
17. Nogueira RG, Jadhav AP, Haussen DC, Bonafe A, Budzik RF, Bhuva P, Yavagal DR, Ribo M, Cognard C, Hanel RA, Sila CA, Hassan AE, Millan M, Levy EI, Mitchell P, Chen M, English JD, Shah QA, Silver FL, Pereira VM, Mehta BP, Baxter BW, Abraham MG, Cardona P, Veznedaroglu E, Hellinger FR, Feng L, Kirmani JF, Lopes DK, Jankowitz BT, Frankel MR, Costalat V, Vora NA, Yoo AJ, Malik AM, Furlan AJ, Rubiera M, Aghaebrahim A, Olivot JM, Tekle WG, Shields R, Graves T, Lewis RJ, Smith WS, Liebeskind DS, Saver JL, Jovin TG and Investigators DT. Thrombectomy 6 to 24 Hours after Stroke with a Mismatch between Deficit and Infarct. *N Engl J Med*. 2018;378:11-21.
18. Albers GW, Marks MP, Kemp S, Christensen S, Tsai JP, Ortega-Gutierrez S, McTaggart RA, Torbey MT, Kim-Tenser M, Leslie-Mazwi T, Sarraj A, Kasner SE, Ansari SA, Yeatts SD, Hamilton S, Mlynash M, Heit JJ, Zaharchuk G, Kim S, Carrozzella J, Palesch YY, Demchuk AM, Bammer R, Lavori PW, Broderick JP, Lansberg MG and Investigators D. Thrombectomy for Stroke at 6 to 16 Hours with Selection by Perfusion Imaging. *N Engl J Med*. 2018;378:708-718.
19. Goyal L, Govindan A, Sheth RA, Nardi V, Blaszkowsky LS, Faris JE, Clark JW, Ryan DP, Kwak EL, Allen JN, Murphy JE, Saha SK, Hong TS, Wo JY, Ferrone CR, Tanabe KK, Chong DQ, Deshpande V, Borger DR, Iafrate AJ, Bardeesy N, Zheng H and Zhu AX. Prognosis and Clinicopathologic Features of Patients With Advanced Stage Isocitrate Dehydrogenase (IDH) Mutant and IDH Wild-Type Intrahepatic Cholangiocarcinoma. *Oncologist*. 2015;20:1019-27.
20. Scheitz JF, Gensicke H, Zinkstok SM, Curtze S, Arnold M, Hametner C, Pezzini A, Turc G, Zini A, Padjen V, Wegener S, Nordanstig A, Kellert L, Kagi G, Bejot Y, Michel P, Leys D, Nolte CH, Nederkoorn PJ, Engelter ST and collaboration T. Cohort profile: Thrombolysis in Ischemic Stroke Patients (TRISP): a multicentre research collaboration. *BMJ Open*. 2018;8:e023265.
21. Engelter ST, Soinne L, Ringleb P, Sarikaya H, Bordet R, Berrouschot J, Odier C, Arnold M, Ford GA, Pezzini A, Zini A, Rantanen K, Rocco A, Bonati LH, Kellert L, Strbian D, Stoll A, Meier N, Michel P, Baumgartner RW, Leys D, Tatlisumak T and Lyrer PA. IV thrombolysis and statins. *Neurology*. 2011;77:888-95.
22. Waje-Andreassen U, Nabavi DG, Engelter ST, Dippel DW, Jenkinson D, Skoda O, Zini A, Orken DN, Staikov I and Lyrer P. European Stroke Organisation certification of stroke units and stroke centres. *Eur Stroke J*. 2018;3:220-226.
23. Zaidat OO, Yoo AJ, Khatri P, Tomsick TA, von Kummer R, Saver JL, Marks MP, Prabhakaran S, Kallmes DF, Fitzsimmons BF, Mocco J, Wardlaw JM, Barnwell SL, Jovin TG, Linfante I, Siddiqui AH, Alexander MJ, Hirsch JA, Wintermark M, Albers G, Woo HH, Heck DV, Lev M, Aviv R, Hacke W, Warach S, Broderick J, Derdeyn CP, Furlan A, Nogueira RG, Yavagal DR, Goyal M, Demchuk AM, Bendszus M, Liebeskind DS, Cerebral Angiographic Revascularization Grading C, group SRw and Force STiCIT. Recommendations on angiographic revascularization grading standards for acute ischemic stroke: a consensus statement. *Stroke*. 2013;44:2650-63.
24. Hacke W, Kaste M, Fieschi C, von Kummer R, Davalos A, Meier D, Larrue V, Bluhmki E, Davis S, Donnan G, Schneider D, Diez-Tejedor E and Trouillas P. Randomised double-blind placebo-controlled trial

For peer review only - http://bmjopen.bmj.com/site/about/guidelines.xhtml

of thrombolytic therapy with intravenous alteplase in acute ischaemic stroke (ECASS II). Second European-Australasian Acute Stroke Study Investigators. *Lancet*. 1998;352:1245-51.
25. National Institute of Neurological D and Stroke rt PASSG. Tissue plasminogen activator for acute ischemic stroke. *N Engl J Med*. 1995;333:1581-7.
26. Wahlgren N, Ahmed N, Davalos A, Ford GA, Grond M, Hacke W, Hennerici MG, Kaste M, Kuelkens S, Larrue V, Lees KR, Roine RO, Soinne L, Toni D, Vanhooren G and investigators S-M. Thrombolysis with alteplase for acute ischaemic stroke in the Safe Implementation of Thrombolysis in Stroke-Monitoring Study (SITS-MOST): an observational study. *Lancet*. 2007;369:275-82.
27. Neuberger U, Mohlenbruch MA, Herweh C, Ulfert C, Bendszus M and Pfaff J. Classification of Bleeding Events: Comparison of ECASS III (European Cooperative Acute Stroke Study) and the New Heidelberg Bleeding Classification. *Stroke*. 2017;48:1983-1985.
28. Tatlisumak T. Implication of the recent positive endovascular intervention trials for organizing acute stroke care: European perspective. *Stroke*. 2015;46:1468-73.
29. Liu X, Xu G, Liu Y, Zhu W, Ma M, Xiong Y, Zi W, Dai Q, Leung T, Yan B, Davis S, Liebeskind DS, Pereira VM, Nogueira RG and Investigators BT. Acute basilar artery occlusion: Endovascular Interventions versus Standard Medical Treatment (BEST) Trial-Design and protocol for a randomized, controlled, multicenter study. *Int J Stroke*. 2017;12:779-785.
30. van der Hoeven EJ, Schonewille WJ, Vos JA, Algra A, Audebert HJ, Berge E, Ciccone A, Mazighi M, Michel P, Muir KW, Obach V, Puetz V, Wijman CA, Zini A, Kappelle JL and Group BS. The Basilar Artery International Cooperation Study (BASICS): study protocol for a randomised controlled trial. *Trials*. 2013;14:200.
31. Singer OC, Berkefeld J, Nolte CH, Bohner G, Haring HP, Trenkler J, Groschel K, Muller-Forell W, Niederkorn K, Deutschmann H, Neumann-Haefelin T, Hohmann C, Bussmeyer M, Mpotsaris A, Stoll A, Bormann A, Brenck J, Schlamann MU, Jander S, Turowski B, Petzold GC, Urbach H, Liebeskind DS and Group ES. Mechanical recanalization in basilar artery occlusion: the ENDOSTROKE study. *Ann Neurol*. 2015;77:415-24.
32. Singer OC, Haring HP, Trenkler J, Nolte CH, Bohner G, Neumann-Haefelin T, Hofmann E, Reich A, Wiesmann M, Niederkorn K, Deutschmann H, Bussmeyer M, Mpotsaris A, Stoll A, Bormann A, Petzold GC, Urbach H, Jander S, Turowski B, Weimar C, Schlamann M, Groschel K, Boor S, Berkefeld J and Group ES. Periprocedural aspects in mechanical recanalization for acute stroke: data from the ENDOSTROKE registry. *Neuroradiology*. 2013;55:1143-51.
33. Wollenweber FA, Tiedt S, Alegiani A, Alber B, Bangard C, Berrouschot J, Bode FJ, Boeckh-Behrens T, Bohner G, Bormann A, Braun M, Dorn F, Eckert B, Flottmann F, Hamann GF, Henn KH, Herzberg M, Kastrup A, Kellert L, Kraemer C, Krause L, Lehm M, Liman J, Lowens S, Mpotsaris A, Papanagiotou P, Petersen M, Petzold GC, Pfeilschifter W, Psychogios MN, Reich A, von Rennenberg R, Rother J, Schafer JH, Siebert E, Siedow A, Solymosi L, Thonke S, Wagner M, Wunderlich S, Zweynert S, Nolte CH, Gerloff C, Thomalla G, Dichgans M and Fiehler J. Functional Outcome Following Stroke Thrombectomy in Clinical Practice. *Stroke*. 2019;50:2500-2506.
34. Heuschmann PU, Biegler MK, Busse O, Elsner S, Grau A, Hasenbein U, Hermanek P, Janzen RW, Kolominsky-Rabas PL, Kraywinkel K, Lowitzsch K, Misselwitz B, Nabavi DG, Otten K, Pientka L, von Reutern GM, Ringelstein EB, Sander D, Wagner M and Berger K. Development and implementation of evidence-based indicators for measuring quality of acute stroke care: the Quality Indicator Board of the German Stroke Registers Study Group (ADSR). *Stroke*. 2006;37:2573-8.
35. group ISTc, Sandercock P, Wardlaw JM, Lindley RI, Dennis M, Cohen G, Murray G, Innes K, Venables G, Czlonkowska A, Kobayashi A, Ricci S, Murray V, Berge E, Slot KB, Hankey GJ, Correia M, Peeters A, Matz K, Lyrer P, Gubitz G, Phillips SJ and Arauz A. The benefits and harms of intravenous thrombolysis with recombinant tissue plasminogen activator within 6 h of acute ischaemic stroke (the third international stroke trial [IST-3]): a randomised controlled trial. *Lancet*. 2012;379:2352-63.

For peer review only

For peer review only - http://bmjopen.bmj.com/site/about/guidelines.xhtml

36. Gensicke H, Strbian D, Zinkstok SM, Scheitz JF, Bill O, Hametner C, Moulin S, Zini A, Kagi G, Pezzini A, Padjen V, Bejot Y, Corbiere S, Zonneveld TP, Seiffge DJ, Roos YB, Traenka C, Putaala J, Peters N, Bonati LH, Curtze S, Erdur H, Sibolt G, Koch P, Vandelli L, Ringleb P, Leys D, Cordonnier C, Michel P, Nolte CH, Lyrer PA, Tatlisumak T, Nederkoorn PJ, Engelter ST and Thrombolysis in Stroke Patients C. Intravenous Thrombolysis in Patients Dependent on the Daily Help of Others Before Stroke. *Stroke*. 2016;47:450-6.

37. Zinkstok SM, Vergouwen MD, Engelter ST, Lyrer PA, Bonati LH, Arnold M, Mattle HP, Fischer U, Sarikaya H, Baumgartner RW, Georgiadis D, Odier C, Michel P, Putaala J, Griebe M, Wahlgren N, Ahmed N, van Geloven N, de Haan RJ and Nederkoorn PJ. Safety and functional outcome of thrombolysis in dissection-related ischemic stroke: a meta-analysis of individual patient data. *Stroke*. 2011;42:2515-20.

38. Gensicke H, Zinkstok SM, Roos YB, Seiffge DJ, Ringleb P, Artto V, Putaala J, Haapaniemi E, Leys D, Bordet R, Michel P, Odier C, Berrouschot J, Arnold M, Heldner MR, Zini A, Bigliardi G, Padjen V, Peters N, Pezzini A, Schindler C, Sarikaya H, Bonati LH, Tatlisumak T, Lyrer PA, Nederkoorn PJ and Engelter ST. IV thrombolysis and renal function. *Neurology*. 2013;81:1780-8.

39. Gensicke H, Wicht A, Bill O, Zini A, Costa P, Kagi G, Stark R, Seiffge DJ, Traenka C, Peters N, Bonati LH, Giovannini G, De Marchis GM, Poli L, Polymeris A, Vanacker P, Sarikaya H, Lyrer PA, Pezzini A, Vandelli L, Michel P, Engelter ST and Thrombolysis in Stroke Patients c. Impact of body mass index on outcome in stroke patients treated with intravenous thrombolysis. *Eur J Neurol*. 2016;23:1705-1712.

40. Scheitz JF, Seiffge DJ, Tutuncu S, Gensicke H, Audebert HJ, Bonati LH, Fiebach JB, Tranka C, Lyrer PA, Endres M, Engelter ST and Nolte CH. Dose-related effects of statins on symptomatic intracerebral hemorrhage and outcome after thrombolysis for ischemic stroke. *Stroke*. 2014;45:509-14.

41. Zinkstok SM, Engelter ST, Gensicke H, Lyrer PA, Ringleb PA, Artto V, Putaala J, Haapaniemi E, Tatlisumak T, Chen Y, Leys D, Sarikaya H, Michel P, Odier C, Berrouschot J, Arnold M, Heldner MR, Zini A, Fioravanti V, Padjen V, Beslac-Bumbasirevic L, Pezzini A, Roos YB and Nederkoorn PJ. Safety of thrombolysis in stroke mimics: results from a multicenter cohort study. *Stroke*. 2013;44:1080-4.

42. Seiffge DJ, Hooff RJ, Nolte CH, Bejot Y, Turc G, Ikenberg B, Berge E, Persike M, Dequatre-Ponchelle N, Strbian D, Pfeilschifter W, Zini A, Tveiten A, Naess H, Michel P, Sztajzel R, Luft A, Gensicke H, Traenka C, Hert L, Scheitz JF, De Marchis GM, Bonati LH, Peters N, Charidimou A, Werring DJ, Palm F, Reinhard M, Niesen WD, Nagao T, Pezzini A, Caso V, Nederkoorn PJ, Kagi G, von Hessling A, Padjen V, Cordonnier C, Erdur H, Lyrer PA, Brouns R, Steiner T, Tatlisumak T, Engelter ST and Group* NS. Recanalization therapies in acute ischemic stroke patients: impact of prior treatment with novel oral anticoagulants on bleeding complications and outcome. *Circulation*. 2015;132:1261-9.

43. Gensicke H, Al Sultan AS, Strbian D, Hametner C, Zinkstok SM, Moulin S, Bill O, Zini A, Padjen V, Kagi G, Pezzini A, Seiffge DJ, Traenka C, Raty S, Amiri H, Zonneveld TP, Lachenmeier R, Polymeris A, Roos YB, Gumbinger C, Jovanovic DR, Curtze S, Sibolt G, Vandelli L, Ringleb PA, Leys D, Cordonnier C, Michel P, Lyrer PA, Peters N, Tatlisumak T, Nederkoorn PJ, Engelter ST and Thrombolysis in Stroke Patients C. Intravenous thrombolysis and platelet count. *Neurology*. 2018;90:e690-e697.

44. Scheitz JF, Turc G, Kujala L, Polymeris AA, Heldner MR, Zonneveld TP, Erdur H, Curtze S, Traenka C, Breniere C, Wiest R, Rocco A, Sibolt G, Gensicke H, Endres M, Martinez-Majander N, Bejot Y, Nederkoorn PJ, Oppenheim C, Arnold M, Engelter ST, Strbian D, Nolte CH and Collaboration T. Intracerebral Hemorrhage and Outcome After Thrombolysis in Stroke Patients Using Selective Serotonin-Reuptake Inhibitors. *Stroke*. 2017;48:3239-3244.

45. Polymeris AA, Curtze S, Erdur H, Hametner C, Heldner MR, Groot AE, Zini A, Bejot Y, Dietrich A, Martinez-Majander N, von Rennenberg R, Gumbinger C, Schaedelin S, De Marchis GM, Thilemann S, Traenka C, Lyrer PA, Bonati LH, Wegener S, Ringleb PA, Tatlisumak T, Nolte CH, Scheitz JF, Arnold M, Strbian D, Nederkoorn PJ, Gensicke H, Engelter ST and Collaborators T. Intravenous Thrombolysis for Suspected Ischemic Stroke with Seizure at Onset. *Ann Neurol*. 2019.

For peer review only - http://bmjopen.bmj.com/site/about/guidelines.xhtml

46. Strbian D, Ringleb P, Michel P, Breuer L, Ollikainen J, Murao K, Seiffge DJ, Jung S, Obach V, Weder B, Eskandari A, Gensicke H, Chamorro A, Mattle HP, Engelter S, Leys D, Numminen H, Kohrmann M, Hacke W and Tatlisumak T. Ultra-early intravenous stroke thrombolysis: do all patients benefit similarly? *Stroke*. 2013;44:2913-6.
47. Strbian D, Michel P, Ringleb P, Numminen H, Breuer L, Bodenant M, Seiffge DJ, Jung S, Obach V, Weder B, Tiainen M, Eskandari A, Gumbinger C, Gensicke H, Chamorro A, Mattle HP, Engelter ST, Leys D, Kohrmann M, Parkkila AK, Hacke W and Tatlisumak T. Relationship between onset-to-door time and door-to-thrombolysis time: a pooled analysis of 10 dedicated stroke centers. *Stroke*. 2013;44:2808-13.
48. Zonneveld TP, Curtze S, Zinkstok SM, Gensicke H, Moulin S, Scheitz JF, Seiffge DJ, Hametner C, Heldner MR, Traenka C, Erdur H, Baharoglu I, Martinez-Majander N, Pezzini A, Zini A, Padjen V, Correia PN, Strbian D, Michel P, Bejot Y, Arnold M, Leys D, Ringleb PA, Tatlisumak T, Nolte CH, Engelter ST, Nederkoorn PJ and collaborators T. Non-office-hours admission affects intravenous thrombolysis treatment times and clinical outcome. *J Neurol Neurosurg Psychiatry*. 2018;89:1005-1007.
49. Strbian D, Engelter S, Michel P, Meretoja A, Sekoranja L, Ahlhelm FJ, Mustanoja S, Kuzmanovic I, Sairanen T, Forss N, Cordier M, Lyrer P, Kaste M and Tatlisumak T. Symptomatic intracranial hemorrhage after stroke thrombolysis: the SEDAN score. *Ann Neurol*. 2012;71:634-41.
50. Strbian D, Meretoja A, Ahlhelm FJ, Pitkaniemi J, Lyrer P, Kaste M, Engelter S and Tatlisumak T. Predicting outcome of IV thrombolysis-treated ischemic stroke patients: the DRAGON score. *Neurology*. 2012;78:427-32.
51. Strbian D, Seiffge DJ, Breuer L, Numminen H, Michel P, Meretoja A, Coote S, Bordet R, Obach V, Weder B, Jung S, Caso V, Curtze S, Ollikainen J, Lyrer PA, Eskandari A, Mattle HP, Chamorro A, Leys D, Bladin C, Davis SM, Kohrmann M, Engelter ST and Tatlisumak T. Validation of the DRAGON score in 12 stroke centers in anterior and posterior circulation. *Stroke*. 2013;44:2718-21.
52. Curtze S, Haapaniemi E, Melkas S, Mustanoja S, Putaala J, Sairanen T, Sibolt G, Tiainen M, Tatlisumak T and Strbian D. White Matter Lesions Double the Risk of Post-Thrombolytic Intracerebral Hemorrhage. *Stroke*. 2015;46:2149-55.
53. Barber PA, Demchuk AM, Zhang J and Buchan AM. Validity and reliability of a quantitative computed tomography score in predicting outcome of hyperacute stroke before thrombolytic therapy. ASPECTS Study Group. Alberta Stroke Programme Early CT Score. *Lancet*. 2000;355:1670-4.
54. Parsons M, Spratt N, Bivard A, Campbell B, Chung K, Miteff F, O'Brien B, Bladin C, McElduff P, Allen C, Bateman G, Donnan G, Davis S and Levi C. A randomized trial of tenecteplase versus alteplase for acute ischemic stroke. *N Engl J Med*. 2012;366:1099-107.
55. Campbell BCV, Mitchell PJ, Churilov L, Yassi N, Kleinig TJ, Dowling RJ, Yan B, Bush SJ, Dewey HM, Thijs V, Scroop R, Simpson M, Brooks M, Asadi H, Wu TY, Shah DG, Wijeratne T, Ang T, Miteff F, Levi CR, Rodrigues E, Zhao H, Salvaris P, Garcia-Esperon C, Bailey P, Rice H, de Villiers L, Brown H, Redmond K, Leggett D, Fink JN, Collecutt W, Wong AA, Muller C, Coulthard A, Mitchell K, Clouston J, Mahady K, Field D, Ma H, Phan TG, Chong W, Chandra RV, Slater LA, Krause M, Harrington TJ, Faulder KC, Steinfort BS, Bladin CF, Sharma G, Desmond PM, Parsons MW, Donnan GA, Davis SM and Investigators E-IT. Tenecteplase versus Alteplase before Thrombectomy for Ischemic Stroke. *N Engl J Med*. 2018;378:1573-1582.
56. Ma H, Campbell BCV, Parsons MW, Churilov L, Levi CR, Hsu C, Kleinig TJ, Wijeratne T, Curtze S, Dewey HM, Miteff F, Tsai CH, Lee JT, Phan TG, Mahant N, Sun MC, Krause M, Sturm J, Grimley R, Chen CH, Hu CJ, Wong AA, Field D, Sun Y, Barber PA, Sabet A, Jannes J, Jeng JS, Clissold B, Markus R, Lin CH, Lien LM, Bladin CF, Christensen S, Yassi N, Sharma G, Bivard A, Desmond PM, Yan B, Mitchell PJ, Thijs V, Carey L, Meretoja A, Davis SM, Donnan GA and Investigators E. Thrombolysis Guided by Perfusion Imaging up to 9 Hours after Onset of Stroke. *N Engl J Med*. 2019;380:1795-1803.
57. Warach SJ, Luby M, Albers GW, Bammer R, Bivard A, Campbell BC, Derdeyn C, Heit JJ, Khatri P, Lansberg MG, Liebeskind DS, Majoie CB, Marks MP, Menon BK, Muir KW, Parsons MW, Vagal A, Yoo AJ,

For peer review only - http://bmjopen.bmj.com/site/about/guidelines.xhtml

Alexandrov AV, Baron JC, Fiorella DJ, Furlan AJ, Puig J, Schellinger PD, Wintermark M, Stroke Imaging R and Investigators VI-I. Acute Stroke Imaging Research Roadmap III Imaging Selection and Outcomes in Acute Stroke Reperfusion Clinical Trials: Consensus Recommendations and Further Research Priorities. *Stroke*. 2016;47:1389-98.

For peer review only

For peer review only - http://bmjopen.bmj.com/site/about/guidelines.xhtml

Table 1. Universal standards and requirements for the databases and centers contributing to EVA-TRISP registry*.

- Prospective registry of consecutive patients with systematic check-up of missing cases.
- Comprehensive collection of baseline characteristics according to consensus definitions stated in this and the previous methodology papers.
- Prospective assessment of hemorrhagic complications (symptomatic intracerebral hemorrhage according to ECASS II criteria) and functional outcome at 3 and 12 months (according to the modified Rankin Scale; either telephone interview, postal questionnaire, or follow-up visit).
- Approval of institutional review board to maintain the respective EVT database and to obtain 3- and 12-month follow-up data.
- EVA-TRISP centers are comprehensive stroke centers with high-volume EVT applications - typically university hospitals or closely affiliated to university hospitals.
- Treatment of acute ischemic stroke patients with EVT according to guidelines valid at the relevant time or documentation of deviation therefrom.

* EVA-TRISP welcomes participation and project proposals of further centers fulfilling the commitment and the outlined requirements.

For peer review only

For peer review only - http://bmjopen.bmj.com/site/about/guidelines.xhtml

Table 2. EVA-TRISP centers, time period, number of endovascular treatments done, and population-base for EVT (in alphabetical order).

City	No of stroke EVT (Jan 2015 to Dec 2019)
Amsterdam	864
Basel	413
Belgrade	136[1]
Berlin	480[2]
Bern	1422
Bremen	Estimation: 200/year
Brescia	412
Bologna	395
Goettingen	396
Gothenburg	1097
Heidelberg	1500
Helsinki	796[2]
Jerusalem	249
Larissa	-
Lausanne	732
Lille	1806
Modena	489
Munich	600
St. Gallen	490
Zurich	Estimation: 500

[1]Jan 2018-Dec 2019
[2]Nov 2015-Dec 2019

For peer review only

For peer review only - http://bmjopen.bmj.com/site/about/guidelines.xhtml

Figure 1. EVA-TRISP centers.

For peer review only

For peer review only - http://bmjopen.bmj.com/site/about/guidelines.xhtml

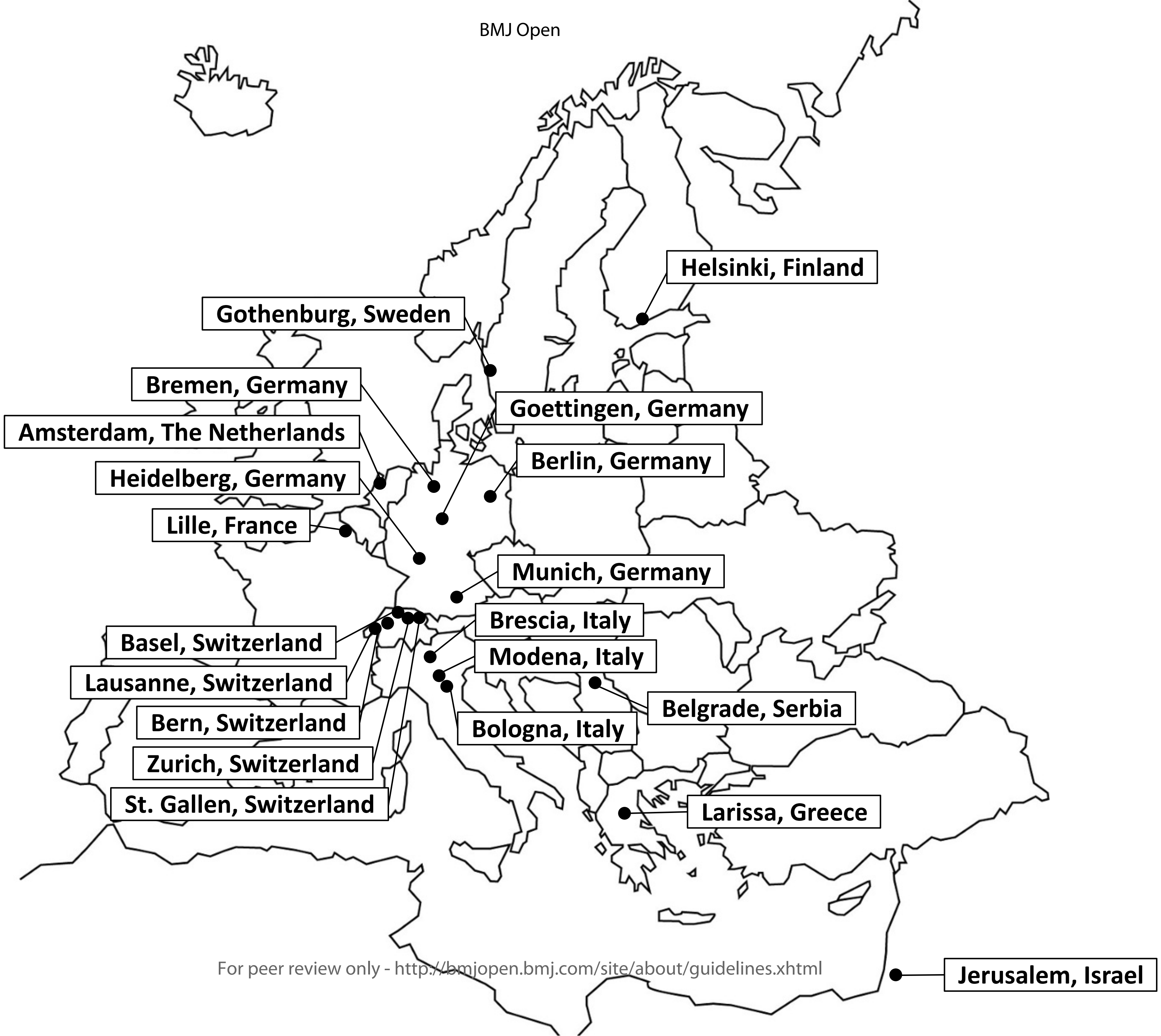
Helsinki, Finland
Gothenburg, Sweden
Bremen, Germany
Goettingen, Germany
Amsterdam, The Netherlands
Berlin, Germany
Heidelberg, Germany
Lille, France
Munich, Germany
Brescia, Italy
Basel, Switzerland
Modena, Italy
Lausanne, Switzerland
Belgrade, Serbia
Bern, Switzerland
Bologna, Italy
Zurich, Switzerland
St. Gallen, Switzerland
Larissa, Greece
Jerusalem, Israel

! " " # $ %& ' ( )*(# + ! , - . &/ "(&0123456748 93(:50(7;<=7>245?7;(89@29(>A(?8B049AC(

D50;70@(:)C* Sami Curtze, Kimmo Lappalainen, Nicolas Martinez-Majander, Jukka Putaala, Gerli Sibolt, Daniel Strbian, Silja Räty, Turgut Tatlisumak, Marjaana Tiainen (Helsinki University Hospital)

D970?2(:)C* Nicolas Bricout, Marie Bodenant, Regis Bordet, Charlotte Cordonnier, Nelly Dequatre, Hilde Hénon, Didier Leys, Anne-Marie Mendyk (University Lille North de France)

E29F70A(:GC* Andreas Kastrup, Panagiotis Papanagiotou (University Hospitals Bremen-Mitte and Bremen-Ost); Tim-Bastian Braemswig, Hebun Erdur, Christian H Nolte, Regina von Rennenberg, Jan F Scheitz, Georg Bohner (Charité-Universitätsmedizin, Berlin); Alex Brehm, Jan Liman, Marios Psychogios (University Medical Center Goettingen); Martin Bendszus, Christian Hametner, Markus Möhlenbruch, Peter A Ringleb (University Hospital Heidelberg); Katharina Feil, Lars Kellert, Clemens Küpper (University Hospital Munich LMU)

E92??2(:)C* George Ntaios, Dimitrios Sagris, Ioannis Ioannidis George Karagiorgas, Eftychia Kapsalaki, Marianna Vlychou (University of Thessaly)

(

&3972;(:)C* Jose Cohen, John Gomori, Ronen R Leker (Hadassah-Hebrew University Medical Center, Jerusalem)

&47;A(:HC* Mauro Magoni, Alessandro Pezzini (University Hospital Brescia); Guido Bigliardi, Luca Verganti, Stefano Vallone, Stefania Maffei (University Hospital Modena), Andrea Zini,

For peer review only - http://bmjopen.bmj.com/site/about/guidelines.xhtml

For peer review only

Luigi Simonetti, Mauro Gentile, Luigi Cirillo, Ludovica Migliaccio (IRCCS Istituto di Scienze Neurologiche di Bologna)

-=2($24=29;70@3(:)C* M Irem Baharoglu, Sophie van den Berg, Paul J Nederkoorn, Yvo B Roos, Fianne HM Spaander, Sanne M Zinkstok, Thomas P Zonneveld, Charles Majoie (Academic Medical Center Amsterdam)

/29>57(:)C* Mirjana Arsenijevic, Ivana Berisavac, Marko Ercegovac, Dejana R Jovanovic, Visnja Padjen, Predrag Stanarcevic, Maja Stefanovic Budimkic, Tamara Svabic Medjedovic, Ivan Vukasinovic, Vladimir Cvetic (Clinical Centre of Serbia, Belgrade)

/ I 2@20(:)C* Margareta Abrahamson, Arne Allard, Monica Argus, Anke Brederleu, Erik Ceder, Maria Davidson, Niclas Dehlfors, Dennis Dunker, Torsteinn Gunnarsson, Lukas Holmegaard, Mikael Jerndal, Susanna Johansson, Katarina Jood, Camilla Karlsson, Jan-Erik Karlsson, Birgitta Leiram, Miroslav Malac, Inger Nilsson, Annika Nordanstig, Petra Redfors, Alexandros Rentzos, Turgut Tatlisumak (Sahlgrenska University Hospital, Gothenburg)

/ I 54J29;70@(:GC* Leo H Bonati, Stefan T Engelter, Joachim Fladt, Henrik Gensicke, Philippe A Lyrer, Gian Marco De Marchis, Nils Peters, Alexandros Polymeris, Sebastian Thilemann, Christopher Traenka (University Hospital Basel); Marcel Arnold, Urs Fischer, Jan Gralla, Mirjam R Heldner, Hakan Sarikaya, David J Seiffge, Roland Wiest (University Hospital Bern); Olivier Bill, Ashraf Eskandari, Patrik Michel, Gaia Sirimarco (Hospitalier Universitaire Vaudois, Lausanne); Georg Kägi, Johannes Weber (Kantonsspital St. Gallen); Zsolt Kulcsar, Andreas R Luft, Susanne Wegener (University Hospital Zurich)

For peer review only - http://bmjopen.bmj.com/site/about/guidelines.xhtml

! " " # $ %& ' (KL(# + ! , - . &/ "(% ! - ! M ! / #(%N O ! &$ / (! $ %(& - # O / (

The exact list of available variables may slightly differ between centers according to the judgment of local ethics committees.

Patient history

- ! "#$
- %#&$
- ' (#)*+(, -#$. / %$
- 012#3#12#1+$3(4 , ($+ , $*+(, -#$5678 9$
- / 4*-$: ; <+ , ($; +(4 ; =$:4>(4== ; +4 , 1$3(#*#1+$; 12$-1 , ? 1$5678 9$
- / 4*-$: ; <+ , ($24 ; >#+#*$3(#*#1+$; 12$-1 , ? 1$5678 9$5678 9$
- / 4*-$: ; <+ , ($@A3#(+#1*4 , 1$3(#*#1+$; 12$-1 , ? 15678 9$
- / 4*-$: ; <+ , ($@A3#(<@ , =#*+#(, =# . 4 ; $3(#*#1+$; 12$-1 , ? 1$5678 9$
- / 4*-$: ; <+ , ($< , (, 1 ; (A$; (+#(A$24*# ; *#$3(#*#1+$; 12$-1 , ? 15678 9$
- / 4*-$: ; <+ , ($3(4 , ($4*<@# . 4<$*+(, -#$5<=414< ; =$24 ; "1 , *4*9$5678 9$
- 0:$3(4 , ($4*<@# . 4<$*+(, -#B$3(4 , ($+(# ; + . #1+$? 4+@$0CD7ECD$5678 9$
- / 4*-$: ; <+ , ($<F((#1+$* . , -41"$5 , ($*+ , 33#2$GHA9$5678$
- ' (#)0CD$F*#$, :$*+ ; +41*$5678$; *$? #==$; *$1 ; . #$; 12$2 , * ; "#9$
- ' (#)0CD$F*#$, :$; 1+4@A3#(+#1*4I#5678 ; *$? #==$; *$1 ; . #$; 12$2 , * ; "#9$
- ' (#)0CD$F*#$, :$; 1+43= ; +#=#+*5678 ; *$? #==$; *$1 ; . #$; 12$2 , * ; "#9$
- ' (#)0CD$F*#$, :$; 1+4< , ; "F= ; 1+*5678 ; *$? #==$; *$1 ; . #$; 12$2 , * ; "#9$
- ! ==$. #24< ; +4 , 1*$51 ; . #$; 12$2 , * ; "#$; *$:(##$+#&+9$

For peer review only - http://bmjopen.bmj.com/site/about/guidelines.xhtml

For peer review only

Pre-hospital

- J ; +#$, :$*+(, -#$, 1*#+$5= ; *+$*##1$? #==9$
- D4 . #$, :$*+(, -#$, 1*#+$5= ; *+$*##1$? #==9$
- J ; +#$, :$*+(, -#$, 1*#+$5:4(*+$*##1$*4< - 9$
- D4 . #$, :$*+(, -#$, 1*#+$5:4(*+$*##1$*4< - 9$
- %+(, -#$, 1*#+$? 4+1#**#2K$5678 9$
- L ; -#)F3$*+(, -#K$5678 9$
- E34=#3+4<$*#4MF(#$; +$*+(, -#$, 1*#+$5678 9$

Admission

- J ; +#$, :$@ , *34+ ; =$; ((4I ; =$
- D4 . #$, :$@ , *34+ ; =$; ((4I ; =$
- L ; *$3 ; +4#1+$+(; 1*:#((#2$:(, . $; 1 , +@#($@ , *34+ ; =K$5678 9$
- 0CD$; 2 . 414*+(; +#2$; +$, +@#($@ , *34+ ; =K$5678 9$
- 80N%%$; +$; 2 . 4**4 , 1$
- %A*+ , =4<$>= , , 2$3(#**F(#$, 1$; 2 . 4**4 , 1$0 . . N"P$
- J4 ; *+ , =4<$>= , , 2$3(#**F(#$, 1$; 2 . 4**4 , 1$0 . . N"P$
- L #4"@+$5#& ; <+$7$#*+4 . ; +#29$
- N#4"@+$5#& ; <+$7$#*+4 . ; +#29$
- Q=F< , *#$, 1$; 2 . 4**4 , 1$0 . . , =7=P$
- R(# ; +4141#$, 1$; 2 . 4**4 , 1$0F . , =7=P$

For peer review only - http://bmjopen.bmj.com/site/about/guidelines.xhtml
For peer review only

- 01+#(1;+4,1;=$8,(.;=4M#2$/;+4,$3(4,($+,$0CD$53,41+$,:$<;(#$+#*+$7$;<+F;=$=;>$I;=F#9$
- 0:$24(#<+$,(;=$;1+4<,;"F=;1+*$5JS!R9B$*3#<4:4<$#**;A7=#I#=$,:$JS!R$$$$$
- '=;+#=#+*$,1$;2.4**4,1$&TU#V$07=P$
- N#.,"=,>41$,1$;2.4**4,1$0"72=P$
- W#F-,<A+#*$,1$;2.4**4,1$&TU#V$07=P$
- R)/#;<+4I#$'(,+#41$,1$;2.4**4,1$0."7=P$

Acute interventions

- DA3#$,:$41+#(I#1+4,1$50CDB$>(42"41"B$ECD9$
- J;+#$,:$0CD$;2.414*+(;+4,1$
- D4.#$,:$0CD$;2.414*+(;+4,1$
- J,*;"#$,:$(+'!$0CD$5UXY$."7-"B$UXV$."7-"B$GZU[$,:$2,*#$,($<,.3=#+#9$
- !1"4,#2#.;$(#=;+#2$+,$0CD$56789$
- E&<=F*4,1$<(4+#(4;$0CD$5(#<#4I#2$0CDB$2#=;A*B$.;\,($3(#*+(,-#$@;124<;3B$4.;"41"$
  <,1+(;4124<;+4,1B$>=##241"$(4*-B$*+(,-#$.4**#27F1<#(+;41B$,+@#(9$
- E&<=F*4,1$<(4+#(4;$0CD$0:(##$+#&+P$
- J;+#$,:$ECD$"(,41$3F1<+F(#$
- D4.#$,:$ECD$"(,41$3F1<+F(#$
- 01+(;);(+#(4;=$+@(,.>,=A+4<$2(F"56789
- 8;.#$;12$2,*;"#$,:$41+(;);(+#(4;=$+@(,.>,=A+4<$2(F"$0:(##$+#&+P$
- Q#1#(;=$;1#*+@#*4;$2F(41"ECD56789$

For peer review only

For peer review only - http://bmjopen.bmj.com/site/about/guidelines.xhtml

- DA3#$, :$#12, I ;*<F=; ($+(#; + . #1+$51, 1#B$*+#1+$(#+(4#I#(B$; *34(; +4, 1B$24*+; =$(#+(4#I#(B$24*+; =$;*34(; +4, 1B$] ;=, , 1$; 1"4, 3=; *+AB$3#(. ;1#1+$41+(; <(; 14; =$*+#1+B$#&+(; <(; 14; =$*+#1+B$, +@#($ 0<, . >41; +4, 1$3, **4>=#P$
- 8F . >#($, :$; ++# . 3+*$:, ($#; <@$+(#; + . #1+$
- 8; . #$, :$2#I4<#5*9$
- D; 12# . $*+#1, *4*7, <<=F*4, 1$3(#*#1+56789
- E&+(; <(; 14; =$+@(, . >#<+, . A$51, B$0R ! B$C ! 9$
- E&+(; <(; 14; =$3#(. ;1#1+$*+#1+$51, B$0R ! B$C ! 9$
- S1=A$; ++# . 3+$+, $3#(:, (. ECD
- ECD$*+, 33#2$#; (=A$>#<; F*#$5414+4; +#2B$>F+$; <<#**)+,)<=, +)3(, >=# . *B$+(4#2B$>F+$; (+#(A$;=(#; 2A$(#<; 1; =4M#2B$, +@#(9$
- ECD$*+, 33#2$#; (=A$(#; *, 1$0:(##$+#&+P$
- ECD$<, . 3=4<; +4, 1*$51, B$I#**#=$3#(:, (; +4, 1B$I ; *, *3; * . B$24**#<+4, 1B$% ! N70RNB$2#I4<#$ 2#+; <@ . #1+7 . 4*3=; <# . #1+B$# . >, =4M; +4, 1$+, $1#?$+#((4+, (AB$; <<#**)*4+#$<, . 3=4<; +4, 1*B$ #; (=A$(#, <<=F*4, 1B$, +@#($0:(##$+#&+P$0<, . >41; +4, 1$3, **4>=#P$
- D4 . #$, :$ECD$#12$, :$3(, <#2F(#$

Imaging

- DA3#$, :$>; *#=41#$4 . ; "#$51, 1#BRDB ^ / 9$
- J; +#$, :$>; *#=41#$4 . ; "#$
- D4 . #$, :$>; *#=41#$4 . ; "#$

For peer review only - http://bmjopen.bmj.com/site/about/guidelines.xhtml

- D#((4+ , (A$, :$41: ; (<+4 , 1$50R ! B$ ^ R ! B$! R ! B$ ' R ! B$R#(#>#==F . B$] (; 41*+# . $0< , . >41 ; +4 , 1$ 3 , **4>=#P$
- %42#$, :$41: ; (<+4 , 1$5=#:+$; 1+#(4 , ($<4(<F= ; +4 , 1B$(4"@+$; 1+#(4 , ($<4(<F= ; +4 , 1B$3 , *+#(4 , ($<4(<F= ; +4 , 1$ 0< , . >41 ; +4 , 1$3 , **4>=#P$
- ! 1+#(4 , ($<4(<F= ; +4 , 1$! % ' ERD%$, 1$> ; *#=41#$4 . ; "#$U)TU$
- ' , *+#(4 , ($<4(<F= ; +4 , 1$! % ' ERD%$, 1$> ; *#=41#$4 . ; "#$U)TU$
- E ; (=A$4*<@# . 4<$<@ ; 1"#*41*F*3#<+#2$; (# ; $5: , < ; =$3 ; (#1<@A . ; =$@A3 , ; ++#1F ; +4 , 1B$= , **$, :$ "(; A) ? @4+#$. ; ++#($24: :#(#1+4 ; +4 , 1B$: , < ; =$#2# . ; $. ; 14:#*+#2$>A$*F=< ; =$, ($I#1+(4<F= ; ($ #: : ; <# . #1+9$5678 9$
- S<<=F2#2$I#**#=$? 4+@$@A3#(2#1*#$; (+#(A$*4"1$5)_$< , =F . 1$RJ9$5678 9$
- DA3#$, :$> ; *#=41#$; 1"4 , "(; 3@A$51 , 1#B$RD ! B$ ^ / ! 9$
- %4+#$, :$. ; 41$41+(; <(; 14 ; =$, <<=F*4 , 1$, 1$> ; *#=41#$; 1"4 , "(; 3@A$51 , 1#B$0R !)0B$0R !)W7DB$3(, &$ ^ TB$24*+ ; =$ ^ TB$ ^ HB$! R ! B$ ' R ! B$] ! B$C ! 9$
- S+@#($, <<=F*4 , 1$*4+#B$3=# ; *#$*3#<4:A$0:(##$+#&+P$
- ! 224+4 , 1 ; =$I#**#=$, <<=F*4 , 1$5A719$$
- 0:$A#*B$= , < ; +4 , 1$, :$; 224+4 , 1 ; =$I#**#=$, <<=F*4 , 1$
- 01$< ; *#$, :$41+(; <(; 14 ; =$= ; ("#$I#**#=$, <<=F*4 , 1$41$+@#$; 1+#(4 , ($<4(<F= ; +4 , 1'$< , == ; +#(; =*$, 1$ > ; *#=41#$; 1"4 , "(; 3@A$4:$; **#** ; >=#$5D ! 8$%< , (#$U)a9$
- /#=#I ; 1+$*+#1 , *4*$5_ZU [$8 ! %RED97 , <<=F*4 , 1$, :$#&+(; <(; 14 ; =$0R ! $, 1$> ; *#=41#$; 1"4 , "(; 3@A$ 5678 9$
- /#=#I ; 1+$*+#1 , *4*$5_ZU [$8 ! %RED97 , <<=F*4 , 1$, :$#&+(; <(; 14 ; =$C ! $, 1$> ; *#=41#$; 1"4 , "(; 3@A$ 5678 9$

For peer review only

For peer review only - http://bmjopen.bmj.com/site/about/guidelines.xhtml

- DA3#$,:$>;*#=41#$3#(:F*4,1$. ,2;=4+A$51,1#B$RD'B$^ / '9$
- ^4* . ;+<@$(;+4,$;<<,(241"$+,$=,<;=$. ,2;=4+4#*56789
- D,+;=$3#(:F*4,1$=#*4,1741:;(<+$<,(#$. 4* . ;+<@$(;+4,$I4*F;==A$,1$>;*#=41#$3#(:F*4,1$4 . ;"#$

  5:X4XTXHb$3#(:F*4,1$=#*4,1$4*$HU [$=;("#($+@;1$<,(#9$51F . >#(9J%!$56789
- 01$<;*#$,:$41+(;<(;14;=$=;("#$I#**#=$,<<=F*4,1$41$+@#$;1+#(4,($<4(<F=;+4,1'$<,==;+#(;=*$,1$J%!$

  5!%0D87%0/$"(;241"9$$$
- R, . 3=#+#$(#<;1;=4M;+4,1$,1$J%!$5 . D0R0bH>7a9$$56789$
- J;+#$,:$(#<;1;=4M;+4,1$,1$J%!$
- D4 . #$,:$(#<;1;=4M;+4,1$,1$J%!$

Follow up

- DA3#$,:$:4(*+$:,==,?)F3$1;+4I#$4 . ;"#$51,1#B$8RRDB$^ /09$
- J;+#$,:$:,==,?)F3$4 . ;"#$
- D4 . #$,:$:,==,?)F3$4 . ;"#$
- DA3#$,:$:4(*+$:,==,?)F3$I#**#=$4 . ;"41"$51,1#B$RD!B$^ / !)DScB$^ / !)REB$F=+(;*,F129$
- J;+#$,:$:,==,?)F3$I#**#=$4 . ;"41"$
- D4 . #$,:$:,==,?)F3$I#**#=$4 . ;"41"$
- R, . 3=#+#$(#<;1;=4M;+4,1$,1$:,==,?)F3$I#**#=$4 . ;"41"$5RD!7^ / !7d%9$56789$
- . D0R0$
- !1A$41+(;<#(#>(;=$@# . ,((@;"#$50RN9$
- c;+;=$0RN$
- %A . 3+, . ;+4<$0RN$5ER!%%)H$<(4+#(4;9$56789$

For peer review only - http://bmjopen.bmj.com/site/about/guidelines.xhtml

- S+@#($0RN$5%!NB$%JNB$#+<X9$0:(##$+#&+P$
- 80N%%$;:+#($He@$
- ./%$;:+#($a$.,1+@*$
- /#<F((#1+$4*<@#.4<$*+(,-#$,($D0!$?4+@41$a$.,1+@*$51,B$D0!5*9B$*+(,-#5*9$54:$D0!$;12$*+(,-#B$ <,F1+$*+(,-#9$
- ./%$;:+#($T$A#;($

Other

- ^,24:4#2$DS!%D$5W!!BRE541<=F241"$'cS9B$%!SB$,+@#(B$.,(#$+@;1$,1#B$F12#+#(.41#2B$ *+(,-#$.4.4<$
- /;(#$*3#<4:4<$*+(,-#$<;F*#*$+,$>#$*+F24#2$41$.,(#$2#+;4=$5<#(I4<;=$;(+#(A$24**#<+4,1B$ 41+(;<(;14;=$;(+#(A$24**#<+4,1B$#12,<;(24+4*B$I;*<F=4+4*B$<,;"F=,3;+@4#*9$

$

$

$

For peer review only - http://bmjopen.bmj.com/site/about/guidelines.xhtml

For peer review only

BMJ Open

Cohort profile: EndoVAscular treatment and ThRombolysis for Ischemic Stroke Patients (EVA-TRISP) Registry: Basis and methodology of a pan-European prospective ischemic stroke revascularization treatment registry

Journal:	*BMJ Open*
Manuscript ID	bmjopen-2020-042211.R1
Article Type:	Cohort profile
Date Submitted by the Author:	24-Apr-2021

Complete List of Authors:	Nordanstig, Annika; Sahlgrenska Academy, Department of Clinical Neurosciences Sahlgrenska Academy at University of Gothenburg; Department of Neurology, Sahlgrenska University Hospital<br>Curtze, Sami; Helsingfors universitets centralsjukhus, Department of Neurology, University of Helsinki and Helsinki University Hospital<br>Gensicke, H; University of Basel, Department of Neurology and Stroke Center, University of Basel and University Hospital Basel, Switzerland<br>Zinkstok, Sanne; Amsterdam University Medical Centres, Department of Neurology, Amsterdam University Medical Centers, the Netherlands<br>Erdur, Hebun; Charité Universitätsmedizin Berlin,<br>Karlsson, Camilla; Sahlgrenska Academy, Department of Clinical Neurosciences, Sahlgrenska Academy at University of Gothenburg<br>Karlsson, Jan-Erik; Sahlgrenska Academy, Department of Clinical Neurosciences, Sahlgrenska Academy at University of Gothenburg; Department of Neurology, Sahlgrenska University Hospital<br>Martinez-Majander , Nicolas; Helsinki University Hospital, Department of Neurology, University of Helsinki and Helsinki University Hospital<br>Sibolt, G; HYKS sairaanhoitopiiri, Department of Neurology, University of Helsinki and Helsinki University Hospital<br>Lyrer, Philippe; University Hospital Basel, Department of Neurology and Stroke Center, University of Basel and University Hospital Basel<br>Traenka, Christopher; Universitätsspital Basel, Department of Neurology and Stroke Center, University of Basel and University Hospital Basel<br>Baharoglu, Merih; Amsterdam University Medical Centres, Department of Neurology, Amsterdam University Medical Centers<br>Scheitz, Jan; Charite Universitatsmedizin Berlin Klinik fur Neurologie mit Experimenteller Neurologie, Department of Neurology and Center for Stroke Research, Charité-Universitätsmedizin Berlin<br>Bricout, Nicolas; University of Lille, Department of Neurology, University of Lille<br>Hénon, Hilde; University of Lille, Department of Neurology, University of Lille<br>Leys, Didier; University of Lille, Department of Neurology, University of Lille<br>Eskandari, Ashraf; University of Lausanne, Department of Neurology, Centre Hospitalier Universitaire Vaudois and University of Lausanne<br>Michel, Patrik; University of Lausanne, Department of Neurology, Centre Hospitalier Universitaire Vaudois and University of Lausanne

Hametner, Christian; University Hospital Heidelberg, Department of Neurology, University Hospital Heidelberg
Ringleb, Peter; University Hospital Heidelberg, Department of Neurology, University Hospital Heidelberg
Arnold, Marcel; Inselspital University Hospital Berne Department of Neurology, Inselspital, Bern University Hospital, University of Bern
Fischer, Urs; Inselspital University Hospital Bern, Department of Neurology, Inselspital, Bern University Hospital, University of Bern
Sarikaya, Hakan; Inselspital University Hospital Bern, Department of Neurology, Inselspital, Bern University Hospital, University of Bern
Seiffge, David ; Inselspital University Hospital Bern, Department of Neurology, Inselspital, Bern University Hospital, University of Bern
Pezzini, Alessandro ; Università degli Studi di Brescia, Department of Clinical and Experimental Sciences, Neurology Clinic, University of Brescia,
Zini, Andrea; IRCCS Istituto Delle Scienze Neurologiche di Bologna, Department of Neurology and Stroke Center, Maggiore Hospital, Bologna
Padjen, Visnja; Clinical Center of Serbia, Neurology Clinic, Clinical Centre of Serbia, Belgrade
Jovanovic, Dejana R.; Clinical Center of Serbia, Neurology Clinic, Clinical Centre of Serbia, Belgrade; Faculty of Medicine, University of Belgrade
Luft, Andreas; University of Zurich, Department of Neurology, University of Zürich
Wegener, Susanne; University of Zurich, Department of Neurology, University of Zürich
Kellert, Lars; Ludwig-Maximilians-Universitat Munchen, Neurology
Feil, Katharina; Ludwig-Maximilians-Universitat Munchen, Neurology and German Center for Vertigo and Balance Disorders
Kägi, Georg; Kantonsspital St Gallen, Department of Neurology, Kantonsspital St. Gallen
Rentzos, Alexandros ; Sahlgrenska Academy, Department of Radiology, Institute of clinical sciences, Sahlgrenska Academy, University of Gothenburg; Diagnostic and Interventional Neuroradiology, Sahlgrenska University Hospital and
Lappalainen, Kimmo; Helsinki University Central Hospital, Department of Radiology, Helsinki University Hospital
Leker , RR; Hebrew University Hadassah Medical School, Department of Neurology, Hadassah-Hebrew University Medical Center
Cohen, Jose; Hebrew University Hadassah Medical School, Department of Neurosurgery, Hadassah-Hebrew University Medical Center
Gomori, John; Hebrew University Hadassah Medical School, Department of Radiology, Hadassah-Hebrew University Medical Center
Brehm, Alex; Georg-August-Universitat Gottingen Universitatsmedizin, Department of Neurology, University Medical Center Goettingen
Liman, Jan; Georg-August-Universitat Gottingen Universitatsmedizin, Department of Neurology, University Medical Center Goettingen
Psychogios, Marios; Georg-August-Universitat Gottingen Universitatsmedizin, Department of Neuroradiology, University Medical Center Goettingen; Neuroradiology and Stroke Center Basel, University of Basel and University Hospital Basel
Kastrup, Andreas; University of Bremen, Department of Neurology, University Hospitals Bremen-Mitte and Bremen-Ost
Papanagiotou, Panagiotis; University of Bremen, Clinic for Diagnostic and Interventional Neuroradiology, University Hospitals Bremen-Mitte and Bremen-Ost; Dept. of Radiology, Areteion University Hospital, National and Kapodistrian University of Athens
Gralla, Jan; University of Bern, Institute of Diagnostic and Interventional Neuroradiology, Inselspital, Bern University Hospital, University of Bern
Magoni, Mauro; ASST Spedali Civili di Brescia, USD Stroke Unit and Vascular Neurology, ASST Spedali Civili Brescia, Brescia, Italy
Majoie, Charles; Amsterdam UMC - Locatie AMC, Department of

	Neuroradiology, Amsterdam University Medical Centers<br>Bohner, Georg; Charité Universitätsmedizin Berlin, Institute of Neuroradiology, Charité-Universitätsmedizin Berlin, Germany<br>Vukasinovic, Ivan; Clinical Center of Serbia, Clinic of Neurosurgery, Clinical Centre of Serbia, Belgrade<br>Cvetic, Vladimir; University of Belgrade, Faculty of Medicine, University of Belgrade; Clinic for Vascular and Endovascular Surgery, Clinical Centre of Serbia, Belgrade<br>Weber, Johannes; Kantonsspital St Gallen, Department of Radiology and Nuclear Medicine, Kantonsspital St. Gallen<br>Kulcsar, Zsolt; University Hospital Zurich, Department of Neuroradiology<br>Bendszus, Martin; UniversitatsKlinikum Heidelberg, Department of Neuroradiology<br>Möhlenbruch, Markus ; UniversitatsKlinikum Heidelberg, Department of Neuroradiology<br>Ntaios, George; University of Thessaly, Department of Internal Medicine, Faculty of Medicine, School of Health Sciences<br>Kapsalaki, Eftychia; University of Thessaly, Department of Radiology, University Hospital of Larissa, School of Medicine<br>Jood, Katarina; Sahlgrenska Academy, Department for Clinical Neuroscience and Rehabilitation; Department of Neurology, Sahlgrenska University Hospital<br>Nolte, Christian ; Charité Universitätsmedizin Berlin, Department of Neurology and Center for Stroke Research<br>Nederkoorn, Paul J; Amsterdam University Medical Centres, Department of Neurology<br>Engelter, Stefan; University of Basel, Department of Neurology and Stroke Center; Deptartment of Radiology, Areteion University Hospital, National and Kapodistrian University of Athens<br>Strbian, Daniel; University of Helsinki, Department of Neurology, University of Helsinki and Helsinki University Hospital<br>Tatlisumak, T; Sahlgrenska University Hospital, Department of Clinical Neurosciences; University of Helsinki, Department of Neurology
**Primary Subject Heading**:	Neurology
Secondary Subject Heading:	Neurology
Keywords:	Stroke < NEUROLOGY, Neurology < INTERNAL MEDICINE, Stroke medicine < INTERNAL MEDICINE

SCHOLARONE™
Manuscripts

I, the Submitting Author has the right to grant and does grant on behalf of all authors of the Work (as defined in the below author licence), an exclusive licence and/or a non-exclusive licence for contributions from authors who are: i) UK Crown employees; ii) where BMJ has agreed a CC-BY licence shall apply, and/or iii) in accordance with the terms applicable for US Federal Government officers or employees acting as part of their official duties; on a worldwide, perpetual, irrevocable, royalty-free basis to BMJ Publishing Group Ltd ("BMJ") its licensees and where the relevant Journal is co-owned by BMJ to the co-owners of the Journal, to publish the Work in this journal and any other BMJ products and to exploit all rights, as set out in our licence.

The Submitting Author accepts and understands that any supply made under these terms is made by BMJ to the Submitting Author unless you are acting as an employee on behalf of your employer or a postgraduate student of an affiliated institution which is paying any applicable article publishing charge ("APC") for Open Access articles. Where the Submitting Author wishes to make the Work available on an Open Access basis (and intends to pay the relevant APC), the terms of reuse of such Open Access shall be governed by a Creative Commons licence – details of these licences and which Creative Commons licence will apply to this Work are set out in our licence referred to above.

Other than as permitted in any relevant BMJ Author's Self Archiving Policies, I confirm this Work has not been accepted for publication elsewhere, is not being considered for publication elsewhere and does not duplicate material already published. I confirm all authors consent to publication of this Work and authorise the granting of this licence.

For peer review only

Cohort profile: EndoVAscular treatment and ThRombolysis for Ischemic Stroke Patients (EVA-TRISP) Registry: Basis and methodology of a pan-European prospective ischemic stroke revascularization treatment registry

Annika Nordanstig[1*], Sami Curtze[2*], Henrik Gensicke[3*], Sanne M Zinkstok[4*], Hebun Erdur[5*], Camilla Karlsson[1*], Jan-Erik Karlsson[1], Nicolas Martinez-Majander[2], Gerli Sibolt[2], Philippe A Lyrer[3], Christopher Traenka[3], Merih I Baharoglu[4], Jan F Scheitz[5], Nicolas Bricout[6], Hilde Hénon[6], Didier Leys[6], Ashraf Eskandari[7], Patrik Michel[7], Christian Hametner[8],Peter A Ringleb[8], Marcel Arnold[9], Urs Fischer[9], Hakan Sarikaya[9], David J Seiffge[9], Alessandro Pezzini[10], Andrea Zini[11], Visnja Padjen[12], Dejana R Jovanovic[12,13], Andreas R Luft[14], Susanne Wegener[14], Lars Kellert[15], Katharina Feil[15], Georg Kägi[16], Alexandros Rentzos[17], Kimmo Lappalainen[18], Ronen R Leker[19], Jose Cohen[20], John Gomori[21], Alex Brehm[22], Jan Liman[22], Marios Psychogios[23], Andreas Kastrup[24], Panagiotis Papanagiotou[25,26], Jan Gralla[27], Mauro Magoni[28], Charles Majoie[29], Georg Bohner[30], Ivan Vukasinovic[31], Vladimir Cvetic[13,32], Johannes Weber[33], Zsolt Kulcsar[34], Martin Bendszus[35], Markus Möhlenbruch[35], George Ntaios[36], Eftychia Kapsalaki[37], Katarina Jood[1#], Christian H Nolte[5#], Paul J Nederkoorn[4#], Stefan T Engelter[3,26#], Daniel Strbian[2#], Turgut Tatlisumak[1,2#], on behalf of EVA-TRISP Investigators

*= shared first authorship; #= shared last authorship

[1]Department of Neurology, Sahlgrenska University Hospital and Department of Clinical Neurosciences, Institute of Neurosciences and Physiology, Sahlgrenska Academy at University of Gothenburg, Gothenburg, Sweden

[2]Department of Neurology, University of Helsinki and Helsinki University Hospital, Finland

[3]Department of Neurology and Stroke Center, University of Basel and University Hospital Basel, Switzerland

[4]Department of Neurology, Amsterdam University Medical Centers, the Netherlands

[5]Department of Neurology and Center for Stroke Research, Charité-Universitätsmedizin Berlin, Germany

[6]Department of Neurology, University of Lille, France

[7]Department of Neurology, Centre Hospitalier Universitaire Vaudois and University of Lausanne, Switzerland

[8]Department of Neurology, University Hospital Heidelberg, Germany

[9]Department of Neurology, lnselspital, Bern University Hospital, University of Bern, Switzerland

[10]Department of Clinical and Experimental Sciences, Neurology Clinic, University of Brescia, Italy

For peer review only - http://bmjopen.bmj.com/site/about/guidelines.xhtml

[11]IRCCS Istituto di Scienze Neurologiche di Bologna, Department of Neurology and Stroke Center, Maggiore Hospital, Bologna, Italy

[12]Neurology Clinic, Clinical Centre of Serbia, Belgrade, Serbia

[13]Faculty of Medicine, University of Belgrade, Serbia

[14]Department of Neurology, University of Zürich, Switzerland

[15]Department of Neurology, Klinikum der Universität München, Ludwig-Maximilians University, Germany

[16]Department of Neurology, Kantonsspital St. Gallen, Switzerland

[17]Diagnostic and Interventional Neuroradiology, Sahlgrenska University Hospital and Department of Radiology, Institute of clinical sciences, Sahlgrenska Academy, University of Gothenburg, Sweden

[18]Department of Radiology, Helsinki University Hospital, Finland

[19]Department of Neurology, Hadassah-Hebrew University Medical Center, Jerusalem, Israel

[20]Department of Neurosurgery, Hadassah-Hebrew University Medical Center, Jerusalem, Israel

[21]Department of Radiology, Hadassah-Hebrew University Medical Center. Jerusalem, Israel

[22]Department of Neurology, University Medical Center Goettingen, Germany

[23]Department of Neuroradiology, University Medical Center Goettingen, Germany, and Neuroradiology and Stroke Center Basel, University of Basel and University Hospital Basel, Switzerland

[24]Department of Neurology, University Hospitals Bremen-Mitte and Bremen-Ost, Germany

[25]Clinic for Diagnostic and Interventional Neuroradiology, University Hospitals Bremen-Mitte and Bremen-Ost, Germany

[26]Department of Radiology, Areteion University Hospital, National and Kapodistrian University of Athens, Greece

[27]Institute of Diagnostic and Interventional Neuroradiology, lnselspital, Bern University Hospital, University of Bern, Switzerland

[28]USD Stroke Unit and Vascular Neurology, ASST Spedali Civili Brescia, Brescia, Italy

[29]Department of Neuroradiology, Amsterdam University Medical Centers, the Netherlands

For peer review only

For peer review only - http://bmjopen.bmj.com/site/about/guidelines.xhtml

[30]Institute of Neuroradiology, Charité-Universitätsmedizin Berlin, Germany

[31]Clinic of Neurosurgery, Clinical Centre of Serbia, Belgrade, Serbia

[32]Clinic for Vascular and Endovascular Surgery, Clinical Centre of Serbia, Belgrade, Serbia

[33]Department of Radiology and Nuclear Medicine, Kantonsspital St. Gallen, Switzerland

[34]Department of Neuroradiology, University Hospital Zürich, Switzerland

[35]University of Heidelberg, Neurology Clinic, Department of Neuroradiology, Germany

[36]Department of Internal Medicine, Faculty of Medicine, School of Health Sciences, University of Thessaly, Larissa, Greece

[37] Department of Radiology, University Hospital of Larissa, School of Medicine, University of Thessaly, Larissa, Greece

Corresponding author: Annika Nordanstig, MD, PhD, Department of Neurology, Sahlgrenska University Hospital, Gröna Stråket 11, 3rd floor, 41345 Gothenburg, Sweden
E-mail: annika.nordanstig@vgregion.se ; Tel: + 46 31 3429061, Fax: + 46 31 823650

Authors' e-mail addresses: annika.nordanstig@vgregion.se; sami.curtze@hus.fi; henrik.gensicke@usb.ch; s.m.zinkstok@amc.uva.nl; hebun.erdur@charite.de; camilla.l.karlsson@vgregion.se; nicolas.martinez-majander@hus.fi; jan.scheitz@charite.de; nicolas.bricout2@chru-lille.fr; didier.leys@univ-lille2.fr; ashraf.eskandari@chuv.ch; christian.hametner@med.uni-heidelberg.de; hakan.sarikaya@insel.ch; urs.fischer@insel.ch; mago1959@hotmail.com; a.zini@ausl.bologna.it; visnja.padjen@hotmail.com; dejana.r.jovanovic@gmail.com; andreas.luft@usz.ch; susanne.wegener@usz.ch; lars.kellert@med.uni-muenchen.de; katharina.feil@med.uni-muenchen.de; georg.kaegi@kssg.ch; christopher.traenka@usb.ch; m.i.baharoglu@amc.uva.nl; jan-erik.karlsson@vgregion.se; gerli.sibolt@hus.fi; alexandros.rentzos@vgregion.se; kimmo.lappalainen@hus.fi; david.seiffge@insel.ch; leker@hadassah.org.il; jcohenns@yahoo.com; gomori@cc.huji.ac.il; alex.brehm@med.uni-goettingen.de; jliman@gwdg.de; marios.psychogios@usb.ch; akastrup06@gmail.com; papanagiotou@me.com; alessandro.pezzini@unibs.it; marcel.arnold@insel.ch; peter.arthur.ringleb@med.uni-heidelberg.de; patrik.michel@chuv.ch; hilde.henon@chru-lille.fr; philippe.lyrer@usb.ch; christian.nolte@charite.de; p.j.nederkoorn@amsterdamumc.nl; jan.gralla@insel.ch; c.b.majoie@amsterdamumc.nl; georg.bohner@charite.de; vukasinovic_i@yahoo.co.uk; drvladimircvetic@gmail.com; johannes.weber@kssg.ch; Zsolt.Kulcsar@usz.ch; markus.moehlenbruch@med.uni-heidelberg.de; martin.bendszus@med.uni-heidelberg.de; ntaiosgeorge@gmail.com; gntaios@med.uth.gr; ekapsal@med.uth.gr; katarina.jood@neuro.gu.se; stefan.engelter@usb.ch; daniel.strbian@hus.fi; turgut.tatlisumak@neuro.gu.se

For peer review only

For peer review only - http://bmjopen.bmj.com/site/about/guidelines.xhtml

Running title: EVA-TRISP Registry Methodology
Number of words in manuscript body: 5929
Number of words in abstract: 310
Number of tables: 2
Number of figures: 1 (EVA-TRISP centers)
Number of references: 57
Number of appendices: 3
Appendix 1: EVA-TRISP Investigators (in alphabetical order by country)
Appendix 2: EVA-TRISP database items
Appendix 3: Names of the ethics committees

For peer review only

For peer review only - http://bmjopen.bmj.com/site/about/guidelines.xhtml

ABSTRACT

Purpose

The Thrombolysis in Ischemic Stroke Patients (TRISP) collaboration was a concerted effort initiated 2010 with the purpose to address relevant research questions about the effectiveness and safety of intravenous thrombolysis (IVT). The collaboration also aims to prospectively collect data on patients undergoing endovascular treatment (EVT) and hence the name of the collaboration was changed from TRISP to EVA-TRISP. The methodology of the former TRISP registry for patients treated with IVT has already been published. This paper focuses on describing the EVT part of the registry.

Participants

All centers committed to collecting predefined variables on consecutive patients prospectively. We aim for accuracy and completeness of the data, and to adapt local databases to investigate novel research questions.

Herein, we introduce the methodology of a recently constructed academic investigator-initiated open collaboration EVT registry built as an extension of an existing IVT registry in patients with acute ischemic stroke (AIS).

Findings to date

Currently, the EVA-TRISP network includes 20 stroke centers with considerable expertise in EVT and maintenance of high-quality hospital-based registries.

Following several successful randomized controlled trials (RCTs), many important clinical questions remain unanswered in the (EVT) field and some of them will unlikely be investigated

For peer review only

For peer review only - http://bmjopen.bmj.com/site/about/guidelines.xhtml

in future RCTs. Prospective registries with high-quality data on EVT-treated patients may help answering some of these unanswered issues, especially on safety and efficacy of EVT in specific patient subgroups.

Future plans

This collaborative effort aims at addressing clinically important questions on safety and efficacy of EVT in conditions not covered by RCTs. The TRISP registry generated substantial novel data supporting stroke physicians in their daily decision-making considering IVT candidate patients. While providing observational data on EVT in daily clinical practice, our future findings may likewise be hypothesis-generating for future research as well as for quality improvement (on EVT). The collaboration welcomes participation of further centers willing to fulfill the commitment and the outlined requirements.

Key words

acute ischemic stroke, benchmarking, collaboration, endovascular treatment, large-artery occlusion, outcome, quality, recanalization, registry, stroke, thrombectomy, thrombolysis

For peer review only - http://bmjopen.bmj.com/site/about/guidelines.xhtml

Strengths and limitations of this study

- The EVA-TRISP collaboration offers a platform to pool individual patient data from prospective registries of patients with ischemic stroke undergoing revascularization therapies.
- The large sample size (currently >13 000 EVTs from 20 centers), high level of completeness of data and standardized data ascertainment are strengths of EVA-TRISP.
- EVA-TRISP will provide data from everyday clinical practice and address clinically important questions about safety and outcomes of patients with ischemic stroke treated with EVT who are not covered by randomized controlled trials.
- Data is derived from registries that are neither monitored nor randomized. There will be no control group without EVT which disallows the assessment of effectiveness of EVT in study populations.

INTRODUCTION

Timely recanalization improves outcomes in patients with AIS [1, 2]. Safety and efficacy of recanalization strategies, namely IVT and more recently EVT (including mechanical thrombectomy with various techniques and devices in AIS patients with anterior circulation large-artery occlusions), have been well-documented in several randomized controlled trials (RCTs)[3-9]. A meta-analysis of five RCTs revealed an average 2.5-fold reduction in disability through EVT in large-vessel occlusions compared with standard care, including IVT[10]. Early

For peer review only - http://bmjopen.bmj.com/site/about/guidelines.xhtml

recanalization is currently the cornerstone of acute stroke treatment with increasing use globally. This benefit is substantially higher with earlier achievement of recanalization and diminishes with longer onset-to-treatment intervals[2, 11, 12]. Previous research has explicitly shown that IVT with alteplase within 4.5 hours of symptom onset improved AIS patient outcomes[13].

Following the results of these RCTs, EVT is recommended as standard of care in patients with intracranial large vessel occlusion in several guidelines[14-16]. Consequently, health systems all over the world have adapted and identify and quickly transfer eligible patients to centers offering EVT. Simultaneously, capacity, logistics, know-how and 24/7 coverage were developed to cope with the quickly increasing demand for this intervention. Moreover, two recent RCTs showed benefit with EVT in patients treated up to 16 or 24 hours after stroke onset, given that presence of a considerable amount of salvageable brain tissue had been demonstrated with appropriate imaging methods [17, 18].

Relevant questions in the daily clinical work of treating stroke patients suffering from large vessel occlusions remain. Firstly, EVT-RCTs included a highly selective patient population. This increased the chances to demonstrate efficacy and to exclude patients who presumably had a low chance for a favorable outcome and patients who had a high risk for serious complications. Secondly, the seven published EVT trials[3, 5-9, 19] analyzed altogether only included 1754 randomized patients (of whom 869 underwent EVT) with the single smallest trial including only 65 patients (of whom 33 underwent EVT)[8]. Usually, after a novel treatment is proved effective, a new wave of assumptions and extrapolations for treating a broader domain of patients begins. As all these excluded patient subgroups cannot be studied in future RCTs, in most cases judgments for treating or not treating with EVT will be based on limited knowledge. Some remaining questions will eventually be answered with a long delay, but some will never be answered in forthcoming RCTs. However, stroke physicians keep facing patients where evidence-based data

For peer review only - http://bmjopen.bmj.com/site/about/guidelines.xhtml

do not explicitly contribute to decision-making for these individuals, in which available treatments may very well have a potential benefit as well. Here, prospective high-quality multicenter registries including large numbers of patients representing many subgroups, not included or not separately analyzed within RCT settings, may offer helpful information for basing clinical judgments while being aware that the level of certainty will not reach that gained from RCTs. These registries may also give strong clues on how trial results are implemented to clinical practice and how daily practice safety and efficacy levels match with those gained in RCTs. Further, registry-based data deliver hints in generating new and adequate hypotheses for future RCTs. Another important aspect is the recently developing new field of clot property-research: interested centers can collect detached clots and ship them to laboratories where macroscopic and microscopic properties of the clot coupled with clinical data can be further investigated and may open new avenues in understanding stroke mechanisms. Lastly, quality is a central indicator in health care and registry-based data can be utilized in comparisons and for improving individual center acute stroke care pathways. As a prerequisite, such data have to be based on well-maintained registries containing a large number of detailed, clearly-defined, and well-characterized variables. The EVA-TRISP registry aims at meeting these prerequisites. Utilizing our decade-long experience from the multinational TRISP registry[20], we are now aiming to build a prospective multinational registry of AIS patients treated with EVT including detailed clinical, laboratory, and imaging data for future analyses. We are presenting herein the current versions of the clinical and imaging database items of the EVA-TRISP registry. Additionally, we will discuss a selection of specific related topics.

For peer review only - http://bmjopen.bmj.com/site/about/guidelines.xhtml

AIMS OF THE EVA-TRISP REGISTRY

The major aim of EVA-TRISP is to address clinically important questions about safety and outcomes in AIS patients treated with IVT and/or EVT that are not covered by RCTs. The idea of EVA-TRISP is that experienced stroke centers with a record and expertise in both (I) usage of IVT and/or EVT and (II) maintenance of hospital-based stroke databases pool their data. In addition to the characteristics of the EVA-TRISP centers stated above, an advantage of EVA-TRISP is the availability of more additional variables than in other large-scale registries and the commitment by the collaborators to I) submit accurate and complete data and to II) be willing to adapt the local databases and quickly add new variables retro- and prospectively.

COHORT DESCRIPTION

The Thrombolysis in Ischemic Stroke Patients (TRISP) collaboration was a concerted effort initiated in 2010 by 11 European stroke centers with the purpose to address clinically relevant research questions about the effectiveness and safety of IVT, and currently 20 stroke centers from nine different countries participate in the collaboration (see Figure 1 and Appendix 1 for a list of member sites and investigators). As the collaboration also aims to prospectively collect granular and high-quality data on all consecutive stroke patients undergoing EVT, the name is changed from TRISP to EVA-TRISP.

EVA-TRISP (former TRISP) operates as an independent, non-profit, investigator-driven open platform that focuses on generating high quality data for clinical research purposes. The EVA-TRISP research initiatives are characterized by informal, project-driven and relaxed work processes based on a high level of mutual trust and understanding between participating collaborators, and no formal scientific leadership committees have so far been implemented or

For peer review only - http://bmjopen.bmj.com/site/about/guidelines.xhtml

deemed necessary. Internal communication principally takes place via e-mail and teleconferences. The EVA-TRISP collaboration also hosts an annual face-to-face meeting during the European Stroke Organisation (ESO) conference, and this forum is utilized for overall strategic planning and major decisions. The collaboration welcomes new collaborators and project proposals from all stroke centers that fulfill the requirements as stated below. The collaboration particularly aims at supporting young researchers. Thus, with the exception of the very first paper[21], first authors of the publications generated by the TRISP invariably have been young stroke physicians or PhD-students. The methodology of the TRISP registry has previously been published[20], and currently data on more than 18,000 IVT-treated patients are available in the registry. This paper focuses on describing the EVT part of the registry.

The EVA-TRISP registry aims to prospectively collect granular and high-quality data on all consecutive AIS patients undergoing both IVT and/or EVT. The overall purpose is to provide a means to address clinically important research questions on the safety and effectiveness of IVT and/or EVT in AIS patients that are typically not covered by RCTs or single center research initiatives. Furthermore, the EVA-TRISP aims at providing a large data source for various stroke care quality improvement initiatives. All EVA-TRISP centers have a proven track-record for delivering high-quality and high-volume stroke patient care. Every EVA-TRISP center offers stroke management that fulfills the criteria of Stroke Centers or Stroke Units as proposed by the ESO[22]. The simple idea of the EVT part of the EVA-TRISP registry is that experienced stroke centers with expertise in both EVT implementation and in the maintenance of hospital-based EVT databases pool their data together. An advantage of the EVA-TRISP registry is the availability of substantially more variables than in other large-scale registries. We strive for a commitment by the collaborators to provide data of high accuracy and completeness as well as towards a willingness among collaborators to swiftly adapt the local databases by adding new

For peer review only - http://bmjopen.bmj.com/site/about/guidelines.xhtml

variables of interest. This enables a potential for explorative insights in the putative prognostic importance of variables with unknown influence on outcome or risk of complications, such as symptomatic intracranial hemorrhage (sICH). Strengths and limitations of the EVA-TRISP registry in general and compared to other existing EVT registries are discussed below (please see Discussion).

The participating centers have all agreed to fulfill the prerequisites that are summarized in Table 1. Participation in other registries does not preclude participation in the EVA-TRISP registry. A standard database template has been developed by an international expert group consisting of stroke physicians and scientists in collaboration with all members, leading to the current standard version of the registry (see Appendix 2-Registry data elements) which has been agreed upon by all EVA-TRISP member sites. This comprehensive dataset includes over 110 items and covers a wide range of data elements, including demographics, pre-stroke health information, acute phase management, and long-term outcomes including three months and one-year modified Rankin Scale (mRS) as well as detailed laboratory data and imaging findings. An add-on imaging repository is currently under preparation and will be implemented within the near future. Electronic medical records used at all member sites allow for additional variables to be collected quickly and reliably when deemed necessary for new projects.

Target population

All AIS patients designated for EVT and in whom interventionalists gained arterial access are within the target population. Therefore, this registry also may include patients with misdiagnosis, already recanalized leading to premature interruption of the procedure, unsuccessful attempts to recanalize, and other unforeseeable conditions. Inclusion of patients is not limited to certain EVT

For peer review only - http://bmjopen.bmj.com/site/about/guidelines.xhtml

techniques. The registry will include also patients who receive intra-arterial thrombolysis even without mechanical thrombectomy.

Data collection and definitions

Data on the characteristics of patients are collected prospectively by all participating centers using standardized definitions and a standardized form. Not all centers have to provide data on all variables but have given a commitment to add missing variables retrospectively, if considered relevant to answer a specific research question.

The dataset includes patient demographics, history, prehospital information, admission data, details on acute interventions, stroke unit and/or intensive care information, discharge, rehabilitation, outcome data (three months and one-year outcomes measured by mRS), as well as detailed laboratory test results, vital signs, and imaging findings (see Appendix 2). Risk factors and stroke etiology will be determined according to standard approaches across centers[20].

Moreover, neuroimaging findings before and after treatment are systematically ascertained and comprise imaging modality (computed tomography versus magnetic resonance imaging) as well as specific imaging findings such as hyperdense artery sign, presence and extent of early ischemic signs, site of vessel occlusion, collateral status, presence of tandem occlusion of ipsilateral carotid artery, recanalization status immediately after EVT and on follow-up imaging (quantified according to modified treatment in cerebral ischemia (mTICI) score)[23], white matter disease severity, presence and burden of cerebral microbleeds.

All patients are monitored for occurrence of hemorrhagic transformation. Follow-up imaging usually takes place close to 24 hours after treatment or earlier in case of clinical worsening. Some centers perform follow-up imaging only in cases with clinical worsening. The definition of

For peer review only - http://bmjopen.bmj.com/site/about/guidelines.xhtml

symptomatic intracerebral hemorrhage (sICH) is in accordance with the definition used in the European Cooperative Acute Stroke Study II ("an intracranial hemorrhage was defined as symptomatic if the patient had clinical deterioration causing an increase in the National Institute of Health Stroke Scale (NIHSS) score of more than or equal to four points and if the hemorrhage was likely to be the cause of the clinical deterioration")[24]. The majority of centers additionally evaluate type of hemorrhagic transformation (hemorrhagic infarction, parenchymal hemorrhage), indicate whether the bleeding occurred remotely from the infarcted area, and document sICH according to definitions used in the National Institute of Neurological Disorders and Stroke Trial I+II, SITS-MOST and ECASS III definitions[25, 26, 27]. Functional outcomes at three months and one-year are assessed using the mRS. The mRS is obtained by telephone calls, postal/electronical questionnaires, or outpatient visits. If patients cannot be interviewed, close relatives, nurses or family doctors are asked for disability status.

We are currently exploring technical, legal, and ethical as well as financial backgrounds for establishing an electronic registry to one of the member centers that would allow direct data insertion from each center and holding the registry compactly in one single file. Otherwise, each center will maintain their own registry within their own electronic system and data will always be transferred without personal identifiers for each single analysis looking at one single aspect and leading to one mutual international publication. Establishing and maintaining such a large database with complete compliance to rules and safety measures is a costly procedure and requires long-term funding. This option is now under exploration.

Future plans in the registry include the addition of a neuroradiology imaging bank that will enable detailed image analysis of patients treated with IVT and/or EVT. The imaging bank will provide "real-world" diagnostic neuroradiology and, used in combination with detailed clinical information from the EVA-TRISP registry, analysis of this imaging data will help to (i) create

For peer review only - http://bmjopen.bmj.com/site/about/guidelines.xhtml

standardized imaging protocols in acute stroke (i.e. defining optimal threshold for perfusion parameters), (ii) identify new (i.e. a collateral score for the posterior circulation) and validate published (i.e. different collateral scores for the anterior circulation) imaging outcome predictors and imaging-based selection tools for reperfusion therapies, (iii) assess the generalizability of RCT results to subgroups of patients who would have been excluded based on imaging criteria (i.e. baseline Alberta Stroke Program Early CT score [ASPECTS] under five, extracranial vessel pathologies), (iv) improve automated analyzing techniques (i.e. machine and deep learning algorithms), (v) and enhance the accuracy of outcome prediction of different clinical and imaging parameters by implementing new imaging outcomes (f.i. infarct volume, recanalization status).

Neuroimages from TRISP/EVA-TRISP patients since 2015 will be pooled centrally. All imaging modalities (non-contrast CT, CT-angiography, CT-perfusion, MRI, MRA, MR-perfusion, and digital subtraction angiography) at baseline and follow-up (up to three months after stroke onset) will be eligible for analysis. Image analyses will be performed blinded to clinical information and treatment decisions, and undergo a central systematic re-evaluation using a specified case report form including all predefined imaging variables.

Stroke-specific image analyzing software (i.e. OSIRIX medical imaging viewer, Quantomo for semi-automated volumetric analysis, and Rapid Processing of Perfusion and Diffusion [RAPID] for perfusion analysis) could be utilized. A detailed case report form for image analysis will be designed in review with all collaborators.

Lastly, EVA-TRISP investigators prepared a detailed standard operating procedure (SOP) to ascertain that all members collecting data are well-aware of standard interpretations and follow identical steps to avoid unnecessary heterogeneities or individual-borne differences. The numbers of recruited patients in each participating center during the study period until the end of year 2019 are reported in Table 2 along with the population each center is covering for EVT.

For peer review only - http://bmjopen.bmj.com/site/about/guidelines.xhtml

Data elements and completeness

A detailed database is aimed to facilitate the investigation of various current and future topics. Data elements are listed in detail in Appendix 2. Over time, new data elements may become necessary for new individual projects. Should this occur, investigators will quickly supplement the missing variables. Patient age, sex, admission NIHSS score, recanalization status before and after thrombectomy, and three months outcome measured by mRS are obligatory data items and must be present for all patients (otherwise a patient is not eligible to be included to the registry). In general, missing data for any variable or patient must not exceed 10%.

Data sources

All paper-based or electronic patient files including laboratory values and imaging data will be utilized to capture the EVA-TRISP registry data points. Based on these different sources alongside repeated clinical evaluations undertaken by the dedicated EVA-TRISP collaborator, registry data points that initially remain missing during the early stroke treatment process will in the majority of cases be possible to reconstruct and thereafter reported to the local registry (e.g. NIHSS scores). In most cases, the local EVA-TRISP investigators form the local stroke team and will be actively seeing the patients already in the emergency room and/or at their own stroke units and can therefore guarantee completeness of data in most cases.

Quality control

Quality control is another crucial step in multicenter large-sized registries since missing data are a frequent problem impairing the reliability and generalizability of registry-borne data. The EVA-TRISP registry is different in this sense because data are not yet directly collected to a central

For peer review only - http://bmjopen.bmj.com/site/about/guidelines.xhtml

registry, but each center collects their own data to their own institutional registry according to a standard harmonized database item list and SOP. Thus, clear variable definitions are easily available for the user when data are entered. It is the responsibility of the local EVA-TRISP collaborator (usually a senior stroke physician) to check and account for the validity and completeness of all data points introduced to the local registry. Therefore, missing data are expected to be very low. Furthermore, all centers have agreed to include all consecutive patients attempted with an EVT and all centers will undertake frequent spot checks against internal hospital administrative systems covering EVT procedures, as to not leave any patient out of the registry. Therefore our registry data will likely include all EVTs performed within a region and population, practically equaling to a population-based study, although being hospital-based, because EVT is usually available only at stroke centers serving a predefined region and the inhabitant population. Most of the required data come from routine procedures which are standardly collected and recorded in stroke patient care pathways as part of the clinical routine and therefore these data points are almost always retrievable. In the subsequent quality control process, the data files from the individual participating centers are merged into a single file for further maintenance analyses. Pseudonymized individual center data are sent to the center leading the specific project using encrypted transfer protocols. The subsequent data management of the merged database will implement checks for missing data along with checks for range, consistency and illogical data (e.g. NIHSS score cannot be minus or over 42 points and can only be full points and not decimals; patient age at stroke onset can be only in digits, and is expected to be from 16 and very rarely over 100). Also other procedures regarding quality check will be implemented at milestone points, such as comparing the performance of data reporting among centers.

For peer review only - http://bmjopen.bmj.com/site/about/guidelines.xhtml

Registry size and duration

The registry will include all EVT patients from all member sites. We anticipate that the absolute numbers and proportions of EVT-treated AIS patients will be increasing over time and annual inserts will exceed thousands shortly. The registry size is not limited. One anticipated strength of this registry is the high patient number together with detailed information on each patient which will allow us to look at many issues that are not feasible to investigate within RCTs or even merged data from all RCTs because of the fact that they include fairly small patient numbers. The use of the registry will be launched after 5000 patients' data have been inserted and adequately quality-checked. Similarly, this registry will be utilized as long as EVT is a viable option in stroke treatment. The unlimited time span requires careful evaluation by ethics committees. If the consortium decides to end the registry, each center's data will be adequately returned to the owners and the registry data will be deleted achieving an absolutely non-retrievable condition according to technical standard operating procedures (SOPs) of the registry-holding center. Thereafter, each individual center will be free to decide how to proceed with their own datasets. Similarly, if a center decides to resign from the registry, their data will be adequately returned and will be deleted from the main database file after confirmation that the data are safely received by the local principal investigator. While all patient-related data including clinical, laboratory, and imaging data are completely anonymized, each center will keep a key file within their local electronic hospital system with patient identifiers matching to the patient code in the registry (e.g. if necessary to go back to patient files). This approach is compliant with current principles and is the SOP worldwide.

For peer review only

Lead of a single project and authorship principles

For peer review only - http://bmjopen.bmj.com/site/about/guidelines.xhtml

The researcher or researchers - usually one or two (rarely three) - who originally present the idea and render the analysis proposal make(s) the initiation by drafting a standard 1-2 pages draft stating a clear hypothesis and statistical plan summarizing the project (project proposal). The proposal is circulated to all member centers and discussed for scientific content and feasibility enriched with input from a large expert community. After that, the enriched plan along with the list of data items required is recirculated. If a center agrees to participate, the center contributes data within the in-advance agreed time frame on all consecutive patients with the key variables of interest. For some projects retrospective collection of data is required. The original proposal makers are entitled to the first and senior authorships. Co-authorships are distributed according to contributions. This includes not only mere quantitative means (i.e. number of patients contributed) but also quality of data (e.g. completeness; considered high across EVA-TRISP centers), handling and pooling of the multicenter data; maintenance of the pooled data set (including data cleaning), statistics, contribution to EVA-TRISP in general, and intellectual input in details of the design or the analyses of the research project, and lastly intellectual input to the writing and improving of the manuscript. These criteria are suggestions and the researches taking the lead in each project take the final responsibility for the fair distribution of authorships. Each member site possesses its own data and each member site whose data is utilized is entitled to co-authorship(s). Whenever feasible, an abstract approved by all co-authors is submitted to the forthcoming ESO Conference. All EVA-TRISP member centers and investigators are listed at the end of the manuscript as a supplement given that the publishing journal's own format allows this approach.

Data ownership, access, use, and publications

For peer review only

For peer review only - http://bmjopen.bmj.com/site/about/guidelines.xhtml

Each center is self-financing in data collection and is the indisputable owner of their own data. If and when a mutual single databank is constructed, each individual researcher with the necessary formal training and permissions will be able to insert data directly to the central database on the internet and have access to the researcher's own center's data without limitations. Individual scientists working on a properly agreed upon single project and doing data analyses will be granted proper access to all data. In case that an individual center refrains from participating in a particular analysis, their data will not be included in that analysis. Any publication that is produced from the registry data will include authors from each contributing center in accordance to number of patients delivered as well as active involvement in analyses and writing work. Number of authors and their placement in the author list may vary according to the amount of contributions. This will be handled openly and in a delicate way aiming at mutual consent.

Ethics, informed consent, and privacy

Each center has received necessary official approval from their respective local authorities and/or ethical committees according to their national and local rules (Appendix 3). These permits include transfer of data between EVA-TRISP centers. Necessity of individual informed consent is dependent on national rules and will be collected if necessary. Data are shared with respect to the EU law 2016/679 about General Data Protection Regulation (GDPR). In the long run, the aim is to have a permanent database residing at a member site. Establishment and maintenance of the permanent database at one center will be initiated only after a separate ethics approval.

DISCUSSION

For peer review only

For peer review only - http://bmjopen.bmj.com/site/about/guidelines.xhtml

Endovascular treatment, now fulfilling the criteria for the highest level of evidence, has changed acute stroke care substantially. Probably approximately 10% of all ischemic stroke patients are eligible for EVT, but the percentages may grow as more and more patients are brought to the attention of emergency systems and the treatment indications will likely expand over time[28]. The rapid developments in acute stroke care put considerable demands on health care systems and necessitate quick rearrangements for coupling these needs. The seven published RCTs and following meta-analyses answered most central questions. Nevertheless, there are numerous unanswered questions remaining in terms of EVT in acute ischemic stroke. Some of these questions will be solved and satisfied via ongoing and forthcoming RCTs. Yet, many other issues will never or unlikely be tested in RCTs and stroke physicians need firm data on these topics to base their clinical decisions on. Moreover, there are certain patient groups where RCTs are ethically difficult to organize; such a group is patients with basilar artery occlusion (BAO). Although a clearly important clinical condition which untreated has a poor outcome, BAO patients were not included in the large EVT trials. An extrapolation from the anterior circulation EVT trial results for BAO has currently a strong support in clinical practice, and thus EVT is currently offered to BAO patients despite limited direct evidence of treatment effectiveness. A small multi-center RCT that included 131 patients– the Chinese BEST trial (Basilar artery occlusion Endovascular intervention versus Standard medical Treatment)[29]– was prematurely terminated due to slow recruitment and a high crossover rate that severely hampered the interpretability of the intent-to-treat analysis. Another RCT with 300 patients included is closed, but data has not yet been presented[30]. Still, both the per-protocol and the as-treated analyses favored EVT compared to best medical treatment. Some small-sized registry data showed high recanalization rates and similar hemorrhagic complication rates as in anterior circulation patients

For peer review only - http://bmjopen.bmj.com/site/about/guidelines.xhtml

treated with EVT, but more often futile recanalization. Large-scale registry studies may further improve our knowledge in this patient group and may help identifying those who will likely benefit or not benefit from EVT in a real-life setting[6, 31-33]. In the absence of RCT-based data, comprehensive observational data may be useful for individual treatment decisions in clinical practice and in evaluating processes of stroke triage and care for IVT or EVT. As a prerequisite, such data have to be based on well-maintained registries containing large numbers of detailed, clearly-defined, and well-characterized variables. The EVA-TRISP registry meets these prerequisites. Ideally, the results from such observational studies are verified or falsified by RCTs. However, with few exceptions (e.g. age limit) this is unlikely to happen. Thus, registry-based data will reflect the highest level of evidence in several aspects, available currently and in the foreseeable future.

Further, we need to continuously follow-up whether the safety and benefit aspects of EVT shown in RCTs could be correctly translated to routine clinical practice. Indeed, the safety and benefit may be better, similar, or even worse in daily practice. Registry data can easily be compared to RCT data especially when basic settings are similar. Additionally, it becomes more and more feasible to compare centers, patient subgroups and devices. Benchmarking, previously performed by site visits, is a popular approach for understanding differences and making improvements, and can now be done easily using electronic data[34].

Systematically ascertained, comprehensive and high-quality observational data are useful to both (I) challenge or (II) confirm the clinical usefulness of commonly used but often arbitrary eligibility criteria. An early example has been the challenge of the usefulness of the upper age limit of 80 years for IVT based on comprehensive, observational studies. Eventually, the third International Stroke Trial (IST-3) proved that indeed patients aged 80 years and older benefit from IVT, too[35].

For peer review only - http://bmjopen.bmj.com/site/about/guidelines.xhtml
For peer review only

Utilizing the TRISP registry, we previously examined the safety of IVT in a number of patient subgroups where RCT-based data did not exist. Previous publications of the TRISP registry (I) provided insight into safety and efficacy of IVT in subgroups of patients who were excluded in RCTs (e.g. patients dependent on the help of others prior to stroke), underrepresented or not specifically addressed (e.g. dissection as cause, impaired renal function, low platelet count, body-mass-index, prior use of statins, serotonin uptake inhibitors, prior use of novel oral anticoagulants, patients with seizure at onset)[21, 36-45], (II) facilitated the evaluation process of acute stroke care such as the meaning of the “off-hour-thrombolysis”, IVT during “working hours”, or the variable “time” in clinical practice[46-48], and (III) served to derive, validate, and compare risk scores for sICH or functional three months outcome[49-51]. These registry-based novel data contributed to the numbers of patients treated safely and successfully with IVT globally. Ongoing and planned research projects within the EVA-TRISP registry collaboration that may fill important knowledge gaps are investigations on (I) stroke due to cervical artery dissection, (II) stroke with low baseline NIHSS, (III) stroke specifically in the ACA-territory, (IV) stroke patients with preexisting dependency, (V) significance of cerebral collaterals, (VI) significance of tandem occlusions and (VII) stroke patients with active cancer.

For peer review only

Disease- or intervention-based patient registries with consecutive patients recruited in a population- or hospital-based approach are useful in many ways: they help describe the natural history, determine clinical effectiveness and cost-effectiveness of health care products or services, measure or monitor safety and harm, measure quality of care, improve quality of care, and help with benchmarking purposes such as how clinical practices vary, what the best predictors of treatment practices are, and comparing different practices providing a basis for further improvements. In such settings, stakeholders are several: the primary stakeholder with the

For peer review only - http://bmjopen.bmj.com/site/about/guidelines.xhtml

EVA-TRISP registry is the academic consortium establishing and running the registry. Potential stakeholders with such a large-scale registry may include public health and regulatory authorities, product manufacturers, health care service providers, payer and commissioning authorities, patients and their advocacy groups, treating physician groups, academic institutions, and professional societies. The EVA-TRISP registry aims at including all patients who underwent EVT as a treatment for AIS, including patients with misdiagnosis, unsuccessful attempts (EVT is defined as a puncture to the artery with the aim of recanalization), or other unforeseeable scenarios. In most countries, registry-based studies are approved by ethics committees with waving informed consent from individual patients as demanding informed consent would leave most severe patients out of the registry and cause a severe bias on representability of any finding. Imaging has become more and more critical in the stroke field. In addition to stroke diagnostics and excluding competing etiologies (e.g. stroke mimics), there are a number of imaging findings related to increased risk following acute treatments (e.g. leukoaraiosis[52] and microbleeds), or findings guiding treatment choices (e.g. major artery occlusion reachable with a catheter), as well as findings helpful in prognostics such as ASPECTS[53] or SEDAN[49] scores. Acute stroke patients are increasingly imaged with a package of standard CT, CT angiography and CT perfusion or in a similar fashion with an MRI-based package. These imaging modalities are then analyzed quickly for determining diagnosis, prognosis, and treatment approach. Functional imaging modalities are increasingly taken into account for patient selection to IVT and EVT instead of strictly deciding according to time. The former TRISP registry included few items on imaging studies. Imaging requirements were according to routine thrombolysis: only a non-contrast CT imaging prior to thrombolysis was mandatory. Imaging was not the main focus as image-analysis is usually labor-intense and not always available. Technological improvements may substitute some of the expert workforce in image analysis already today and in the near future. However, with the

For peer review only - http://bmjopen.bmj.com/site/about/guidelines.xhtml

616 developments in imaging technologies and increased requirements in patient care, currently most
617 patients are imaged with CT angiography and CT-perfusion in addition to basic non-contract CT
618 imaging. Less frequently, patients are imaged with a similar versatile package of various MRI
619 sequences. Developments in the imaging technology and logistics, decrease in radiation dose
620 used with CT imaging, as well as automatic image-analysis software development contributed to
621 the progress. Installing imaging scanners into or adjacent to emergency departments or taking
622 acute stroke suspect patients directly to the imaging facility by-passing the emergency room have
623 also improved the availability of more detailed imaging. Patient selection for the best individual
624 treatment is becoming increasingly dependent on neuroimaging with the goal of providing rapid
625 patient-specific metrics such as tissue viability, vessel patency status, thrombus characteristics
626 and cerebral perfusion etc. Imaging findings have also been used for patient selection in some
627 highly successful IVT and EVT RCTs[4, 5, 54-56]. Detailed imaging information is even more crucial
628 when EVT is considered. Therefore, the establishing of an imaging repository parallel with the
629 EVA-TRISP registry received a widespread support from members. To date, the choice of
630 imaging modalities, parameters and thresholds varies widely across medical centers. No
631 standardized imaging protocols currently exist, other than joint statements from professional
632 societies[57]. A large, multicenter neuroimaging registry with state-of-the-art re-evaluation of
633 images combined with detailed clinical data of IVT/EVT-treated stroke patients would be helpful
634 for validating between modalities, defining thresholds, enhancing automated assessments and
635 creating standards in neuroimaging for acute ischemic stroke. For the imaging part of the
636 database we will collect baseline, interventional and follow-up images (up to three months after
637 stroke onset) from all stroke patients included in TRISP since 2012. All images will be centrally
638 analyzed using a predefined, standardized form.

639

For peer review only

For peer review only - http://bmjopen.bmj.com/site/about/guidelines.xhtml

Strengths and limitations of the EVA-TRISP registry

Strengths of EVA-TRISP registry include (I) the high completeness level of data with few missing data, (II) large sample sizes which reduce the risk of bias and allows adjustments for confounders, (III) the systematic and standardized data ascertainment which increases the homogeneity of the study population, (IV) the intrinsic motivation of the study personnel which leads to a high rate of completeness of ascertained data sets, contributing to a high-quality registry, and (V) the dynamic nature of the EVA-TRISP database due to the commitment of the centers to adapt the local database and add variables retro- and prospectively. In addition, (VI) a large number of variables is gathered including those with unknown prognostic importance. This allows addressing novel yet unidentified research questions. Moreover, (VII) pooling of individual patient data increases generalizability compared to single center studies, and (VIII) the fact that variables and outcomes have been collected irrespective of the present research question, reduces the risk of a bias. (IX) As most EVA-TRISP centers are regional reference centers for acute patient care, particularly for EVT, the EVA-TRISP registry will resemble a population-based registry. Limitations are inherent to the design of EVA-TRISP: (I) Data is derived from registries that are neither monitored nor randomized. Usually, there will be no control group without EVT which disallows the assessment of effectiveness of EVT in study populations. (II) As true for all observational studies, analyses based on registers have a higher risk of bias than RCTs. Thus, we urge to a cautious interpretation of findings and observations. (III) All EVA-TRISP centers are experienced in stroke treatment. This expertise implies – as a downside – a limited generalizability of findings to all stroke providers with less expertise and less advanced settings. (IV) The majority of our included patients are Caucasians and from high-income countries. Thus, we cannot compare ethnical differences, nor can we compare health systems with various funding levels. (V) Currently, there is no 'core lab' to validate hemorrhagic

For peer review only - http://bmjopen.bmj.com/site/about/guidelines.xhtml

complications and three-month mRS ratings. As valid for other major registries like SITS and GWTG, local interpretation of outcome data may differ between sites. Since EVA-TRISP centers are mostly high-volume centers with long-standing experience in maintaining IVT databases, this bias is likely to be smaller than in most of the other registries.

SUMMARY

The EVA-TRISP collaboration is an open platform dedicated to conduct joint research projects in AIS patients treated with IVT and/or EVT. EVA-TRISP aims to increase knowledge on the safety and efficacy of IVT and EVT, study outcomes after IVT and EVT, to evaluate processes of acute stroke care as well as to document and improve acute stroke care quality. Our previous achievements prove that this collaboration has the potential to provide versatile observational information on treatment of AIS patients during daily clinical practice. Prospective and standardized documentation of individual patient data according to consensus definitions is a major requirement to maintain the quality of the EVA-TRISP registry. Publishing this methodology paper improves the transparency of the registry and collected data. EVA-TRISP welcomes participation and project proposals of further centers fulfilling the requirements stated above.

Consent for publication

Not applicable.

Availability of data and materials

For peer review only - http://bmjopen.bmj.com/site/about/guidelines.xhtml

Not applicable.

Competing interests

The authors declare that they have no competing interests.

Patient and public involvement

Patients or the public were not involved in the design, or conduct, or reporting, or dissemination plans of our research.

Funding

None.

Authors' contributions

Data collection: AN, SC, HG, SMZ, HE, CK, JEK, NMM, GS, PAL, CT, MIB, JFS, NB, HH, DL, AE, PM, CH, PAR, MA, UF, HS, DJS, AP, AZ, VP, DRJ, ARL, SW, LK, KF, GK, AR, KL, RRL, JC, JG, AB, JL, MP, AK, PP, JG, Mauro M, CM, GB, IV, VC, JW, ZK, MB, Markus M, GN, EK, KJ, CHN, PJN, STE, DS, TT. Manuscript drafting: AN, SC, HG, SMZ, HE, CK, CHN, PJN, KJ, STE, DS, TT. Study supervision: TT, DS, STE, KJ, PJN, CHN. Statistical analysis and interpretation: does not apply. Review of the manuscript for intellectual contribution: AN, SC, HG, SMZ, HE, CK, JEK, NMM, GS, PAL, CT, MIB, JFS, NB, HH, DL, AE, PM, CH, PAR, MA, UF, HS, DJS, AP, AZ, VP, DRJ, ARL, SW, LK, KF, GK, AR, KL, RRL, JC, JG, AB, JL, MP, AK, PP, JG, Mauro M, CM, GB, IV, VC, JW, ZK, MB, Markus M, GN, EK, KJ, CHN, PJN, STE, DS, TT . All authors agreed on submitting this last version of the manuscript to the journal.

For peer review only - http://bmjopen.bmj.com/site/about/guidelines.xhtml

Acknowledgments

We thank Ms. Anu Eräkanto and Ms. Judith Klecki for technical support.

Disclosures

TT: Academic grants from the European Union, National Stroke Research Institute of Australia, Sahlgrenska University Hospital, Sigrid Juselius Foundation, University of Gothenburg, and Wennerström Foundation. Study contracts with Bayer, Boehringer Ingelheim, Bristol Myers Squibb, and Portola Pharm. Personal fees from Bayer, Boehringer Ingelheim, Bristol Myers Squibb, Lumosa Pharmaceuticals, and Portola Pharmaceuticals. TT received speaker's fees from the University of Donau, Krems, Austria. He holds patents on a treatment aiming to avoid post-thrombolytic brain edema and intracerebral hemorrhage in stroke (method to prevent brain edema and reperfusion injury; method to prevent post-thrombolytic hemorrhage formation).

STE has received funding for travel or speaker honoraria from Bayer, Boehringer Ingelheim and Daiichi-Sankyo. He has served on scientific advisory boards for Bayer, Boehringer Ingelheim, BMS/Pfizer, and MindMaze and on the editorial board of Stroke. His institutions have received an educational grant from Pfizer, compensation from Stago for educational efforts and research support from Daiichi-Sankyo, the Science Funds [Wissenschaftsfonds] of the University Hospital Basel, the University Basel, the "Freiwillige Akademische Gesellschaft Basel", the Swiss Heart Foundation, and the Swiss National Science Foundation.

PL has received funding for travel or speaker honoraria from Bayer, Boehringer Ingelheim, and has served on scientific advisory boards for Bayer, Boehringer Ingelheim, Pfizer/BMS, Amgen, and Medtronic. His institutions have received research support from Bayer, and Sanofi, the Science Funds [Wissenschaftsfonds] of the University Hospital Basel, the Swiss Heart

For peer review only

For peer review only - http://bmjopen.bmj.com/site/about/guidelines.xhtml

Foundation, and the Swiss National Science Foundation, and the University Hospital Basel foundation “propatient”.

CHN has received funding for travel or speaker honoraria from Bayer, Boehringer Ingelheim, Pfizer Pharma, Bristol-Myers Squibb, Gore and Ass. and Sanofi. His institutions have received research support or grants from the German Federal Ministry of Research and Education (BMBF) namely the “Center for Stroke Research Berlin, CSB”, the “Deutsches Zentrum für Herz-Kreislauf Forschung (DZHK)”, “Deutsches Zentrum für neurodegenerative Erkrankungen (DZNE)”.

SW has received speaker honoraria from Amgen, travel honoraria from Bayer and a research grant from Boehringer Ingelheim and academic grants from the Swiss National Science Foundation, the UZH (Clinical Research Priority Program Stroke), the Swiss Heart foundation and the Olga Mayenfish foundation.

AZ has received speaker fees and consulting fees from Boehringer-Ingelheim, Medtronic, Cerenovus and advisory board from Daiichi Sankyo and Boehringer-Ingelheim and Stryker.

MB is on the editorial board for Springer as Co-editor of "Clinical Neuroradiology". He serves on advisory boards for Boehringer, Vascular Dynamics and BBRaun. He received speaker honoraria from Guerbet, Bayer, Novartis, Codman, Roche, Teva, Grifols and Merck. He received research support from Novartis, Guerbet, Siemens, Hopp foundation, the European Union, and from Deutsche Forschungs Gesellschaft.

MM is consultant for Acandis, Cerenovus, Medtronic, MicroVention, Route92, Stryker. He received research support from Balt, MicroVention, Siemens, Stryker.

PR is on scientific advisory boards for Bayer and Boehringer-Ingelheim not in relation to the submitted manuscript. He received funding for travel or speaker honoraria from SITS, and lecture fees and travel compensation from Boehringer Ingelheim, Bayer, BMS, Pfizer. He is on the

For peer review only - http://bmjopen.bmj.com/site/about/guidelines.xhtml

editorial board of the journal Stroke as Associate editor (since 2010), of the journal Gefäßchirurgie (editor since 2014), and of the ESO-Journal (Editor since 2015). The University of Heidelberg received research support from Boehringer Ingelheim for the ECASS 4 study, and funding from the German research foundation for the SPACE-2 study.

REFERENCES

1. Rha JH and Saver JL. The impact of recanalization on ischemic stroke outcome: a meta-analysis. *Stroke*. 2007;38:967-73.

2. Saver JL, Goyal M, van der Lugt A, Menon BK, Majoie CB, Dippel DW, Campbell BC, Nogueira RG, Demchuk AM, Tomasello A, Cardona P, Devlin TG, Frei DF, du Mesnil de Rochemont R, Berkhemer OA, Jovin TG, Siddiqui AH, van Zwam WH, Davis SM, Castano C, Sapkota BL, Fransen PS, Molina C, van Oostenbrugge RJ, Chamorro A, Lingsma H, Silver FL, Donnan GA, Shuaib A, Brown S, Stouch B, Mitchell PJ, Davalos A, Roos YB, Hill MD and Collaborators H. Time to Treatment With Endovascular Thrombectomy and Outcomes From Ischemic Stroke: A Meta-analysis. *JAMA*. 2016;316:1279-88.

3. Berkhemer OA, Fransen PS, Beumer D, van den Berg LA, Lingsma HF, Yoo AJ, Schonewille WJ, Vos JA, Nederkoorn PJ, Wermer MJ, van Walderveen MA, Staals J, Hofmeijer J, van Oostayen JA, Lycklama a Nijeholt GJ, Boiten J, Brouwer PA, Emmer BJ, de Bruijn SF, van Dijk LC, Kappelle LJ, Lo RH, van Dijk EJ, de Vries J, de Kort PL, van Rooij WJ, van den Berg JS, van Hasselt BA, Aerden LA, Dallinga RJ, Visser MC, Bot JC, Vroomen PC, Eshghi O, Schreuder TH, Heijboer RJ, Keizer K, Tielbeek AV, den Hertog HM, Gerrits DG, van den Berg-Vos RM, Karas GB, Steyerberg EW, Flach HZ, Marquering HA, Sprengers ME, Jenniskens SF, Beenen LF, van den Berg R, Koudstaal PJ, van Zwam WH, Roos YB, van der Lugt A, van Oostenbrugge RJ, Majoie CB, Dippel DW and Investigators MC. A randomized trial of intraarterial treatment for acute ischemic stroke. *N Engl J Med*. 2015;372:11-20.

4. Goyal M, Demchuk AM, Menon BK, Eesa M, Rempel JL, Thornton J, Roy D, Jovin TG, Willinsky RA, Sapkota BL, Dowlatshahi D, Frei DF, Kamal NR, Montanera WJ, Poppe AY, Ryckborst KJ, Silver FL, Shuaib A, Tampieri D, Williams D, Bang OY, Baxter BW, Burns PA, Choe H, Heo JH, Holmstedt CA, Jankowitz B, Kelly M, Linares G, Mandzia JL, Shankar J, Sohn SI, Swartz RH, Barber PA, Coutts SB, Smith EE, Morrish WF, Weill A, Subramaniam S, Mitha AP, Wong JH, Lowerison MW, Sajobi TT, Hill MD and Investigators ET. Randomized assessment of rapid endovascular treatment of ischemic stroke. *N Engl J Med*. 2015;372:1019-30.

5. Campbell BC, Mitchell PJ, Kleinig TJ, Dewey HM, Churilov L, Yassi N, Yan B, Dowling RJ, Parsons MW, Oxley TJ, Wu TY, Brooks M, Simpson MA, Miteff F, Levi CR, Krause M, Harrington TJ, Faulder KC, Steinfort BS, Priglinger M, Ang T, Scroop R, Barber PA, McGuinness B, Wijeratne T, Phan TG, Chong W, Chandra RV, Bladin CF, Badve M, Rice H, de Villiers L, Ma H, Desmond PM, Donnan GA, Davis SM and Investigators E-I. Endovascular therapy for ischemic stroke with perfusion-imaging selection. *N Engl J Med*. 2015;372:1009-18.

6. Saver JL, Goyal M, Bonafe A, Diener HC, Levy EI, Pereira VM, Albers GW, Cognard C, Cohen DJ, Hacke W, Jansen O, Jovin TG, Mattle HP, Nogueira RG, Siddiqui AH, Yavagal DR, Baxter BW, Devlin TG, Lopes DK, Reddy VK, du Mesnil de Rochemont R, Singer OC, Jahan R and Investigators SP. Stent-retriever thrombectomy after intravenous t-PA vs. t-PA alone in stroke. *N Engl J Med*. 2015;372:2285-95.

For peer review only

For peer review only - http://bmjopen.bmj.com/site/about/guidelines.xhtml

7. Jovin TG, Chamorro A, Cobo E, de Miquel MA, Molina CA, Rovira A, San Roman L, Serena J, Abilleira S, Ribo M, Millan M, Urra X, Cardona P, Lopez-Cancio E, Tomasello A, Castano C, Blasco J, Aja L, Dorado L, Quesada H, Rubiera M, Hernandez-Perez M, Goyal M, Demchuk AM, von Kummer R, Gallofre M, Davalos A and Investigators RT. Thrombectomy within 8 hours after symptom onset in ischemic stroke. *N Engl J Med*. 2015;372:2296-306.

8. Muir KW, Ford GA, Messow CM, Ford I, Murray A, Clifton A, Brown MM, Madigan J, Lenthall R, Robertson F, Dixit A, Cloud GC, Wardlaw J, Freeman J, White P and Investigators P. Endovascular therapy for acute ischaemic stroke: the Pragmatic Ischaemic Stroke Thrombectomy Evaluation (PISTE) randomised, controlled trial. *J Neurol Neurosurg Psychiatry*. 2017;88:38-44.

9. Bracard S, Ducrocq X, Mas JL, Soudant M, Oppenheim C, Moulin T, Guillemin F and investigators T. Mechanical thrombectomy after intravenous alteplase versus alteplase alone after stroke (THRACE): a randomised controlled trial. *Lancet Neurol*. 2016;15:1138-47.

10. Goyal M, Menon BK, van Zwam WH, Dippel DW, Mitchell PJ, Demchuk AM, Davalos A, Majoie CB, van der Lugt A, de Miquel MA, Donnan GA, Roos YB, Bonafe A, Jahan R, Diener HC, van den Berg LA, Levy EI, Berkhemer OA, Pereira VM, Rempel J, Millan M, Davis SM, Roy D, Thornton J, Roman LS, Ribo M, Beumer D, Stouch B, Brown S, Campbell BC, van Oostenbrugge RJ, Saver JL, Hill MD, Jovin TG and collaborators H. Endovascular thrombectomy after large-vessel ischaemic stroke: a meta-analysis of individual patient data from five randomised trials. *Lancet*. 2016;387:1723-31.

11. Meretoja A, Keshtkaran M, Saver JL, Tatlisumak T, Parsons MW, Kaste M, Davis SM, Donnan GA and Churilov L. Stroke thrombolysis: save a minute, save a day. *Stroke*. 2014;45:1053-8.

12. Meretoja A, Keshtkaran M, Tatlisumak T, Donnan GA and Churilov L. Endovascular therapy for ischemic stroke: Save a minute-save a week. *Neurology*. 2017;88:2123-2127.

13. Emberson J, Lees KR, Lyden P, Blackwell L, Albers G, Bluhmki E, Brott T, Cohen G, Davis S, Donnan G, Grotta J, Howard G, Kaste M, Koga M, von Kummer R, Lansberg M, Lindley RI, Murray G, Olivot JM, Parsons M, Tilley B, Toni D, Toyoda K, Wahlgren N, Wardlaw J, Whiteley W, del Zoppo GJ, Baigent C, Sandercock P, Hacke W and Stroke Thrombolysis Trialists' Collaborative G. Effect of treatment delay, age, and stroke severity on the effects of intravenous thrombolysis with alteplase for acute ischaemic stroke: a meta-analysis of individual patient data from randomised trials. *Lancet*. 2014;384:1929-35.

14. Wahlgren N, Moreira T, Michel P, Steiner T, Jansen O, Cognard C, Mattle HP, van Zwam W, Holmin S, Tatlisumak T, Petersson J, Caso V, Hacke W, Mazighi M, Arnold M, Fischer U, Szikora I, Pierot L, Fiehler J, Gralla J, Fazekas F, Lees KR, Eso-Ksu EEE and Ean. Mechanical thrombectomy in acute ischemic stroke: Consensus statement by ESO-Karolinska Stroke Update 2014/2015, supported by ESO, ESMINT, ESNR and EAN. *Int J Stroke*. 2016;11:134-47.

15. Powers WJ, Rabinstein AA, Ackerson T, Adeoye OM, Bambakidis NC, Becker K, Biller J, Brown M, Demaerschalk BM, Hoh B, Jauch EC, Kidwell CS, Leslie-Mazwi TM, Ovbiagele B, Scott PA, Sheth KN, Southerland AM, Summers DV, Tirschwell DL and American Heart Association Stroke C. 2018 Guidelines for the Early Management of Patients With Acute Ischemic Stroke: A Guideline for Healthcare Professionals From the American Heart Association/American Stroke Association. *Stroke*. 2018;49:e46-e110.

16. Turc G, Bhogal P, Fischer U, Khatri P, Lobotesis K, Mazighi M, Schellinger PD, Toni D, de Vries J, White P and Fiehler J. European Stroke Organisation (ESO) - European Society for Minimally Invasive Neurological Therapy (ESMINT) Guidelines on Mechanical Thrombectomy in Acute Ischaemic StrokeEndorsed by Stroke Alliance for Europe (SAFE). *Eur Stroke J*. 2019;4:6-12.

17. Nogueira RG, Jadhav AP, Haussen DC, Bonafe A, Budzik RF, Bhuva P, Yavagal DR, Ribo M, Cognard C, Hanel RA, Sila CA, Hassan AE, Millan M, Levy EI, Mitchell P, Chen M, English JD, Shah QA, Silver FL, Pereira VM, Mehta BP, Baxter BW, Abraham MG, Cardona P, Veznedaroglu E, Hellinger FR, Feng L, Kirmani JF, Lopes DK, Jankowitz BT, Frankel MR, Costalat V, Vora NA, Yoo AJ, Malik AM, Furlan AJ, Rubiera M, Aghaebrahim A, Olivot JM, Tekle WG, Shields R, Graves T, Lewis RJ, Smith WS, Liebeskind DS,

For peer review only - http://bmjopen.bmj.com/site/about/guidelines.xhtml

Saver JL, Jovin TG and Investigators DT. Thrombectomy 6 to 24 Hours after Stroke with a Mismatch between Deficit and Infarct. *N Engl J Med*. 2018;378:11-21.

18. Albers GW, Marks MP, Kemp S, Christensen S, Tsai JP, Ortega-Gutierrez S, McTaggart RA, Torbey MT, Kim-Tenser M, Leslie-Mazwi T, Sarraj A, Kasner SE, Ansari SA, Yeatts SD, Hamilton S, Mlynash M, Heit JJ, Zaharchuk G, Kim S, Carrozzella J, Palesch YY, Demchuk AM, Bammer R, Lavori PW, Broderick JP, Lansberg MG and Investigators D. Thrombectomy for Stroke at 6 to 16 Hours with Selection by Perfusion Imaging. *N Engl J Med*. 2018;378:708-718.

19. Goyal L, Govindan A, Sheth RA, Nardi V, Blaszkowsky LS, Faris JE, Clark JW, Ryan DP, Kwak EL, Allen JN, Murphy JE, Saha SK, Hong TS, Wo JY, Ferrone CR, Tanabe KK, Chong DQ, Deshpande V, Borger DR, Iafrate AJ, Bardeesy N, Zheng H and Zhu AX. Prognosis and Clinicopathologic Features of Patients With Advanced Stage Isocitrate Dehydrogenase (IDH) Mutant and IDH Wild-Type Intrahepatic Cholangiocarcinoma. *Oncologist*. 2015;20:1019-27.

20. Scheitz JF, Gensicke H, Zinkstok SM, Curtze S, Arnold M, Hametner C, Pezzini A, Turc G, Zini A, Padjen V, Wegener S, Nordanstig A, Kellert L, Kagi G, Bejot Y, Michel P, Leys D, Nolte CH, Nederkoorn PJ, Engelter ST and collaboration T. Cohort profile: Thrombolysis in Ischemic Stroke Patients (TRISP): a multicentre research collaboration. *BMJ Open*. 2018;8:e023265.

21. Engelter ST, Soinne L, Ringleb P, Sarikaya H, Bordet R, Berrouschot J, Odier C, Arnold M, Ford GA, Pezzini A, Zini A, Rantanen K, Rocco A, Bonati LH, Kellert L, Strbian D, Stoll A, Meier N, Michel P, Baumgartner RW, Leys D, Tatlisumak T and Lyrer PA. IV thrombolysis and statins. *Neurology*. 2011;77:888-95.

22. Waje-Andreassen U, Nabavi DG, Engelter ST, Dippel DW, Jenkinson D, Skoda O, Zini A, Orken DN, Staikov I and Lyrer P. European Stroke Organisation certification of stroke units and stroke centres. *Eur Stroke J*. 2018;3:220-226.

23. Zaidat OO, Yoo AJ, Khatri P, Tomsick TA, von Kummer R, Saver JL, Marks MP, Prabhakaran S, Kallmes DF, Fitzsimmons BF, Mocco J, Wardlaw JM, Barnwell SL, Jovin TG, Linfante I, Siddiqui AH, Alexander MJ, Hirsch JA, Wintermark M, Albers G, Woo HH, Heck DV, Lev M, Aviv R, Hacke W, Warach S, Broderick J, Derdeyn CP, Furlan A, Nogueira RG, Yavagal DR, Goyal M, Demchuk AM, Bendszus M, Liebeskind DS, Cerebral Angiographic Revascularization Grading C, group SRw and Force STiCIT. Recommendations on angiographic revascularization grading standards for acute ischemic stroke: a consensus statement. *Stroke*. 2013;44:2650-63.

24. Hacke W, Kaste M, Fieschi C, von Kummer R, Davalos A, Meier D, Larrue V, Bluhmki E, Davis S, Donnan G, Schneider D, Diez-Tejedor E and Trouillas P. Randomised double-blind placebo-controlled trial of thrombolytic therapy with intravenous alteplase in acute ischaemic stroke (ECASS II). Second European-Australasian Acute Stroke Study Investigators. *Lancet*. 1998;352:1245-51.

25. National Institute of Neurological D and Stroke rt PASSG. Tissue plasminogen activator for acute ischemic stroke. *N Engl J Med*. 1995;333:1581-7.

26. Wahlgren N, Ahmed N, Davalos A, Ford GA, Grond M, Hacke W, Hennerici MG, Kaste M, Kuelkens S, Larrue V, Lees KR, Roine RO, Soinne L, Toni D, Vanhooren G and investigators S-M. Thrombolysis with alteplase for acute ischaemic stroke in the Safe Implementation of Thrombolysis in Stroke-Monitoring Study (SITS-MOST): an observational study. *Lancet*. 2007;369:275-82.

27. Neuberger U, Mohlenbruch MA, Herweh C, Ulfert C, Bendszus M and Pfaff J. Classification of Bleeding Events: Comparison of ECASS III (European Cooperative Acute Stroke Study) and the New Heidelberg Bleeding Classification. *Stroke*. 2017;48:1983-1985.

28. Tatlisumak T. Implication of the recent positive endovascular intervention trials for organizing acute stroke care: European perspective. *Stroke*. 2015;46:1468-73.

29. Liu X, Xu G, Liu Y, Zhu W, Ma M, Xiong Y, Zi W, Dai Q, Leung T, Yan B, Davis S, Liebeskind DS, Pereira VM, Nogueira RG and Investigators BT. Acute basilar artery occlusion: Endovascular Interventions

For peer review only

For peer review only - http://bmjopen.bmj.com/site/about/guidelines.xhtml

versus Standard Medical Treatment (BEST) Trial-Design and protocol for a randomized, controlled, multicenter study. *Int J Stroke*. 2017;12:779-785.
30. van der Hoeven EJ, Schonewille WJ, Vos JA, Algra A, Audebert HJ, Berge E, Ciccone A, Mazighi M, Michel P, Muir KW, Obach V, Puetz V, Wijman CA, Zini A, Kappelle JL and Group BS. The Basilar Artery International Cooperation Study (BASICS): study protocol for a randomised controlled trial. *Trials*. 2013;14:200.
31. Singer OC, Berkefeld J, Nolte CH, Bohner G, Haring HP, Trenkler J, Groschel K, Muller-Forell W, Niederkorn K, Deutschmann H, Neumann-Haefelin T, Hohmann C, Bussmeyer M, Mpotsaris A, Stoll A, Bormann A, Brenck J, Schlamann MU, Jander S, Turowski B, Petzold GC, Urbach H, Liebeskind DS and Group ES. Mechanical recanalization in basilar artery occlusion: the ENDOSTROKE study. *Ann Neurol*. 2015;77:415-24.
32. Singer OC, Haring HP, Trenkler J, Nolte CH, Bohner G, Neumann-Haefelin T, Hofmann E, Reich A, Wiesmann M, Niederkorn K, Deutschmann H, Bussmeyer M, Mpotsaris A, Stoll A, Bormann A, Petzold GC, Urbach H, Jander S, Turowski B, Weimar C, Schlamann M, Groschel K, Boor S, Berkefeld J and Group ES. Periprocedural aspects in mechanical recanalization for acute stroke: data from the ENDOSTROKE registry. *Neuroradiology*. 2013;55:1143-51.
33. Wollenweber FA, Tiedt S, Alegiani A, Alber B, Bangard C, Berrouschot J, Bode FJ, Boeckh-Behrens T, Bohner G, Bormann A, Braun M, Dorn F, Eckert B, Flottmann F, Hamann GF, Henn KH, Herzberg M, Kastrup A, Kellert L, Kraemer C, Krause L, Lehm M, Liman J, Lowens S, Mpotsaris A, Papanagiotou P, Petersen M, Petzold GC, Pfeilschifter W, Psychogios MN, Reich A, von Rennenberg R, Rother J, Schafer JH, Siebert E, Siedow A, Solymosi L, Thonke S, Wagner M, Wunderlich S, Zweynert S, Nolte CH, Gerloff C, Thomalla G, Dichgans M and Fiehler J. Functional Outcome Following Stroke Thrombectomy in Clinical Practice. *Stroke*. 2019;50:2500-2506.
34. Heuschmann PU, Biegler MK, Busse O, Elsner S, Grau A, Hasenbein U, Hermanek P, Janzen RW, Kolominsky-Rabas PL, Kraywinkel K, Lowitzsch K, Misselwitz B, Nabavi DG, Otten K, Pientka L, von Reutern GM, Ringelstein EB, Sander D, Wagner M and Berger K. Development and implementation of evidence-based indicators for measuring quality of acute stroke care: the Quality Indicator Board of the German Stroke Registers Study Group (ADSR). *Stroke*. 2006;37:2573-8.
35. group ISTc, Sandercock P, Wardlaw JM, Lindley RI, Dennis M, Cohen G, Murray G, Innes K, Venables G, Czlonkowska A, Kobayashi A, Ricci S, Murray V, Berge E, Slot KB, Hankey GJ, Correia M, Peeters A, Matz K, Lyrer P, Gubitz G, Phillips SJ and Arauz A. The benefits and harms of intravenous thrombolysis with recombinant tissue plasminogen activator within 6 h of acute ischaemic stroke (the third international stroke trial [IST-3]): a randomised controlled trial. *Lancet*. 2012;379:2352-63.
36. Gensicke H, Strbian D, Zinkstok SM, Scheitz JF, Bill O, Hametner C, Moulin S, Zini A, Kagi G, Pezzini A, Padjen V, Bejot Y, Corbiere S, Zonneveld TP, Seiffge DJ, Roos YB, Traenka C, Putaala J, Peters N, Bonati LH, Curtze S, Erdur H, Sibolt G, Koch P, Vandelli L, Ringleb P, Leys D, Cordonnier C, Michel P, Nolte CH, Lyrer PA, Tatlisumak T, Nederkoorn PJ, Engelter ST and Thrombolysis in Stroke Patients C. Intravenous Thrombolysis in Patients Dependent on the Daily Help of Others Before Stroke. *Stroke*. 2016;47:450-6.
37. Zinkstok SM, Vergouwen MD, Engelter ST, Lyrer PA, Bonati LH, Arnold M, Mattle HP, Fischer U, Sarikaya H, Baumgartner RW, Georgiadis D, Odier C, Michel P, Putaala J, Griebe M, Wahlgren N, Ahmed N, van Geloven N, de Haan RJ and Nederkoorn PJ. Safety and functional outcome of thrombolysis in dissection-related ischemic stroke: a meta-analysis of individual patient data. *Stroke*. 2011;42:2515-20.
38. Gensicke H, Zinkstok SM, Roos YB, Seiffge DJ, Ringleb P, Artto V, Putaala J, Haapaniemi E, Leys D, Bordet R, Michel P, Odier C, Berrouschot J, Arnold M, Heldner MR, Zini A, Bigliardi G, Padjen V, Peters N, Pezzini A, Schindler C, Sarikaya H, Bonati LH, Tatlisumak T, Lyrer PA, Nederkoorn PJ and Engelter ST. IV thrombolysis and renal function. *Neurology*. 2013;81:1780-8.

For peer review only - http://bmjopen.bmj.com/site/about/guidelines.xhtml

39. Gensicke H, Wicht A, Bill O, Zini A, Costa P, Kagi G, Stark R, Seiffge DJ, Traenka C, Peters N, Bonati LH, Giovannini G, De Marchis GM, Poli L, Polymeris A, Vanacker P, Sarikaya H, Lyrer PA, Pezzini A, Vandelli L, Michel P, Engelter ST and Thrombolysis in Stroke Patients c. Impact of body mass index on outcome in stroke patients treated with intravenous thrombolysis. *Eur J Neurol*. 2016;23:1705-1712.

40. Scheitz JF, Seiffge DJ, Tutuncu S, Gensicke H, Audebert HJ, Bonati LH, Fiebach JB, Tranka C, Lyrer PA, Endres M, Engelter ST and Nolte CH. Dose-related effects of statins on symptomatic intracerebral hemorrhage and outcome after thrombolysis for ischemic stroke. *Stroke*. 2014;45:509-14.

41. Zinkstok SM, Engelter ST, Gensicke H, Lyrer PA, Ringleb PA, Artto V, Putaala J, Haapaniemi E, Tatlisumak T, Chen Y, Leys D, Sarikaya H, Michel P, Odier C, Berrouschot J, Arnold M, Heldner MR, Zini A, Fioravanti V, Padjen V, Beslac-Bumbasirevic L, Pezzini A, Roos YB and Nederkoorn PJ. Safety of thrombolysis in stroke mimics: results from a multicenter cohort study. *Stroke*. 2013;44:1080-4.

42. Seiffge DJ, Hooff RJ, Nolte CH, Bejot Y, Turc G, Ikenberg B, Berge E, Persike M, Dequatre-Ponchelle N, Strbian D, Pfeilschifter W, Zini A, Tveiten A, Naess H, Michel P, Sztajzel R, Luft A, Gensicke H, Traenka C, Hert L, Scheitz JF, De Marchis GM, Bonati LH, Peters N, Charidimou A, Werring DJ, Palm F, Reinhard M, Niesen WD, Nagao T, Pezzini A, Caso V, Nederkoorn PJ, Kagi G, von Hessling A, Padjen V, Cordonnier C, Erdur H, Lyrer PA, Brouns R, Steiner T, Tatlisumak T, Engelter ST and Group* NS. Recanalization therapies in acute ischemic stroke patients: impact of prior treatment with novel oral anticoagulants on bleeding complications and outcome. *Circulation*. 2015;132:1261-9.

43. Gensicke H, Al Sultan AS, Strbian D, Hametner C, Zinkstok SM, Moulin S, Bill O, Zini A, Padjen V, Kagi G, Pezzini A, Seiffge DJ, Traenka C, Raty S, Amiri H, Zonneveld TP, Lachenmeier R, Polymeris A, Roos YB, Gumbinger C, Jovanovic DR, Curtze S, Sibolt G, Vandelli L, Ringleb PA, Leys D, Cordonnier C, Michel P, Lyrer PA, Peters N, Tatlisumak T, Nederkoorn PJ, Engelter ST and Thrombolysis in Stroke Patients C. Intravenous thrombolysis and platelet count. *Neurology*. 2018;90:e690-e697.

44. Scheitz JF, Turc G, Kujala L, Polymeris AA, Heldner MR, Zonneveld TP, Erdur H, Curtze S, Traenka C, Breniere C, Wiest R, Rocco A, Sibolt G, Gensicke H, Endres M, Martinez-Majander N, Bejot Y, Nederkoorn PJ, Oppenheim C, Arnold M, Engelter ST, Strbian D, Nolte CH and Collaboration T. Intracerebral Hemorrhage and Outcome After Thrombolysis in Stroke Patients Using Selective Serotonin-Reuptake Inhibitors. *Stroke*. 2017;48:3239-3244.

45. Polymeris AA, Curtze S, Erdur H, Hametner C, Heldner MR, Groot AE, Zini A, Bejot Y, Dietrich A, Martinez-Majander N, von Rennenberg R, Gumbinger C, Schaedelin S, De Marchis GM, Thilemann S, Traenka C, Lyrer PA, Bonati LH, Wegener S, Ringleb PA, Tatlisumak T, Nolte CH, Scheitz JF, Arnold M, Strbian D, Nederkoorn PJ, Gensicke H, Engelter ST and Collaborators T. Intravenous Thrombolysis for Suspected Ischemic Stroke with Seizure at Onset. *Ann Neurol*. 2019.

46. Strbian D, Ringleb P, Michel P, Breuer L, Ollikainen J, Murao K, Seiffge DJ, Jung S, Obach V, Weder B, Eskandari A, Gensicke H, Chamorro A, Mattle HP, Engelter S, Leys D, Numminen H, Kohrmann M, Hacke W and Tatlisumak T. Ultra-early intravenous stroke thrombolysis: do all patients benefit similarly? *Stroke*. 2013;44:2913-6.

47. Strbian D, Michel P, Ringleb P, Numminen H, Breuer L, Bodenant M, Seiffge DJ, Jung S, Obach V, Weder B, Tiainen M, Eskandari A, Gumbinger C, Gensicke H, Chamorro A, Mattle HP, Engelter ST, Leys D, Kohrmann M, Parkkila AK, Hacke W and Tatlisumak T. Relationship between onset-to-door time and door-to-thrombolysis time: a pooled analysis of 10 dedicated stroke centers. *Stroke*. 2013;44:2808-13.

48. Zonneveld TP, Curtze S, Zinkstok SM, Gensicke H, Moulin S, Scheitz JF, Seiffge DJ, Hametner C, Heldner MR, Traenka C, Erdur H, Baharoglu I, Martinez-Majander N, Pezzini A, Zini A, Padjen V, Correia PN, Strbian D, Michel P, Bejot Y, Arnold M, Leys D, Ringleb PA, Tatlisumak T, Nolte CH, Engelter ST, Nederkoorn PJ and collaborators T. Non-office-hours admission affects intravenous thrombolysis treatment times and clinical outcome. *J Neurol Neurosurg Psychiatry*. 2018;89:1005-1007.

For peer review only - http://bmjopen.bmj.com/site/about/guidelines.xhtml

49. Strbian D, Engelter S, Michel P, Meretoja A, Sekoranja L, Ahlhelm FJ, Mustanoja S, Kuzmanovic I, Sairanen T, Forss N, Cordier M, Lyrer P, Kaste M and Tatlisumak T. Symptomatic intracranial hemorrhage after stroke thrombolysis: the SEDAN score. *Ann Neurol*. 2012;71:634-41.
50. Strbian D, Meretoja A, Ahlhelm FJ, Pitkaniemi J, Lyrer P, Kaste M, Engelter S and Tatlisumak T. Predicting outcome of IV thrombolysis-treated ischemic stroke patients: the DRAGON score. *Neurology*. 2012;78:427-32.
51. Strbian D, Seiffge DJ, Breuer L, Numminen H, Michel P, Meretoja A, Coote S, Bordet R, Obach V, Weder B, Jung S, Caso V, Curtze S, Ollikainen J, Lyrer PA, Eskandari A, Mattle HP, Chamorro A, Leys D, Bladin C, Davis SM, Kohrmann M, Engelter ST and Tatlisumak T. Validation of the DRAGON score in 12 stroke centers in anterior and posterior circulation. *Stroke*. 2013;44:2718-21.
52. Curtze S, Haapaniemi E, Melkas S, Mustanoja S, Putaala J, Sairanen T, Sibolt G, Tiainen M, Tatlisumak T and Strbian D. White Matter Lesions Double the Risk of Post-Thrombolytic Intracerebral Hemorrhage. *Stroke*. 2015;46:2149-55.
53. Barber PA, Demchuk AM, Zhang J and Buchan AM. Validity and reliability of a quantitative computed tomography score in predicting outcome of hyperacute stroke before thrombolytic therapy. ASPECTS Study Group. Alberta Stroke Programme Early CT Score. *Lancet*. 2000;355:1670-4.
54. Parsons M, Spratt N, Bivard A, Campbell B, Chung K, Miteff F, O'Brien B, Bladin C, McElduff P, Allen C, Bateman G, Donnan G, Davis S and Levi C. A randomized trial of tenecteplase versus alteplase for acute ischemic stroke. *N Engl J Med*. 2012;366:1099-107.
55. Campbell BCV, Mitchell PJ, Churilov L, Yassi N, Kleinig TJ, Dowling RJ, Yan B, Bush SJ, Dewey HM, Thijs V, Scroop R, Simpson M, Brooks M, Asadi H, Wu TY, Shah DG, Wijeratne T, Ang T, Miteff F, Levi CR, Rodrigues E, Zhao H, Salvaris P, Garcia-Esperon C, Bailey P, Rice H, de Villiers L, Brown H, Redmond K, Leggett D, Fink JN, Collecutt W, Wong AA, Muller C, Coulthard A, Mitchell K, Clouston J, Mahady K, Field D, Ma H, Phan TG, Chong W, Chandra RV, Slater LA, Krause M, Harrington TJ, Faulder KC, Steinfort BS, Bladin CF, Sharma G, Desmond PM, Parsons MW, Donnan GA, Davis SM and Investigators E-IT. Tenecteplase versus Alteplase before Thrombectomy for Ischemic Stroke. *N Engl J Med*. 2018;378:1573-1582.
56. Ma H, Campbell BCV, Parsons MW, Churilov L, Levi CR, Hsu C, Kleinig TJ, Wijeratne T, Curtze S, Dewey HM, Miteff F, Tsai CH, Lee JT, Phan TG, Mahant N, Sun MC, Krause M, Sturm J, Grimley R, Chen CH, Hu CJ, Wong AA, Field D, Sun Y, Barber PA, Sabet A, Jannes J, Jeng JS, Clissold B, Markus R, Lin CH, Lien LM, Bladin CF, Christensen S, Yassi N, Sharma G, Bivard A, Desmond PM, Yan B, Mitchell PJ, Thijs V, Carey L, Meretoja A, Davis SM, Donnan GA and Investigators E. Thrombolysis Guided by Perfusion Imaging up to 9 Hours after Onset of Stroke. *N Engl J Med*. 2019;380:1795-1803.
57. Warach SJ, Luby M, Albers GW, Bammer R, Bivard A, Campbell BC, Derdeyn C, Heit JJ, Khatri P, Lansberg MG, Liebeskind DS, Majoie CB, Marks MP, Menon BK, Muir KW, Parsons MW, Vagal A, Yoo AJ, Alexandrov AV, Baron JC, Fiorella DJ, Furlan AJ, Puig J, Schellinger PD, Wintermark M, Stroke Imaging R and Investigators VI-I. Acute Stroke Imaging Research Roadmap III Imaging Selection and Outcomes in Acute Stroke Reperfusion Clinical Trials: Consensus Recommendations and Further Research Priorities. *Stroke*. 2016;47:1389-98.

For peer review only - http://bmjopen.bmj.com/site/about/guidelines.xhtml

Table 1. Universal standards and requirements for the databases and centers contributing to the EVA-TRISP registry*.

- Prospective registry of consecutive patients with systematic check-up of missing cases.
- Comprehensive collection of baseline characteristics according to consensus definitions stated in this and the previous methodology papers.
- Prospective assessment of hemorrhagic complications (symptomatic intracerebral hemorrhage according to ECASS II criteria) and functional outcome at 3 and 12 months (according to the modified Rankin Scale; either telephone interview, postal questionnaire, or follow-up visit).
- Approval of institutional review board to maintain the respective EVT database and to obtain 3- and 12-month follow-up data.
- EVA-TRISP centers are comprehensive stroke centers with high-volume EVT applications - typically university hospitals or closely affiliated to university hospitals.
- Treatment of acute ischemic stroke patients with EVT according to guidelines valid at the relevant time or documentation of deviation therefrom.

* EVA-TRISP welcomes participation and project proposals of further centers fulfilling the commitment and the outlined requirements.

For peer review only

For peer review only - http://bmjopen.bmj.com/site/about/guidelines.xhtml

Table 2. EVA-TRISP centers, time period, number of endovascular treatments done, and population-base for EVT (in alphabetical order).

City	No of stroke EVT (Jan 2015 to Dec 2019)
Amsterdam	864
Basel	413
Belgrade	136[1]
Berlin	480[2]
Bern	1422
Bremen	Estimation: 200/year
Brescia	412
Bologna	395
Goettingen	396
Gothenburg	1097
Heidelberg	1500
Helsinki	796[2]
Jerusalem	249
Larissa	-
Lausanne	732
Lille	1806
Modena	489
Munich	600
St. Gallen	490
Zurich	Estimation: 500

[1]Jan 2018-Dec 2019

[2]Nov 2015-Dec 2019

For peer review only

For peer review only - http://bmjopen.bmj.com/site/about/guidelines.xhtml

Figure 1. EVA-TRISP centers.

For peer review only

For peer review only - http://bmjopen.bmj.com/site/about/guidelines.xhtml

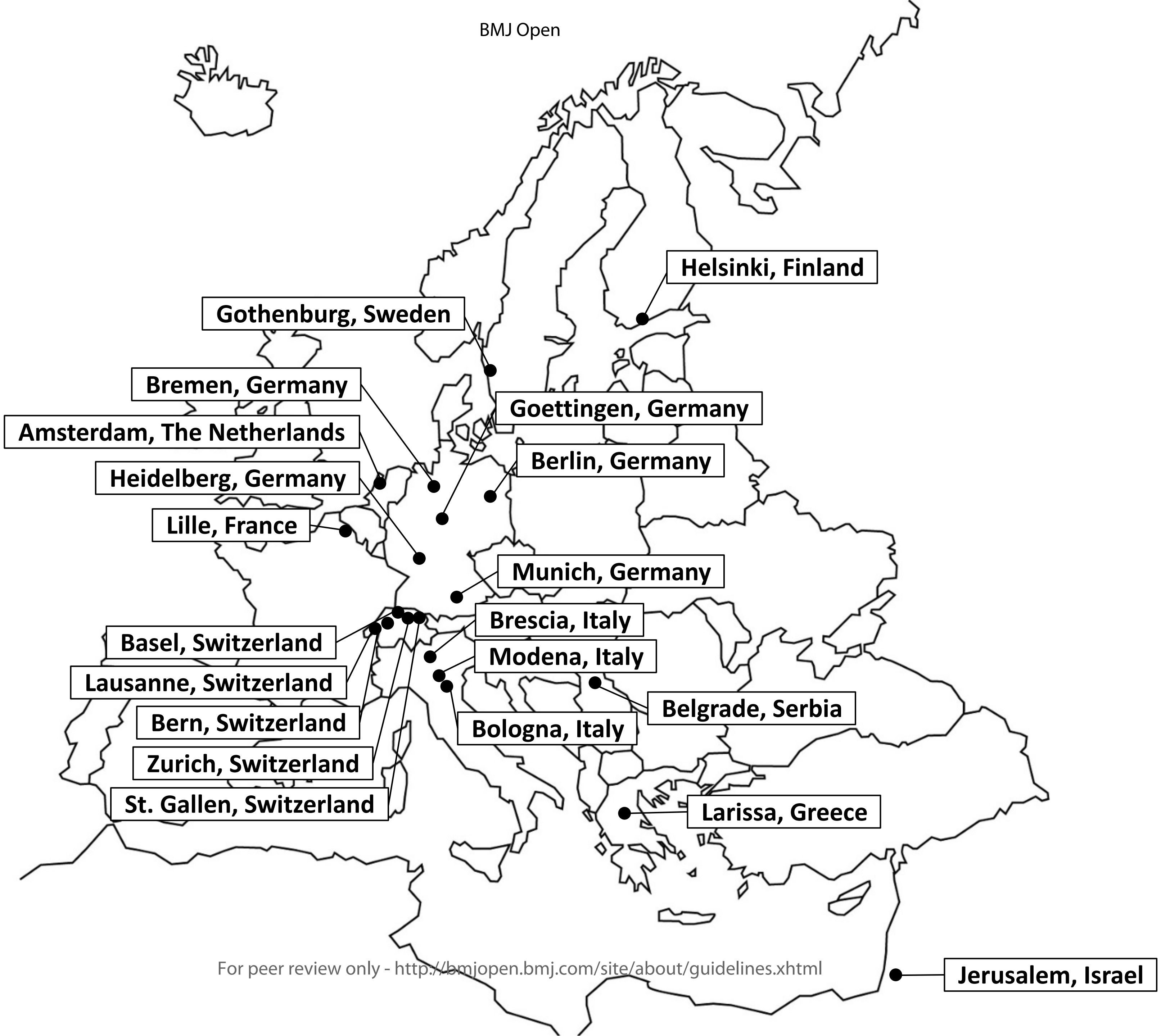
Helsinki, Finland
Gothenburg, Sweden
Bremen, Germany
Goettingen, Germany
Amsterdam, The Netherlands
Berlin, Germany
Heidelberg, Germany
Lille, France
Munich, Germany
Brescia, Italy
Basel, Switzerland
Modena, Italy
Lausanne, Switzerland
Belgrade, Serbia
Bern, Switzerland
Bologna, Italy
Zurich, Switzerland
St. Gallen, Switzerland
Larissa, Greece
Jerusalem, Israel

APPENDIX 1: EVA-TRISP Investigators (in alphabetical order by country)

Finland (1): Sami Curtze, Kimmo Lappalainen, Nicolas Martinez-Majander, Jukka Putaala, Gerli Sibolt, Daniel Strbian, Silja Räty, Turgut Tatlisumak, Marjaana Tiainen (Helsinki University Hospital)

France (1): Nicolas Bricout, Marie Bodenant, Regis Bordet, Charlotte Cordonnier, Nelly Dequatre, Hilde Hénon, Didier Leys, Anne-Marie Mendyk (University Lille North de France)

Germany (5): Andreas Kastrup, Panagiotis Papanagiotou (University Hospitals Bremen-Mitte and Bremen-Ost); Tim-Bastian Braemswig, Hebun Erdur, Christian H Nolte, Regina von Rennenberg, Jan F Scheitz, Georg Bohner (Charité-Universitätsmedizin, Berlin); Alex Brehm, Jan Liman, Marios Psychogios (University Medical Center Goettingen); Martin Bendszus, Christian Hametner, Markus Möhlenbruch, Peter A Ringleb (University Hospital Heidelberg); Katharina Feil, Lars Kellert, Clemens Küpper (University Hospital Munich LMU)

Grecce (1): George Ntaios, Dimitrios Sagris, Ioannis Ioannidis George Karagiorgas, Eftychia Kapsalaki, Marianna Vlychou (University of Thessaly)

Israel (1): Jose Cohen, John Gomori, Ronen R Leker (Hadassah-Hebrew University Medical Center, Jerusalem)

Italy (3): Mauro Magoni, Alessandro Pezzini (University Hospital Brescia); Guido Bigliardi, Luca Verganti, Stefano Vallone, Stefania Maffei (University Hospital Modena), Andrea Zini,

For peer review only

For peer review only - http://bmjopen.bmj.com/site/about/guidelines.xhtml

Luigi Simonetti, Mauro Gentile, Luigi Cirillo, Ludovica Migliaccio (IRCCS Istituto di Scienze Neurologiche di Bologna)

The Netherlands (1): M Irem Baharoglu, Sophie van den Berg, Paul J Nederkoorn, Yvo B Roos, Fianne HM Spaander, Sanne M Zinkstok, Thomas P Zonneveld, Charles Majoie (Academic Medical Center Amsterdam)

Serbia (1): Mirjana Arsenijevic, Ivana Berisavac, Marko Ercegovac, Dejana R Jovanovic, Visnja Padjen, Predrag Stanarcevic, Maja Stefanovic Budimkic, Tamara Svabic Medjedovic, Ivan Vukasinovic, Vladimir Cvetic (Clinical Centre of Serbia, Belgrade)

Sweden (1): Margareta Abrahamson, Arne Allard, Monica Argus, Anke Brederleu, Erik Ceder, Maria Davidson, Niclas Dehlfors, Dennis Dunker, Torsteinn Gunnarsson, Lukas Holmegaard, Mikael Jerndal, Susanna Johansson, Katarina Jood, Camilla Karlsson, Jan-Erik Karlsson, Birgitta Leiram, Miroslav Malac, Inger Nilsson, Annika Nordanstig, Petra Redfors, Alexandros Rentzos, Turgut Tatlisumak (Sahlgrenska University Hospital, Gothenburg)

Switzerland (5): Leo H Bonati, Stefan T Engelter, Joachim Fladt, Henrik Gensicke, Philippe A Lyrer, Gian Marco De Marchis, Nils Peters, Alexandros Polymeris, Sebastian Thilemann, Christopher Traenka (University Hospital Basel); Marcel Arnold, Urs Fischer, Jan Gralla, Mirjam R Heldner, Hakan Sarikaya, David J Seiffge, Roland Wiest (University Hospital Bern); Olivier Bill, Ashraf Eskandari, Patrik Michel, Gaia Sirimarco (Hospitalier Universitaire Vaudois, Lausanne); Georg Kägi, Johannes Weber (Kantonsspital St. Gallen); Zsolt Kulcsar, Andreas R Luft, Susanne Wegener (University Hospital Zurich)

For peer review only - http://bmjopen.bmj.com/site/about/guidelines.xhtml
For peer review only

APPENDIX 2. EVA-TRISP DATABASE DOMAINS AND ITEMS

The exact list of available variables may slightly differ between centers according to the judgment of local ethics committees.

Patient history

- Age
- Sex
- Pre-stroke mRS
- Independent prior to stroke (Y/N)
- Risk factor atrial fibrillation present and known (Y/N)
- Risk factor diabetes present and known (Y/N) (Y/N)
- Risk factor hypertension present and known(Y/N)
- Risk factor hypercholesterolemia present and known (Y/N)
- Risk factor coronary artery disease present and known(Y/N)
- Risk factor prior ischemic stroke (clinical diagnosis) (Y/N)
- If prior ischemic stroke, prior treatment with IVT/EVT (Y/N)
- Risk factor current smoking (or stopped <2y) (Y/N
- Pre-IVT use of statins (Y/N as well as name and dosage)
- Pre-IVT use of antihypertensive (Y/N as well as name and dosage)
- Pre-IVT use of antiplatelets (Y/N as well as name and dosage)
- Pre-IVT use of anticoagulants (Y/N as well as name and dosage)
- All medications (name and dosage as free text)

For peer review only - http://bmjopen.bmj.com/site/about/guidelines.xhtml

For peer review only

Pre-hospital

- Date of stroke onset (last seen well)
- Time of stroke onset (last seen well)
- Date of stroke onset (first seen sick)
- Time of stroke onset (first seen sick)
- Stroke onset witnessed? (Y/N)
- Wake-up stroke? (Y/N)
- Epileptic seizure at stroke onset (Y/N)

Admission

- Date of hospital arrival
- Time of hospital arrival
- Was patient transferred from another hospital? (Y/N)
- IVT administrated at other hospital? (Y/N)
- NIHSS at admission
- Systolic blood pressure on admission [mmHg]
- Diastolic blood pressure on admission [mmHg]
- Weight (exact / estimated)
- Height (exact / estimated)
- Glucose on admission [mmol/l]
- Creatinine on admission [umol/l]

For peer review only - http://bmjopen.bmj.com/site/about/guidelines.xhtml

- International Normalized Ratio prior to IVT (point of care test / actual lab value)
- If direct oral anticoagulants (DOAC), specific essay/level of DOAC
- Platelets on admission x10e9 [/l]
- Hemoglobin on admission [g/dl]
- Leukocytes on admission x10e9 [/l]
- C-Reactive Protein on admission [mg/l]

Acute interventions

- Type of intervention (IVT, bridging, EVT)
- Date of IVT administration
- Time of IVT administration
- Dosage of rtPA IVT (0.6 mg/kg, 0.9 mg/kg, <50% of dose or complete)
- Angioedema related to IVT (Y/N)
- Exclusion criteria IVT (received IVT, delays, major prestroke handicap, imaging contraindication, bleeding risk, stroke missed/uncertain, other)
- Exclusion criteria IVT [free text]
- Date of EVT groin puncture
- Time of EVT groin puncture
- Intra-arterial thrombolytic drug (Y/N)
- Name and dosage of intra-arterial thrombolytic drug [free text]
- General anesthesia during EVT (Y/N)

For peer review only - http://bmjopen.bmj.com/site/about/guidelines.xhtml
For peer review only

- Type of endovascular treatment (none, stent retriever, aspiration, distal retriever, distal aspiration, Balloon angioplasty, permanent intracranial stent, extracranial stent, other [combination possible]
- Number of attempts for each treatment
- Name of device(s)
- Tandem stenosis/occlusion present (Y/N)
- Extracranial thrombectomy (no, ICA, VA)
- Extracranial permanent stent (no, ICA, VA)
- Only attempt to perform EVT
- EVT stopped early because (initiated, but access-to-clot-problems, tried, but artery already recanalized, other)
- EVT stopped early reason [free text]
- EVT complications (no, vessel perforation, vasospasm, dissection, SAH/ICH, device detachment/misplacement, embolization to new territory, access-site complications, early reocclusion, other [free text] [combination possible]
- Time of EVT end of procedure

Imaging

- Type of baseline image (none, CT, MR)
- Date of baseline image
- Time of baseline image

For peer review only - http://bmjopen.bmj.com/site/about/guidelines.xhtml

- Territory of infarction (ICA, MCA, ACA, PCA, Cerebellum, Brainstem [combination possible]
- Side of infarction (left anterior circulation, right anterior circulation, posterior circulation [combination possible]
- Anterior circulation ASPECTS on baseline image 0-10
- Posterior circulation ASPECTS on baseline image 0-10
- Early ischemic changes in suspected area (focal parenchymal hypoattenuation, loss of gray-white matter differentiation, focal edema manifested by sulcal or ventricular effacement) (Y/N)
- Occluded vessel with hyperdense artery sign (-> column CD) (Y/N)
- Type of baseline angiography (none, CTA, MRA)
- Site of main intracranial occlusion on baseline angiography (none, ICA-I, ICA-L/T, prox M1, distal M1, M2, ACA, PCA, BA, VA)
- Other occlusion site, please specify [free text]
- Additional vessel occlusion (y/n)
- If yes, location of additional vessel occlusion
- In case of intracranial large vessel occlusion in the anterior circulation: collaterals on baseline angiography if assessable (TAN Score 0-3)
- Relevant stenosis (>50% NASCET)/occlusion of extracranial ICA on baseline angiography (Y/N)
- Relevant stenosis (>50% NASCET)/occlusion of extracranial VA on baseline angiography (Y/N)

For peer review only

For peer review only - http://bmjopen.bmj.com/site/about/guidelines.xhtml

- Type of baseline perfusion modality (none, CTP, MRP)
- Mismatch ratio according to local modalities (Y/N)
- Total perfusion lesion/infarct core mismatch ratio visually on baseline perfusion image (f.i. 1.2 = perfusion lesion is 20% larger than core) (number)DSA (Y/N)
- In case of intracranial large vessel occlusion in the anterior circulation: collaterals on DSA (ASITN/SIR grading)
- Complete recanalization on DSA (mTICI=2b/3) (Y/N)
- Date of recanalization on DSA
- Time of recanalization on DSA

Follow up

- Type of first follow-up native image (none, NCCT, MRI)
- Date of follow-up image
- Time of follow-up image
- Type of first follow-up vessel imaging (none, CTA, MRA-TOF, MRA-CE, ultrasound)
- Date of follow-up vessel imaging
- Time of follow-up vessel imaging
- Complete recanalization on follow-up vessel imaging (CTA/MRA/US) (Y/N)
- mTICI
- Any intracerebral hemorrhage (ICH)
- Fatal ICH
- Symptomatic ICH (ECASS-2 criteria) (Y/N)

For peer review only - http://bmjopen.bmj.com/site/about/guidelines.xhtml

- Other ICH (SAH, SDH, etc.) [free text]
- NIHSS after 24h
- mRS after 3 months
- Recurrent ischemic stroke or TIA within 3 months (no, TIA(s), stroke(s) (if TIA and stroke, count stroke)
- mRS after 1 year

Other

- Modified TOAST (LAA, CE (including PFO), SAO, other, more than one, undetermined, stroke mimic
- Rare specific stroke causes to be studied in more detail (cervical artery dissection, intracranial artery dissection, endocarditis, vasculitis, coagulopathies)

For peer review only

For peer review only - http://bmjopen.bmj.com/site/about/guidelines.xhtml

Appendix 3: Names of the ethics committees

City	Ethics committees
Amsterdam	Anonymized registry based research does not need ethical approval in The Netherlands
Basel	The ethics committee Basel (Ethikkommission Nordwest- und Zentralschweiz (EKNZ))
Belgrade	The ethics committee of Clinical Centre of Serbia
Berlin	The ethical review committee of the Charité–University Medicine Berlin
Bern	The cantonal ethics Committee Bern
Bremen	The local ethic committee of the Ärztekammer Bremen
Bologna	Local regulations do not require approval by the ethics board for observational studies using registry data in IRCCS (Institute for Treatment and Research)
Brescia	EC ASST spedali civili university hospital Brescia
Goettingen	The ethics committee in Goettingen approved it (No.: 16/2/16).
Gothenburg	The regional ethical board of Gothenburg
Heidelberg	Ethik kommission der Medizinischen Fakultät Heidelberg
Helsinki	Local regulations do not require approval by the ethics board for retrospective studies using registry data
Larissa	Participation in EVA-TRISP does not require ethics approval in Greece
Jerusalem	Hadassah Medical Organization (HMO) Jerusalem
Lausanne	The ethics commission for research on humans of the Canton of Vaud has approved the scientific use data from the ASTRAL registry
Lille	Comité de Protection des Personnes Nord Ouest IV Lille, France
Modena	The study was approved by local EC called “Comitato Etico Area Vasta Emilia Nord"
Munich	The ethics committee of the chamber of physicians at Ludwig-Maximilians University LMU Munich
St. Gallen	Each study project has been approved by the ethical committee
Zurich	The ethics commission Zurich, Switzerland.

For peer review only
